# Fossil record biases skew inferences of mammal trait evolution

**DOI:** 10.1038/s41467-026-77026-w

**Published:** 2026-08-24

**Authors:** Graciela Sotelo, Sofía Galván, Sara Gamboa, Adrián Castro-Insua, Alfio Alessandro Chiarenza, Emma M. Dunne, Lewis A. Jones, Marta Matamala-Pagès, Eduardo Méndez-Quintas, Adriana Oliver, Iván Rey-Rodríguez, Sara Varela

**Affiliations:** 1 Centro de Investigación Mariña, Departamento de Ecoloxía e Bioloxía Animal, Universidade de Vigo, Vigo, Spain; 2 Zentralmagazin Naturwissenschaftlicher Sammlungen, Martin Luther University Halle-Wittenberg, Halle (Saale), Germany; 3 Department of Earth Sciences, University College London, London, UK; 4 School of Natural Sciences, Trinity College Dublin, Dublin, Ireland; 5 Oportunius, Axencia Galega de Innovación, Santiago de Compostela, Spain

**Keywords:** Phylogenetics, Palaeontology, Biodiversity

## Abstract

The fossil record is key to elucidating past biodiversity dynamics, yet it remains incomplete and non-uniform, governed by factors such as sedimentation rates, species’ range and body size, and sampling effort. How these biases distort inferences on evolutionary trends remains poorly understood. Here, we simulate a future fossil record of present-day land mammals to quantify the extent to which real evolutionary patterns can be recovered from fragmentary fossil data. Species are filtered according to sediment availability, species’ range and body size, and spatial and taxonomic sampling biases under three preservation rates, and then systematically pruned from a baseline mammal phylogeny before analyses of body mass and diet evolution through phylogenetic comparative methods. We find that biological and taxonomic filters strongly skew these inferences, while spatial filters do not significantly distort them. Our results provide first-order estimates of how fossil record incompleteness can bias our interpretation of deep-time biodiversity change.

## Abstract (web summary)

Fossils document the evolution of life on Earth but represent only a small fraction of the species that ever existed. This study illustrates how common fossil record biases can render a distorted picture of past communities’ composition and change.

## Introduction

The fossil record provides primary empirical evidence of how life evolved on Earth, documenting long-term shifts in biodiversity and its response to abiotic and biotic regulators. However, this rich archive is inherently incomplete and non-uniform across space, time, environments, and taxa^1–4^. These biases compromise our assessment of past diversity dynamics^5–9^, despite the development of several methods to take them into account^10–13^. Recent approaches further allow incorporating preservation and sampling rates in the simulation and analysis of fossils in a phylogenetic context^14–18^, but the heterogeneity of preservation and recovery rates (across space, time, environments, and taxa) from extinct communities cannot be truly known. Therefore, our ability to correct for taphonomic, geologic, and anthropogenic biases in deep-time macroevolutionary studies is still limited.

The impact of biases in the fossil record has been mainly evaluated on estimates of richness and diversification (speciation and extinction) trends over geological time. Most works based on fossil occurrences highlight the distorting effect of spatial sampling biases over deeper timescales (i.e., hundreds of million years)^19–23^, though simulations suggest that the fossil record might preserve the signature of past biogeographic patterns^24^, especially over narrower periods (i.e., tens of million years)^25–29^. In a phylogenetic framework, fossil sampling has proven to impact tree reconstruction^30–36^ and time-calibration of diversification events^37,38^, with a general consensus that combining data from fossil and living taxa increases phylogenetic accuracy (as reviewed by ref. ^39–41^). Yet, the impact of fossil record biases on subsequent macroevolutionary inferences (particularly trait evolution) remains understudied^42–45^.

Here, we use data on extant taxa to explicitly simulate the effects of fossil record biases on macroevolutionary inferences. Starting from a community of living species, we generate a set of potential future fossil samples, attending to biases known to affect the fossil record, to then compare the evolutionary estimates that we derive from those partial samples with the real evolutionary estimates that we derive from the full set of living species. We use mammals as a working example, since they offer a suitable biological model: they are a speciose group, widespread over the planet, with a vast ecomorphological disparity, and one of the best studied^46^: there is considerable information available on their past (e.g., https://paleobiodb.org/#/^47^, https://nowdatabase.org/^48^) and present-day distributions (e.g., https://www.iucnredlist.org/, https://www.gbif.org/), traits, e.g., refs. ^49,50^, and phylogeny, e.g., refs. ^51,52^; and they have been the subject of previous investigations on fossil record biases, e.g., refs. ^25,29,36,53,54^.

**Fig. 1:**
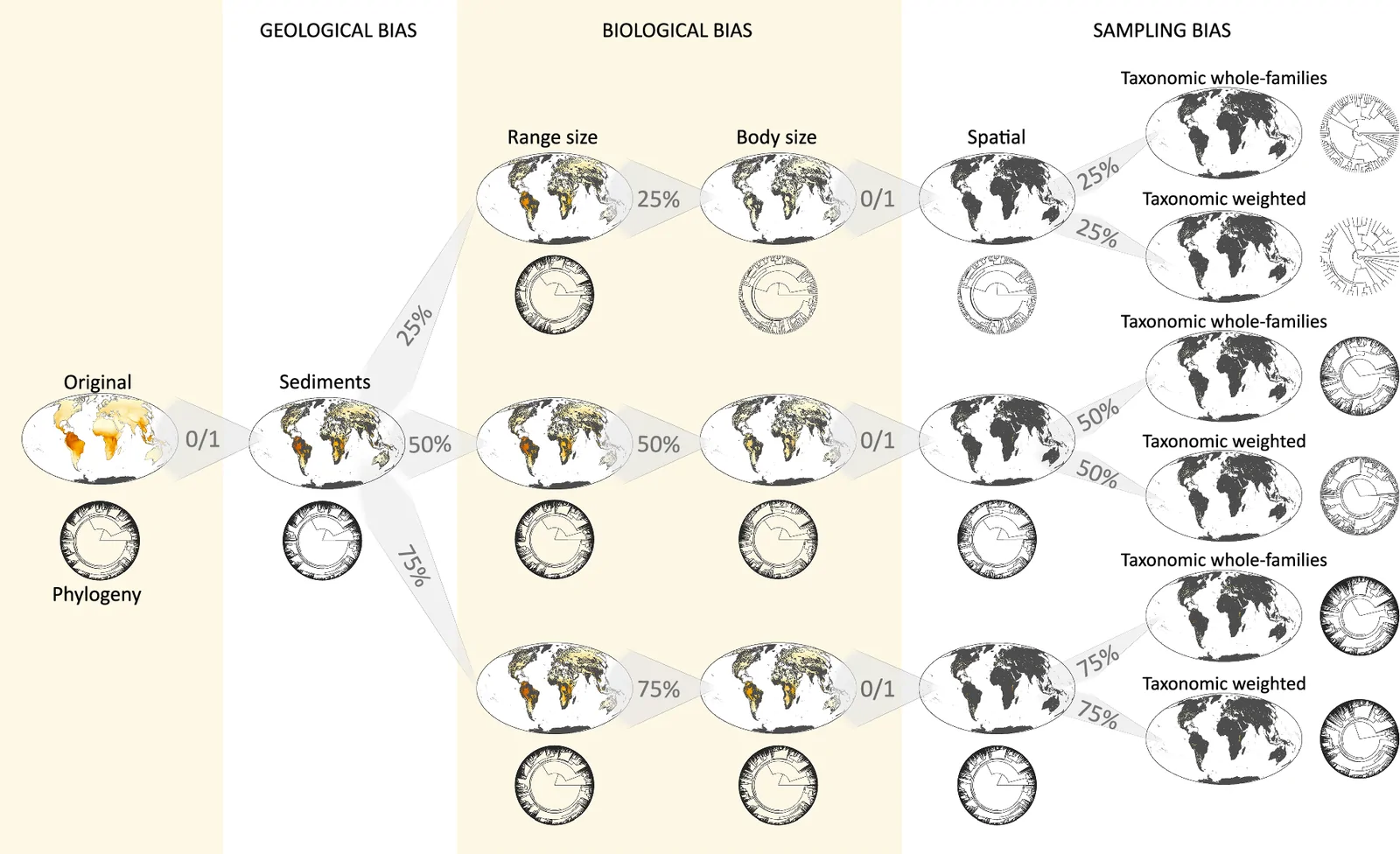
Consecutive simulation approach. From the current distribution (Original) and most recent time-calibrated tree (Phylogeny) of mammals, species are sequentially filtered out according to sediment availability (Sediments), species traits (Range size, Body size), and sampling biases (Spatial, Taxonomic—whole-families or weighted—) under three preservation rates (25%, 50%, 75%) respect to the previous step. After each step, filtered-out species are removed from the baseline mammal tree by pruning the corresponding branches. Phylogenetic analyses are performed on each pruned tree and results compared to the baseline. In the independent simulation approach, each filtering step is applied over the initial dataset (Original & Phylogeny). Adapted from ref. ^29^.

Specifically, we simulate which present-day land mammal species would become part of a future fossil record, according to geological (sediment availability), biological (species’ range and body size), and sampling-related (spatial and taxonomic) biases, under three different preservation scenarios. We conduct the analysis in a phylogenetic framework, taking the most recent phylogeny of mammals as our baseline from which we prune the species (tips) as they are filtered out through the process (Fig. 1). By doing so, we assume that the same backbone phylogeny would always be recovered, regardless of the number of species or data used for its reconstruction. We acknowledge this is rather unlikely^36,55^, but our aim here is to isolate the effect of fossil record biases on subsequent evolutionary estimates from the distortion caused primarily by inaccurate phylogenetic reconstruction. We also acknowledge that analysing samples from a single time period departs from common practice in the field, where fossils are generally distributed across multiple time periods throughout a phylogeny, but adding a temporal component to the simulation would imply even stronger assumptions on preservation and sampling processes (e.g., different land configurations or human settlements). In terms of inferences, we focus on the evolution of body mass and diet along the mammal phylogeny, two fundamental species’ traits^46,56–58^ that can be generally obtained from fossil remains^59–63^. We apply standard phylogenetic comparative methods to estimate how these traits are distributed across the phylogeny, their mode of evolution, and their ancestral states across simulated fossil samples with respect to baseline data.

Employing a forward simulation approach, we quantify the degree to which recognised biases in the fossil record might distort our understanding of past evolutionary trends, using land mammals as an example. We show that geographically driven biases have a lower impact than biologically and taxonomically driven biases, since the latter imbalance the structure of the community more than the former, preferentially removing specific parts of the mammal phylogeny and trait space from potential future fossils.

## Results

### Mammal sample composition

From our baseline phylogeny of 3710 mammal species, the number of retained species per filter decayed from 3294 after sediments to a minimum of 47 after taxonomy-weighted when filters were consecutively applied (consecutive approach) and to 910 after body-size when filters were independently applied (independent approach) under the lowest (25) preservation rate scenario (Supplementary Data 1). Overall, body-size affected the species composition and the proportion of trait categories in the samples the most, with a general increase in medium-sized and herbivorous species at the expense of smaller-sized and faunivorous species (Supplementary Figs. 2–4). The application of the body-size filter resulted in the exclusion of Chiroptera and Lipotyphla from part of the samples (13 and 26%, respectively), an effect that was enhanced by posterior taxonomic filters. The two orders were absent from all or nearly all samples after taxonomy-whole families (100 and 84%, respectively) and taxonomy-weighted (94 and 79%, respectively). In addition, Primates species were always excluded after taxonomy-whole families and in some cases after taxonomy-weighted (20%), and this last filter also resulted in the sporadic absence of Rodentia (3%). When applied independently, body-size caused the largest reduction of Chiroptera, Lipotyphla, and Rodentia species in the samples, while taxonomy-weighted did so for Primates and Carnivora, and range-size for Artiodactyla (Supplementary Data 2 and 3). On the other hand, the first spatial filter (sediments) had a negligible effect on the composition of the samples and subsequent inferences with respect to the baseline phylogeny. Similarly, the independent effect of the sampling spatial filter (spatial) was relatively low but moulded by previous filters in the consecutive approach.

Next, we describe results from the consecutive approach with specific references to results from the independent approach. Also, for discrete trait evolution, we focus on results under the all-rates-different (ARD) model of transition rates among character states, while results from the equal-rates (ER) model can be found in Supplementary Note 1. Finally, since maximum likelihood and Bayesian approaches for ancestral character reconstruction yielded similar results, we do not distinguish between them hereafter.

### Distribution of tip-diversification rates

The distribution of species-level diversification rate (DR) values became narrower as filters were consecutively applied (Supplementary Fig. 5). Median DR values consistently decreased across filters except for the last, the taxonomic ones, which tended to move estimates upwards (except for taxonomy-weighted in the 25 rate scenario). In this way, median DR values differed the most with respect to those from our baseline phylogeny after the spatial filter (Supplementary Data 4). The independent approach confirmed that the maximum decline in median DR values occurred after range-size and the minimum, after the taxonomic filters. Indeed, in the 75 rate scenario, the distribution of DR values after taxonomy-whole families did not differ from the baseline (Supplementary Data 4).

### Phylogenetic signal of continuous body mass

**Fig. 2:**
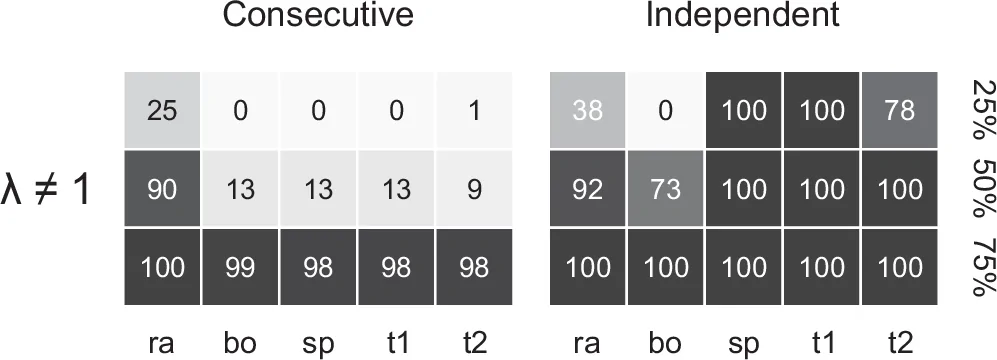
Phylogenetic signal of body mass as a continuous trait. The null hypothesis of *λ* = 1 is rejected (one-sided chi-square test, *p*-value = 9.78e-157) in the baseline phylogeny, as depicted in the margin. Heatmaps indicate the number of replicates, out of a hundred, that match the baseline result after each filtering step and preservation rate (25%, 50%, 75%) in the consecutive (left column) and independent (right column) approaches. Filter codes: ra range-size, bo body-size, sp spatial, t1 taxonomy-whole families, t2 taxonomy-weighted. The sediments filter is not depicted because of a consistent match with the baseline result.

The estimate of *λ* for body mass as a continuous trait was high in the baseline phylogeny (0.99) but still significantly different from 1 (*p*-value < 0.05; Supplementary Fig. 6). The largest variation in the estimates was observed after the taxonomic filters, especially taxonomy-weighted, in the 25 rate scenario. In general, the phylogenetic signal strengthened from the application of range-size onwards in this scenario and after body-size in the 50 rate one, supporting the null hypothesis of *λ* = 1 in most cases. The effect of body-size persisted when applied independently, followed by range-size and taxonomy-weighted under the lowest (25) preservation rate (Fig. 2 and Supplementary Fig. 6).

### Phylogenetic distribution of discrete body mass and diet

**Fig. 3:**
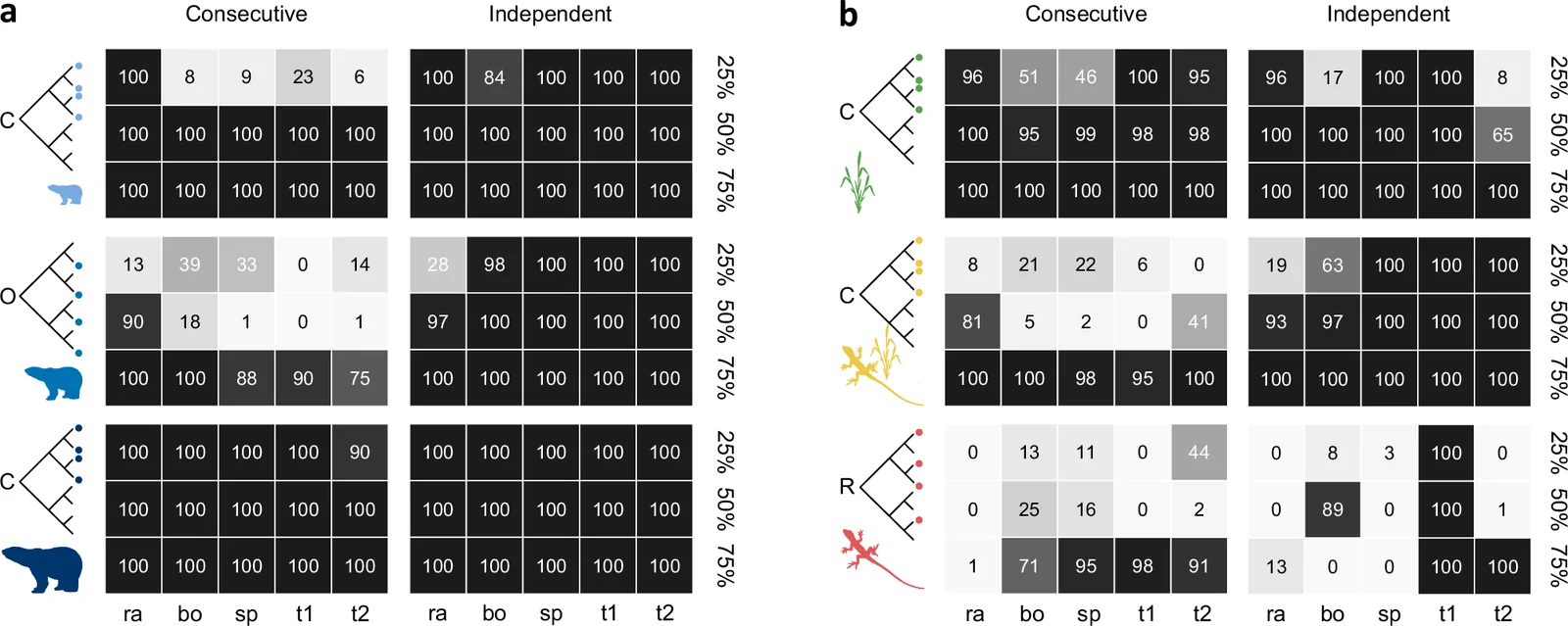
Phylogenetic distribution of trait categories. Patterns observed in the baseline phylogeny are indicated in the margins; C clustered, O overdispersed, R randomly distributed. From top to bottom, body mass categories (**a**) are smaller- (0–500 g, light blue), medium- (500.1–50,000 g, blue), and larger-sizes (> 50,000 g, dark blue); and diet categories (**b**) are herbivore (green), omnivore (yellow), and faunivore (red). Heatmaps indicate the number of replicates, out of a hundred, that match the baseline pattern after each filtering step and preservation rate (25%, 50%, 75%) in the consecutive (left column) and independent (right column) approaches. Filter codes: ra range-size, bo body-size, sp spatial, t1 taxonomy-whole families, t2 taxonomy-weighted. The sediments filter is not depicted because of a consistent match with the baseline result. Species silhouettes were downloaded from PhyloPic (https://www.phylopic.org): *Ursus maritimus* by Tracy Heath is reprinted under a CC0 1.0 Universal Public Domain Dedication license (**a**); *Triticum aestivum aestivum* by Mason McNair and *Anolis carolinensis* by Christoph Schomburg are reprinted under a CC0 1.0 Universal Public Domain Dedication license (**b**).

According to net relatedness index (NRI) analyses, smaller- (0–500 g) and larger-sized (> 50,000 g) species were significantly clustered in the baseline phylogeny, while medium-sized (500.1–50,000 g) ones were overdispersed (Fig. 3a and Supplementary Fig. 7). For larger species, the pattern was basically maintained. For smaller species, it was only distorted under the lowest (25) preservation rate scenario, mostly showing a random distribution after body-size and subsequent filters (> 75% replicates per filter) and even overdispersion in a few cases (≤ 10% replicates per filter). On the other hand, the overdispersion of medium-sized species was not recovered in general. Their distribution was rather random after range-size in the 25 rate scenario and after body-size in the 50 rate scenario, turning into clustering after taxonomic filters (~20% replicates per filter) in both scenarios. The independent approach supported a major impact of range-size on the pattern of medium-sized species and only a minor effect of body-size on the pattern of smaller-sized ones (Fig. 3a and Supplementary Fig. 7).

Regarding diet, herbivorous and omnivorous species were clustered in the baseline phylogeny while faunivorous species were randomly distributed (Fig. 3b and Supplementary Fig. 8). The pattern of herbivores was the least altered, changing to random in nearly half of the samples after body-size and spatial filters in the 25 rate scenario. The clustering of omnivores was largely rejected in the 25 and 50 rate scenarios. In the first, the pattern eventually shifted to overdispersion after taxonomic filters (5% after taxonomy-whole families and 12% after taxonomy-weighted). Results for faunivores were the most distorted across filters and rates. The baseline random pattern changed to clustering in most cases in the 25 and 50 rate scenarios, as well as after range-size in the 75 rate scenario. In the latter scenario, it was even reversed to overdispersion mainly after body-size (29%) but also after subsequent filters. Based on the independent approach, the distribution pattern of herbivores was most impacted by taxonomy-weighted and that of omnivores by range-size, both followed by body-size. In the case of faunivores, all filters but taxonomy-whole families had a notable effect. Range-size and spatial filters consistently rendered a pattern of clustering, whereas taxonomy-weighted led to overdispersion except for the 75 rate scenario. On the other hand, body-size caused clustering in the lowest (25) preservation rate scenario but overdispersion in the highest (75) (Supplementary Fig. 8).

### Evolution of body mass as a continuous trait

**Fig. 4:**
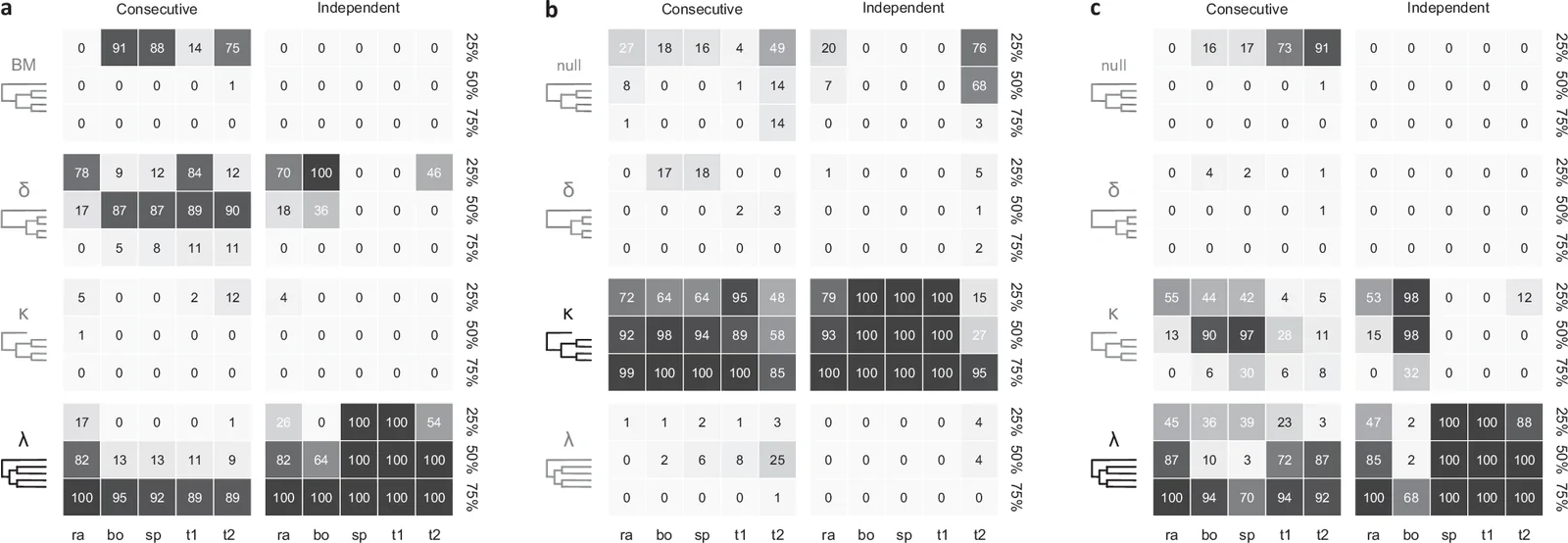
Model support for the evolution of traits. Evolution of body mass as a continuous (log10 transformed, **a**) and discrete (three categories, **b**) trait, and of diet as a discrete trait (three categories, **c**). Heatmaps indicate the number of replicates, out of a hundred, where the best supported model was that shown in the margin. BM Brownian-Motion, null no tree transformation, δ delta (time-dependent), κ kappa (speciational), λ lambda (covariation) models. Results are depicted per filtering step and preservation rate (25%, 50%, 75%) in the consecutive (left column) and independent (right column) approaches. For discrete traits, results correspond to all-rates-different (ARD) transition model. In the left margin, the inference from the baseline phylogeny is highlighted in black. Filter codes: ra range-size, bo body-size, sp spatial, t1 taxonomy-whole families, t2 taxonomy-weighted. The sediments filter is not depicted because of a consistent match with the baseline result.

Body mass evolved according to a lambda (covariance among trait values for species) model in the baseline phylogeny (Akaike weight = 1, *λ* = 0.99) when it was analysed as a continuous trait (Fig. 4a, Supplementary Fig. 9a and Supplementary Data 5). This result, however, was not supported in most cases in the 25 and 50 rate scenarios. In the first, a delta (time-dependent evolution) model was preferentially selected after range-size and taxonomy-whole families filters (Akaike weight ≥ 0.57, *δ* < 1) and the Brownian motion (BM) model after the others (Akaike weight ≥ 0.41). In the second, delta remained as the best fitted model from body-size onwards (Akaike weight ≥ 0.74, *δ* < 1). The independent approach highlighted a major role of body-size in distorting the baseline inference, followed by range-size and taxonomy-weighted (Fig. 4a and Supplementary Fig. 9a).

In general, the estimated body mass of the mammal ancestor progressively increased with respect to the baseline value (~790 g) across filters, with more variance after the taxonomic ones (Supplementary Fig. 10a). This pattern was consistent across mammal orders (Supplementary Fig. 10b–g) except for Chiroptera and Lipotyphla, which showed smaller ancestral sizes mostly after the taxonomic filters in the 25 rate scenario. Estimates for ancestral Lipotyphla, though, remained below the baseline even in the 75 rate scenario. The independent approach reinforced the overestimating effect of the body-size filter on ancestral sizes and the variable effect of the taxonomic filters depending on the mammal group. Meanwhile, range-size occasionally rendered underestimated ancestral sizes, particularly for Lipotyphla and Artiodactyla (Supplementary Fig. 10b–g).

### Evolution of body mass as a discrete trait

When body mass was analysed as a discrete trait with three categories (i.e., smaller- [0–500 g], medium- [500.1–50,000 g], and larger-size [> 50,000 g]), it was best fit by a kappa (punctuated/speciational evolution) model in the baseline phylogeny (Akaike weight = 0.98, *κ* = 0.68) (Fig. 4b, Supplementary Fig. 9b and Supplementary Data 6). This result was maintained across all filter and rate combinations despite support fluctuations over samples, being lowest after taxonomy-weighted (Akaike weight ≤ 0.53). The independent approach highlighted the largest impact of this filter on model selection, favouring the null model (no tree transformation) (Akaike weight ≥ 0.39) over the baseline kappa (Akaike weight ≤ 0.30) under all preservation rates but the highest (75). It was followed by range-size, which lowered the support of the baseline model too (Fig. 4b and Supplementary Fig. 9b).

**Fig. 5:**
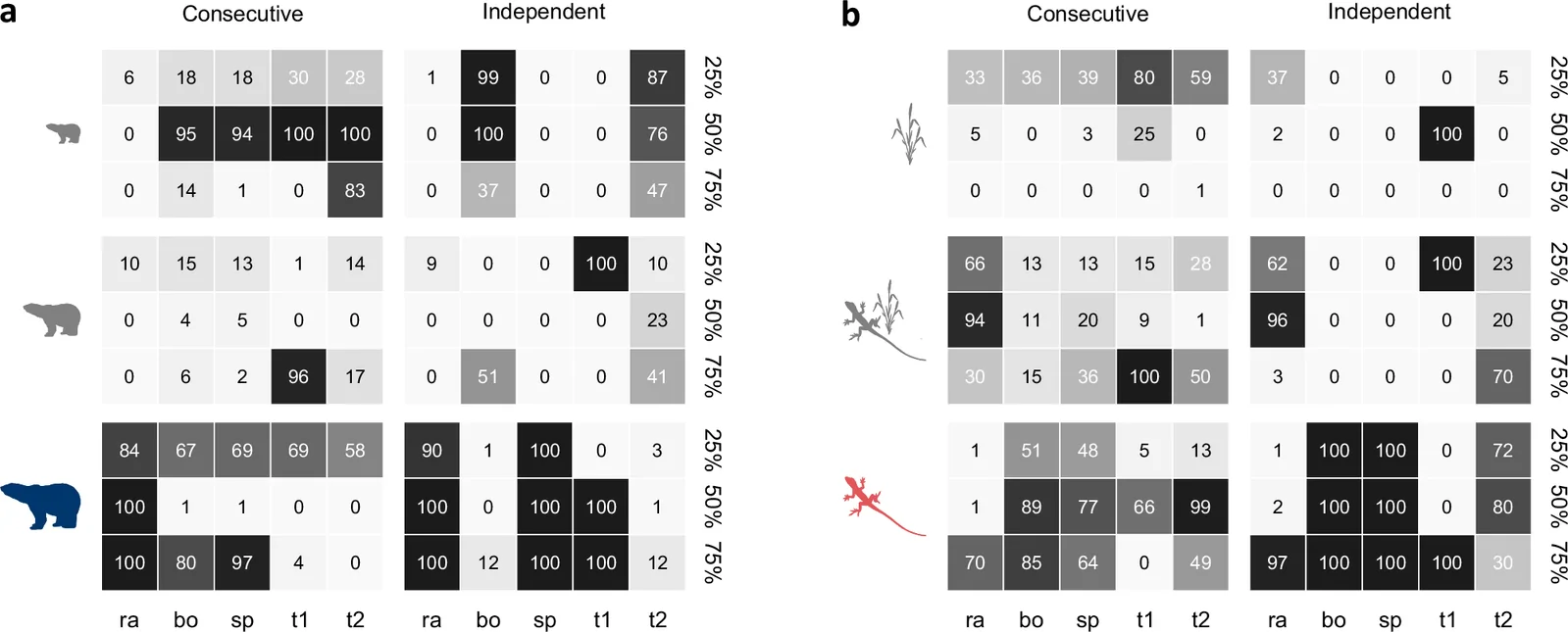
Maximum likelihood ancestral character reconstruction of discrete traits. Reconstruction of body mass (**a**) and diet (**b**) under all-rates-different (ARD) transition model, for the mammal ancestor. Heatmaps indicate the number of replicates, out of a hundred, where the most likely ancestral state was that shown in the margin. For body mass (**a**), categories are smaller- (0–500 g), medium- (500.1–50,000 g), and larger-size (> 50,000 g) ancestor (from top to bottom). For diet (**b**), categories are herbivore, omnivore, and faunivore ancestor (from top to bottom). Results are depicted per filtering step and preservation rate (25%, 50%, 75%) in the consecutive (left column) and independent (right column) approaches. In the left margin, the inference from the baseline phylogeny is highlighted in colour (in contrast to the general grayscale). Filter codes: ra range-size, bo body-size, sp spatial, t1 taxonomy-whole families, t2 taxonomy-weighted. The sediments filter is not depicted because of a consistent match with the baseline result. Species silhouettes were downloaded from PhyloPic (https://www.phylopic.org): *Ursus maritimus* by Tracy Heath is reprinted under a CC0 1.0 Universal Public Domain Dedication license (**a**); *Triticum aestivum aestivum* by Mason McNair and *Anolis carolinensis* by Christoph Schomburg are reprinted under a CC0 1.0 Universal Public Domain Dedication license (**b**).

The mammal ancestor most likely belonged to the larger-size category, although the probability of the medium-size category was relatively close to it (mean probability across replicates = 0.40, vs. 0.48) (Fig. 5a and Supplementary Figs. 11 and 12). Once the body-size filter was applied, a trend towards smaller categories was observed primarily in the 50 rate scenario. The independent approach showed the largest impact of taxonomy-weighted on ancestral estimates, an impact that persisted across preservation rate scenarios. Rodentia and Primates presented a similar pattern, but starting from a medium-sized ancestor in the baseline phylogeny. In this case, the effect of body-size was the highest, with taxonomy-whole families and taxonomy-weighted as next, respectively. Baseline estimates for Lipotyphla (medium-sized ancestor) were consistently distorted in all rate scenarios, mostly by both taxonomic filters, but also by body-size and range-size. On the other hand, inferences for Carnivora (medium-sized ancestor) and especially for Artiodactyla (larger-sized ancestor) were barely affected overall (Supplementary Figs. 11 and 12).

### Evolution of diet as a discrete trait

The evolution of diet (i.e., herbivore, omnivore, and faunivore) along the baseline phylogeny best fitted a lambda model (Akaike weight = 1, *λ* = 0.97) (Fig. 4c, Supplementary Fig. 9c and Supplementary Data 7), but this was not supported from range-size onwards in the 25 rate scenario (Akaike weight ≤ 0.45), neither after body-size and spatial in the 50 rate scenario (Akaike weight ≤ 0.11). The independent approach showed a major effect of body-size on model selection, which persisted across preservation rate scenarios, while the effect of range-size was also important but limited to the lowest (25) rate scenario (Fig. 4c and Supplementary Fig. 9c).

The mammal ancestor was most likely a faunivore (mean probability across replicates = 0.48) according to the baseline phylogeny, but this inference also changed after range-size and subsequent filters in the 25 rate scenario (Fig. 5b and Supplementary Figs. 13 and 14). Omnivory was rather supported after range-size and herbivory after the others, more clearly after taxonomy-whole families. The effect of range-size was still detected in the 50 rate scenario and when applied independently. The independent approach also highlighted the impact of taxonomy-whole families on the estimates (Fig. 5b). The pattern of state probabilities varied across orders. Omnivory was inferred as the most likely ancestral state for Rodentia, Primates, and Carnivora, while faunivory was so for Chiroptera and Lipotyphla, and omnivory and herbivory were similarly supported for Artiodactyla. Relative baseline probabilities were altered from the application of range-size onwards for Rodentia, Chiroptera and Lipotyphla, an effect remarked by the independent approach. The taxonomic filters caused the shift for Artiodactyla and Carnivora in the 25 and 50 rate scenarios, respectively, while the independent effect of body-size was the highest for the latter order. Finally, the most likely ancestral state for Primates did not change despite probability fluctuations across filters (Supplementary Figs. 13 and 14).

## Discussion

Our study illustrates how the incompleteness and non-uniformity of the fossil record across space and taxa can affect inferences on lineage evolution regarding two fundamental traits, body mass and diet. We used present-day land mammal species as a baseline to simulate future fossils and to compare the information we could retrieve from those partial samples with respect to that obtained from the complete set of species in a phylogenetic context, by means of commonly applied phylogenetic comparative methods. Among the sources of bias in the fossil record that we have implemented here (geological, biological, and sampling related), our results show that biological (body-size and range-size) and taxonomic (taxonomy-whole families and taxonomy-weighted) filters may significantly impact the inferences, especially under the most stringent filtering conditions (25 preservation rate scenario), although their relative importance depends on the trait, method, and mammal group considered (Figs. 2–5 and Supplementary Figs. 10–14). In contrast, the impact of spatial filters (sediments and spatial sampling effort) appears largely negligible. Overall, the impact of each filter can be explained by the interaction between the number of species retained, their position along the phylogeny, and their coverage of the baseline trait diversity. As expected, the fewer the species and the more unequal representation of mammal clades and trait space in the simulated fossil sample, the higher the distortion on derived phylogenetic estimates^45,64–66^.

On average, in the sequential approach and under the lowest (25) preservation rate, the simulated fossil samples only contain from 1.3% (after taxonomy-weighted filter) to 3.0% (after taxonomy-whole families filter) of the initial 3710 species, with a remarkable underrepresentation of major mammal orders (Supplementary Data 1–3). In fact, chiropterans and primates would leave no trace at all in a future fossil record in the latter case and nearly so in the first. Similarly, lipotyphlans would be missing from most samples and yet rodents would eventually be lost. The four orders together account for 76% of the species in the initial dataset, and rodents alone account for 39% of the total. These results indicate a strong bias in the fossil record in absolute and relative terms. They align with previous estimates on Miocene mammal fossils pointing to approximately 4% completeness of the record, but much lower for small mammals and primates^67^. Thus, our preservation and sampling protocol seems to reasonably resemble what is observed in the fossil record. In higher preservation rate scenarios, the imbalance across groups persists, although all major orders are retained. The total coverage increases to 9.9 and 15.6% under the 50 preservation rate, also approaching the 16% inferred for Pleistocene mammals^53^, and to 31.1 and 38.8% under the highest (75) preservation rate.

The main distortion in simulated fossil samples with respect to the present-day mammal biodiversity is driven by the body-size filter (Supplementary Figs. 2–4), as reported in other studies^28^. This is not surprising since body mass is an essential axis of mammal diversity and most living mammals are small^46^. The application of the body-size filter causes an overrepresentation of medium-sized and herbivorous species at the expense of smaller-sized and faunivores, further altering the species composition within trait categories. Smaller species are mostly chiropterans, lipotyphlans, and rodents, with the first two orders accounting for most faunivores, and the latter for omnivores and herbivores. Moreover, among placental mammals, these groups belong to different clades: chiropterans and lipotyphlans (together with carnivorans and artiodactyls) on one hand and rodents (together with primates) on the other^51,52^. Therefore, as smaller species are filtered out, with carnivorans and artiodactyls becoming predominant groups, the structure of the samples necessarily changes, regarding both mammal phylogeny and trait space. As noted above, the disrupting effect is more evident when fewer species are retained and generally enhanced by posterior taxonomic filters. The taxonomic biases that we have simulated preferentially retain closely related species. In the pruned trees, this translates into the retention of relatively more nodes, denser branching patterns (i.e., fewer long branches), which render higher DR values^68^ over simulated fossil samples compared to other filters. It particularly occurs after the taxonomy-whole families filter, when the most frequent families are kept in full. We observe the opposite effect (overall lower rates) when small-ranged mammals are preferentially lost (range-size filter; Supplementary Figs. 1 and 5), in line with the proposed inverse relationship between range size and DR (but see ref. ^69^).

In terms of traits, body mass and diet show a strong phylogenetic signal in mammals that would be mostly disrupted in potential future fossil samples, attending to the distribution of trait categories along the phylogeny (Figs. 2 and 3). Larger mammals (mainly artiodactyls, robust to the filtering process) would preserve a signature of clustering. On the other hand, the number of smaller species, while consistently represented by rodents, would decrease to the point that there would be no power to detect the baseline aggregation under the lowest (25) preservation rate scenario. Medium-sized mammals, initially spread among groups, would lose the signature of overdispersion, eventually turning into clustering, in most fossil samples from the application of the range-size filter onwards (except under the highest [75] preservation rate). The filtering process reveals a higher impact on diet than on body mass, despite a more even number of species per diet category than per body mass category (Supplementary Figs. 3 and 4). The exception corresponds to herbivores, where the baseline signal of aggregation would remain regardless of the progressive replacement of rodents by artiodactyls. Within omnivores, baseline clustering would turn into stochasticity, as carnivorans and artiodactyls take over rodents. Meanwhile, the random pattern of faunivores would mainly change to clustering because of the increased presence of carnivorans over chiropterans and lipotyphlans, even under the highest (75) preservation rate scenario.

The phylogenetic signal of body mass is further reflected in the support of the covariation model of evolution (lambda) when it is analysed as a continuous trait (Fig. 4a and Supplementary Data 5). Nonetheless, in agreement with the distortion of the mammal community, this signal would be erased in most fossil samples under both 25 and 50 preservation rate scenarios. Samples would point either to stochastic evolution (Brownian motion) or to deceleration of evolution towards the present ("early burst" [EB] model, *δ* < 1). When body mass is categorised, the variation and the information on the trait diminish. In this way, it adjusts to the speciational model of evolution (kappa) irrespective of the transition matrix considered, and this estimate would generally stand in future fossil samples (Fig. 4b, Supplementary Fig. 15a and Supplementary Data 6). For diet, the best model of evolution depends on the transition matrix used instead. It would mainly move between lambda and kappa models across filters and samples, with the null model also suggested under the lowest (25) preservation rate scenario (Fig. 4c, Supplementary Fig. 15b and Supplementary Data 7). Overall, our results support that potential future fossils would render disparate inferences about the mode of evolution of these fundamental traits along the diversification of mammals compared to present-day species.

The ancestral reconstruction of body mass as a continuous trait highlights the overestimating effect of the body-size filter and the variable effects of the two taxonomic filters (Supplementary Fig. 10). For land mammals altogether, fossil samples could suggest an ancestor one order of magnitude bigger (> 10 kg) than inferred from present-day species (~790 g) under most stringent filtering. A similar pattern would apply to major orders, except for chiropterans and lipotyphlans, which show more variable results after biological and taxonomic filters. When body mass is categorised, baseline data point to a larger or smaller ancestor depending on the transition matrix used (ARD and ER, respectively), but with the probability of a medium-sized ancestor always close to the highest (Fig. 5a and Supplementary Fig. 16a), reflecting the expected uncertainty on these estimates (see ref. ^70^ and references therein). Regardless of the model, the baseline results would not be recovered from most fossil samples once the body-size filter is applied. For instance, when different transition rates are allowed between each pair of trait categories (ARD), a trend towards the smaller-sized state becomes more evident in the 50 rate scenario. This may seem counter-intuitive but reflects the interplay between category frequencies and the estimated transition matrix in each sample. Assuming a single transition rate (ER), inferences move towards a medium-sized state instead. In any case, inferences from fossil samples would tend to suggest opposite patterns of body mass evolution in mammals compared to baseline inferences. The uncertainty on the diet of the mammal ancestor is even higher, but faunivory is supported as the most likely state (Fig. 5b and Supplementary Fig. 16b). As for body mass, this baseline result would be rarely obtained from future fossils. Either an omnivore or herbivore ancestor would be suggested (under ARD and ER, respectively). Among major orders, the least altered inferences would correspond to artiodactyls and carnivorans, since in general they would be less affected by the filtering process. All in all, the different estimates on trait evolution and ancestral states from potential future fossil samples would have profound implications on the understanding of the factors and processes underlying the diversification of mammals in the context of the environmental conditions they experienced over time.

Regarding the low impact of spatial biases on our sampling simulation, we remark that the sediments filter only accounts for the impossibility of fossilisation when appropriate sediments are absent from the area occupied by a species. While the presence of sediments is considered essential for fossilisation, it alone is not sufficient for fossilisation to occur, as distinct diagenetic processes are required to geologically stabilise the remains^71^. Furthermore, these processes (i.e., taphonomic filters) can also impact fossil accessibility and identification upon collection over time^29,54^. The different preservation rate scenarios we implemented aim to cover those processes, while sediments work as a first soft filter. It retains 88.8% of the species in our baseline dataset (Supplementary Data 1), in line with the 81.3% previously reported for terrestrial mammals based on long-term sedimentary areas^54^. Importantly, the species are filtered out in a similar way across major mammal orders (Supplementary Data 2 and 3), without causing a notable imbalance on the pruned mammal tree compared to the baseline and so on subsequent phylogenetic inferences^65,66^. The sampling spatial filter has an analogous effect. When applied independently, it maintains 85.7% of the species, fairly distributed across orders. In the sequential approach, its effects are necessarily moulded by the previous filters but do not stand out in general. These results contrast with the demonstrated role of spatial sampling biases on obscuring deep-time biodiversity patterns over different geological periods with heterogeneous fossil records^21,72–75^. That temporal factor was out of the scope of this study, but the lack of it in our simulation could explain such discrepancy.

While the rationale and limitations of our fossil sampling simulation have been discussed when first described in ref. ^29^ and briefly above, we want to remark the caveats of our phylogenetic comparative analysis. We have assumed that the inferred relationships between mammal species will remain robust to the sample size and the type of data used for reconstruction, that the same backbone phylogeny will always be recovered, either from a set of 3710 species (with genomic data) or from a minimum sample of 47 (with morphological data), which is rather unlikely^36,55^ but also a conservative approach to the effects of fossil record biases on derived estimates. The variance on the inferred phylogeny across replicates would propagate to all subsequent analyses, most probably increasing the disparity between replicates as well as with respect to the baseline. Likewise, our results are bound to the assumptions of the baseline phylogeny^52^. Including the uncertainty on topology and node ages (and hence on branch lengths) will imply working with multiple trees and high posterior density intervals instead of with the most plausible estimates, expanding the parameter space to explore and thus computational needs. Nonetheless, some common comparative methods as those we applied here (e.g., phylogenetic signal) appear to be robust to tree misspecification, at least in terms of general trends^76,77^. For simplicity, we have also compared the results from simulated fossil samples with respect to the baseline, attending to the most supported estimates per replicate (i.e., percentage of samples matching the baseline pattern) and across replicates (i.e., mean parameter values) instead of focussing on uncertainty around individual estimates, which may be preferred in a phylogenetic context^41,78^. Still, the replicates of the simulation process offer some measure of that uncertainty. In addition, the baseline phylogeny is an ultrametric (extant-only) tree from which we have removed species by pruning the corresponding tips, so our simulated fossil samples represent a single point in time. This contrasts with most studies on fossil remains, where samples of different ages (fossil time-series) are generally analysed, helping trace evolutionary paths^79–82^. We acknowledge that our approach is closer to studies on extant taxa dealing with the effect of incomplete sampling on phylogenetically derived estimates^64–66,83,84^, and indeed arrives at the same conclusion: that non-random sampling causes the most skewed estimates. Yet, our incomplete sampling is non-random in a specific way: it reflects biases known to affect the fossil record and further accounts for different preservation scenarios. Therefore, though certainly simplistic, our approach gives some insights into how fossil record biases might distort our interpretation of extinct communities and their trajectories.

Finally, state-of-the-art methods allow the integration of data on extinct and extant organisms^33,36,85,86^, increasing the power to explore and elucidate deep-time diversification patterns (as reviewed by refs. ^39–41^), while our simulation would approximate those cases where other sources of information on lineage evolution are rather sparse beyond a relatively time-limited fossil record. Besides, it is rooted in empirical data instead of in purely theoretical simulations, where the true underlying parameters are known. In this respect, simulation tools accounting for diverse biases in the fossil record (across space, time, and taxa) have become available, such as FossilSim^16^, DeepDive^17,18^ or simulations based on ecological neutral theory^22^, that allow evaluating alternative hypotheses on biodiversity change through time. Nevertheless, these approaches can complement each other. Empirically driven works can help guide the selection of parameters or scenarios to test, while theoretical models can inform on the performance and likelihood of such scenarios.

In conclusion, taking land mammals as an example, our study illustrates how the incomplete and biased nature of the fossil record may impact our inferences on the evolutionary pathways of life on Earth. While no one doubts the value of the fossil record, it may fail to capture lineage-specific processes and trends, providing biased reconstructions when read literally. Even for groups with a relatively high chance of fossilisation (as mammals), fossils may render a distorted picture of their past biodiversity and trait evolution.

## Methods

### Mammal data: distribution, phylogeny, and traits

We downloaded distribution maps for terrestrial and freshwater (land) mammals from the IUCN^87^ on 24th January and 21st September 2022, respectively. We loaded range shapefiles in R (v.4.2.0)^88^ using the rgdal package (v.1.6-6)^89^. We excluded polygons (i.e., map areas) associated with families of predominantly aquatic/marine habits, namely dolphins (Delphinidae, Iniidae, Lipotidae, Platanistidae) and porpoises (Phocoenidae), manatees (Trichechidae), and true seals (Phocidae). We also filtered out polygons where the species presence was uncertain (presence codes 3, 6) and that did not correspond to the species native range (origin codes 3, 4), resulting in a set of 5739 species. We rasterised each species range at a spatial resolution of 0.5° latitude and 0.5° longitude using the terra package (v.1.6-7)^90^.

We used the most recent time-calibrated phylogeny of mammals^52^, available at https://github.com/sabifo4/mammals_dating/blob/main/02_SeqBayes_S2/03_Generate_final_mammal_tree/4705sp.tree, as our baseline for analyses. This is a consensus phylogeny inferred from genome-wide data under a sequential Bayesian approach that comprises 4705 extant mammal taxa. Before analysis, we conducted taxonomic harmonisation between IUCN and the phylogeny. In all 257 conflicts, we adopted the nomenclatural usage and systematic treatment applied by ref. ^52^, updating IUCN names accordingly (Supplementary Data 8). Specifically, for 19 genus-level tips, we assigned the respective type species; for 95 subspecies-level tips, we collapsed terminals to species rank, replacing the infraspecific epithet with the accepted species name; and we further adapted the name of 143 tip species. In this way, our working dataset consisted of 3710 mammal species with information on both distribution and phylogenetic position.

We retrieved trait data for mammals from the COMBINE database^50^. Specifically, we extracted body mass (adult_mass_g) and diet (trophic_level), together with order assignment, for those species with matching nomenclature between IUCN distribution maps and COMBINE (iucn2020_binomial field). We then discarded species absent from the phylogeny, resulting in 3641 and 3660 species with data on body mass and diet, respectively, in our working dataset.

### Fossil sampling simulations

We sampled mammal species while considering biases known to affect the fossil record, from geological, biological, and anthropogenic sources, as in ref. ^29^. Briefly, geological bias here refers to the fact that fossilisation can only occur if appropriate sediments are available. To simulate this, we downloaded a global map of unconsolidated sediments currently exposed on the land surface^91^, consisting of 39 sedimentary classes. Among them, we excluded “ice and glaciers”, “proglacial”, “till”, and “glacial, undifferentiated” because of their extremely low preservation potential, while all water bodies were retained. We assigned a presence/absence code (1/0) to each map cell and filtered out those cells coded as 0 (sediments filter hereafter). Biological biases account for the lower probability of fossilisation expected for species with smaller geographic range and body size, implemented as species sampling weights in our approach (range-size and body-size filters hereafter). Anthropogenic biases describe the unequal coverage of geographic regions and taxonomic groups in the fossil record due to research preferences and possibilities. To define these, we extracted all fossil records for mammals, from all geological periods, from the Paleobiology Database (PBDB; https://paleobiodb.org/#/) on 12th January 2023. Based on geographic coordinates of fossil occurrences, we coded map cells as containing or not (1/0) fossil sites and filtered out the latter (spatial filter hereafter). To represent the taxonomic bias, we calculated the number of observations per mammal family in the PBDB and followed two alternative sampling strategies: we either (1) retained all species from the families with the highest number of observations (taxonomy-whole families filter hereafter) or we (2) sampled species weighting by the number of observations of the family they belong to (taxonomy-weighted filter hereafter). We used the geological, biological, and anthropogenic filters both consecutively and independently, aiming to assess their cumulative and individual effects but acknowledging that some interaction among filters cannot be eliminated^29^. For instance, range size and body size are generally correlated in mammals^46^, as well as the frequency of a given family in PBDB is not only the result of human research interests or capacity but also of the conditions that made possible their preservation (sediment availability, species’ range size, body size) and discovery (sampling spatial effort). In the consecutive approach, the order of the filters was: sediments, range-size, body-size, and spatial followed by either taxonomy-whole families or taxonomy-weighted (Fig. 1). In the independent approach, each filter was implemented upon the initial working dataset (phylogeny filter hereafter). In any case, whenever sampling was involved (i.e., for non-binary filters), we applied three levels of species recovery with respect to the previous step in the simulation: 25, 50 and 75% (25, 50 and 75 preservation rates hereafter) (Fig. 1). We generated 100 sampling processes (i.e., replicates) under each approach, with each replicate starting from a different random seed and rendering a list of retained species after each filter and preservation rate combination (i.e., a total of 4200 samples: 2 approaches × 3 rates × 7 filters × 100 replicates).

### Phylogenetic comparative methods

Our aim was to evaluate the impact of incomplete and biased sampling, as expected from the fossil record, on evolutionary inferences in a phylogenetic context. We focused on tip-diversification rates and trait evolution (body mass and diet) along the mammal tree. We conducted the analyses on the trees obtained after every filtering step per replicate and approach (Supplementary Fig. 1). We obtained these trees by pruning the discarded species in the corresponding step from the baseline phylogeny of 3710 species, with the drop.tip function from the ape package (v.5.8)^92^.

To estimate tip-diversification rates, we used the species-level DR metric^68^, which depends on the number of splitting events and internode distances along the path from the root of the tree to the tip, giving more weight to branches closer to the present. High DR values denote frequent branching events towards the present, while low DR values point to long (deep) branches not shared among species^51^. Although this metric was originally described as a measure of net diversification (i.e., speciation minus extinction), it is a better estimate of the rate of speciation^93^. We calculated DR using the evol.distinct function from the picante package (v.1.8.2)^94^ under the equal splits method with untransformed branch lengths, and compared DR distributions between trees. Since branch lengths are indicated in 100 million years (Myr) in the baseline phylogeny^52^, we multiplied them by 100 beforehand to obtain species per one Myr as the unit of diversification.

For body mass analysis, we transformed the values to log10 scale because of the wide and skewed distribution of this trait in mammals^46^. We also defined three body mass categories, trying to balance the number of observations and the range of values (in orders of magnitude) per category, and to approximate small (0–500 g), medium (500.1–50,000 g), and large mammal species (> 50,000 g). This allowed us to assess the effect of treating body mass as either a continuous or a discrete trait. For diet, we used the three main trophic levels, herbivore, omnivore, and carnivore (faunivore hereafter), as retrieved from the COMBINE database.

We first evaluated the phylogenetic signal of the traits, defined as the tendency for closely related species to resemble each other more than less related ones because of shared evolutionary history^95^. In the case of body mass as a continuous trait, we used Pagel’s *λ*^96^ as implemented in the phylosig function from the phytools package (v.1.5-1)^97^. Pagel’s *λ* ranges from 0, indicating no phylogenetic signal, to 1, indicating that the trait evolved on the phylogeny as expected under the Brownian motion model^96^. We tested whether observed *λ* values differed from 1 (*λ* = 1 as our null hypothesis). In the case of discrete traits, we evaluated how they were distributed along the phylogeny, whether clustered or overdispersed. To do so, we grouped the species by trait category, treated each group as a community, and applied the net relatedness index (NRI)^98^. NRI is based on the mean phylogenetic distances between co-occurring taxa in a community, with positive NRI values representing phylogenetic clustering and negative NRI values, overdispersion with respect to a random distribution. We calculated NRI using the ses.mpd function from the picante package.

To assess the mode of trait evolution along the phylogeny, we fitted likelihood models for both continuous and discrete traits using the fitContinuous and fitDiscrete functions (respectively), and compared them by means of the Akaike information criterion using the aicw function, all from the geiger package (v.2.0.11)^99^. For continuous traits, we compared Brownian motion (BM)^100^ and Pagel’s tree transformation models^96^: lambda (*λ*, covariance among trait values for species), kappa (*κ*, punctuated/speciational evolution), and delta (*δ*, time-dependent evolution). Both *λ* and *κ* range from 0 to 1, while *δ* can take any value. When any of the parameters is 1, the model equates to BM. A *λ* of 0 means that the trait has evolved independently of the phylogeny, whereas a *κ* of 0 indicates that changes only occurred at branching (speciation) events. *δ* values below or over 1 point to slowdown or acceleration of evolution towards the present, here largely corresponding to “early burst” (EB)^101^ and “Ornstein Uhlenbeck” (OU)^102^ models of trait evolution, respectively. For discrete traits, we evaluated models under both equal and all different transition rates between character states (ER and ARD, respectively), either specifying or not (null model) one of the three Pagel’s tree transformations.

We further reconstructed character states at the root of the tree and at nodes corresponding to the ancestors of mammal orders with more than 200 species in the baseline phylogeny: Rodentia, Chiroptera, Primates, Lipotyphla, Carnivora, and Artiodactyla. For continuous traits (log10-scaled body mass), we used the maximum likelihood approach implemented in the fastAnc function from phytools. For discrete traits (categorical body mass and diet), we used maximum likelihood and Bayesian approaches under both ER and ARD transition rate models. We obtained maximum likelihood estimates with the function ace from ape and Bayesian estimates with the function make.simmap from phytools. For the latter, we set 100 simulations per input tree, an equal prior distribution of character states at the root, and an empirical transition matrix (i.e., the rate matrix is first fitted under a continuous-time reversible Markov model and then used, together with tip states, for stochastic character mapping along the tree).

### Reporting summary

Further information on research design is available in the Nature Portfolio Reporting Summary linked to this article.

## Supplementary information

Supplementary Information

Description of Additional Supplementary Files

Suplementary Data 1 to 8

Reporting Summary

Transparent Peer Review file

## Data Availability

The data used in this study are available in the Supporting Information of this article and at: https://doi.org/10.6084/m9.figshare.30299914. Primary data were sourced from https://www.iucnredlist.org/resources/spatial-data-download (mammal geographic distributions), https://doi.org/10.1038/s41586-021-04341-1 (mammal phylogeny), https://doi.org/10.6084/m9.figshare.13028255.v4 (mammal traits), https://paleobiodb.org/#/ (mammal fossils), https://doi.org/10.1002/2017GC007273 (map of unconsolidated sediments). The data used to generate the figures are also available at: https://doi.org/10.6084/m9.figshare.30299914.

## Code availability

The code used in this study, included that to generate the figures, is available at: https://doi.org/10.6084/m9.figshare.30299914.
