# Supplementary material for: Fossil record biases skew inferences of mammal trait evolution
Source: Nat Commun. 2026 Aug 24;17:10096. doi: 10.1038/s41467-026-77026-w (PMC13597520; doi:10.1038/s41467-026-77026-w)
Supplement: Supplementary file 1 [file 41467_2026_77026_MOESM1_ESM.pdf]

Supplementary Information

Fossil record biases skew inferences of mammal trait evolution

Graciela Sotelo[1]*, Sofía Galván[1], Sara Gamboa[1,2], Adrián Castro-Insua[1], Alfio Alessandro Chiarenza[3], Emma M. Dunne[4], Lewis A. Jones[3], Marta Matamala-Pagès[1], Eduardo Méndez-Quintas[1], Adriana Oliver[1], Iván Rey-Rodríguez[1], Sara Varela[1,5]

*Corresponding Author: Graciela Sotelo
E-mail: gsotelo.fdz@gmail.com / graciela.sotelo@uvigo.es

This PDF file includes:

Supplementary Note 1

Supplementary Figures 1 to 16

Supplementary Note 1

Evolution of body mass as a discrete trait

When body mass was analysed as a discrete trait with three categories (i.e., smaller- [0 – 500 g], medium- [500.1 – 50,000 g], and larger-size [> 50,000 g]) it best adjusted to a kappa (punctuated/speciational evolution) model of evolution in the baseline phylogeny (Akaike weight = 0.89, κ = 0.73) under the equal-rates (ER) transition matrix (Supplementary Fig. 9b; Supplementary Data 6). This result was maintained across all filter and rate combinations despite support fluctuations over samples, being the lowest after range-size (Akaike weight ≤ 0.40). The independent approach showed that taxonomic filters had the largest impact on model selection. Taxonomy-whole families favoured delta (time-dependent evolution) (Akaike weight = 0.48) over the baseline kappa (Akaike weight = 0.15) in the 25 rate scenario and taxonomy-weighted favoured the null model (Akaike weight = 0.39) instead (kappa Akaike weight = 0.28) in the 50 rate scenario. In addition, range-size consistently lowered the support of the baseline model (Supplementary Fig. 9b, 15a).

According to the ER model, the mammal ancestor most likely belonged to the smaller-size category but estimates moved towards medium-sizes once the body-size filter was applied, especially in the 25 rate scenario (Supplementary Fig. 11, 12, 16a). This also happened for Rodentia, Lipotyphla, and Primates. For Chiroptera, the support of a medium ancestor increased as well but remained below that of the smaller. On the other hand, estimates for Carnivora were unaltered and those for Artiodactyla were only affected by the taxonomic filters, in favour of a larger-sized ancestor (Supplementary Fig. 11, 12).

Evolution of diet as a discrete trait

The inferred evolution of diet (i.e., herbivore, omnivore, and faunivore) along the mammal phylogeny adjusted to a kappa model (Akaike weight = 1, $\kappa = 0.23$) under the ER transition matrix (Supplementary Fig. 8c; Supplementary Data 7). The baseline results were only shifted after taxonomic filters (Akaike weight ≤ 0.24), in support of the null model (Akaike weight ≥ 0.38) in the 25 rate scenario, while the independent effect of the filters was largely negligible (Supplementary Fig. 9c, 15b).

Attending to the ER model, the mammal ancestor could belong to any of the three diet categories although the mean probability of faunivory was slightly higher (0.39 vs. 031 for omnivory and 0.29 for herbivory) in the baseline phylogeny (Supplementary Fig. 13, 14, 16b). This particularly changed after taxonomy-whole families in the 25 rate scenario, when herbivory was better supported instead (also in the independent approach), a trend observed already from body-size onwards. The pattern of state probabilities varied across orders. Herbivory was inferred as the most likely ancestral state for Rodentia, Primates, and Artiodactyla, while faunivory was so for Chiroptera and Lipotyphla, and omnivory and faunivory were similarly supported for Carnivora. The filter that most altered the results was range-size in the case of Rodentia and Primates, and body-size in the case of Chiroptera and Lipotyphla, while estimates for Artiodactyla were barely affected. For Carnivora, only the taxonomic filters modified the most likely state when applied independently in the 25 rate scenario (Supplementary Fig. 13, 14).

Supplementary Figures

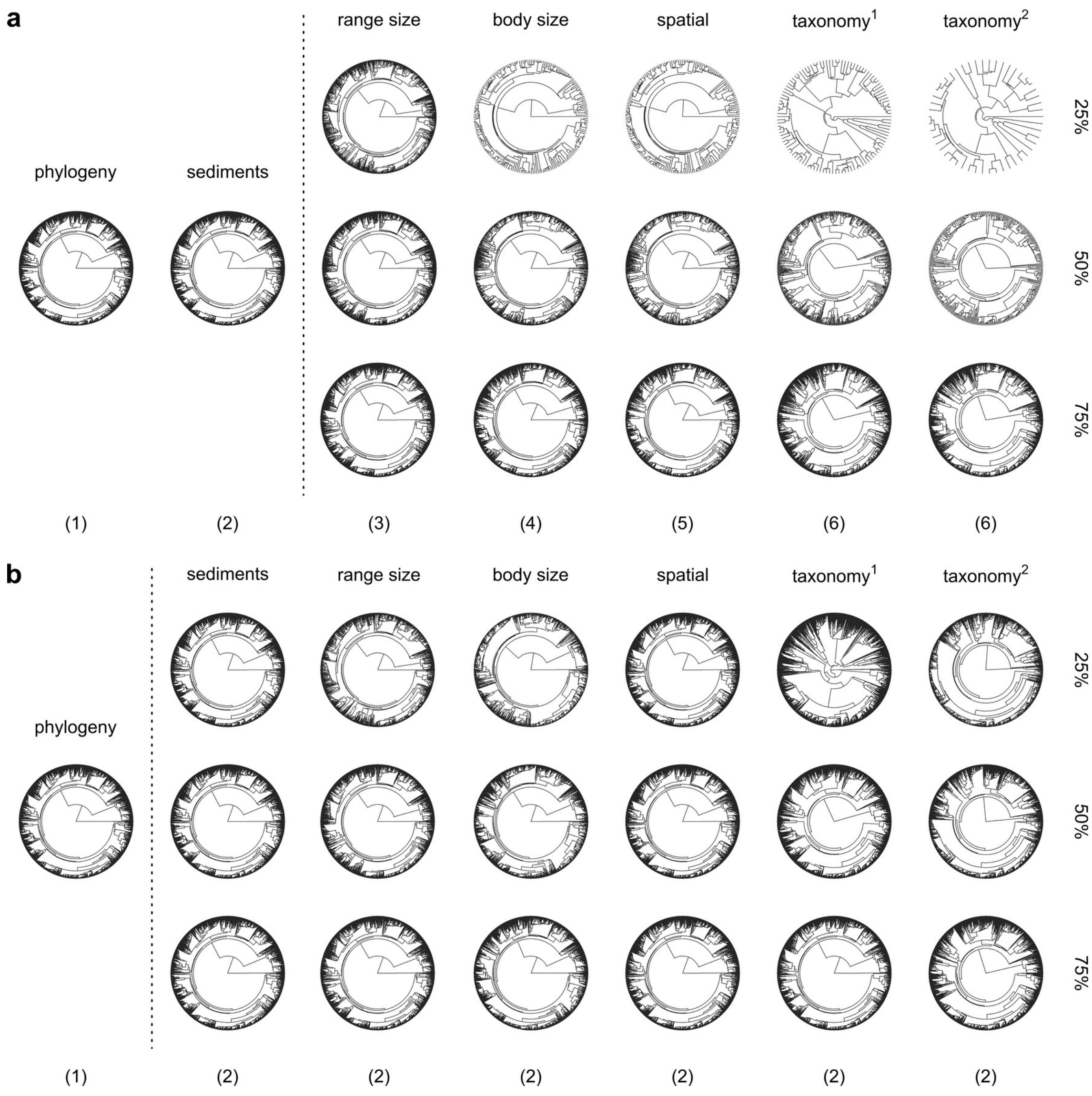

Supplementary Fig. 1. Phylogenies obtained for the first replicate after each filtering step and preservation rate (25%, 50%, 75%) in the consecutive (**a**) and independent (**b**) approaches. Abbreviations: taxonomy[1] – taxonomy-whole families filter, taxonomy[2] – taxonomy-weighted filter. Numbers in the bottom row indicate the order of application of filters.

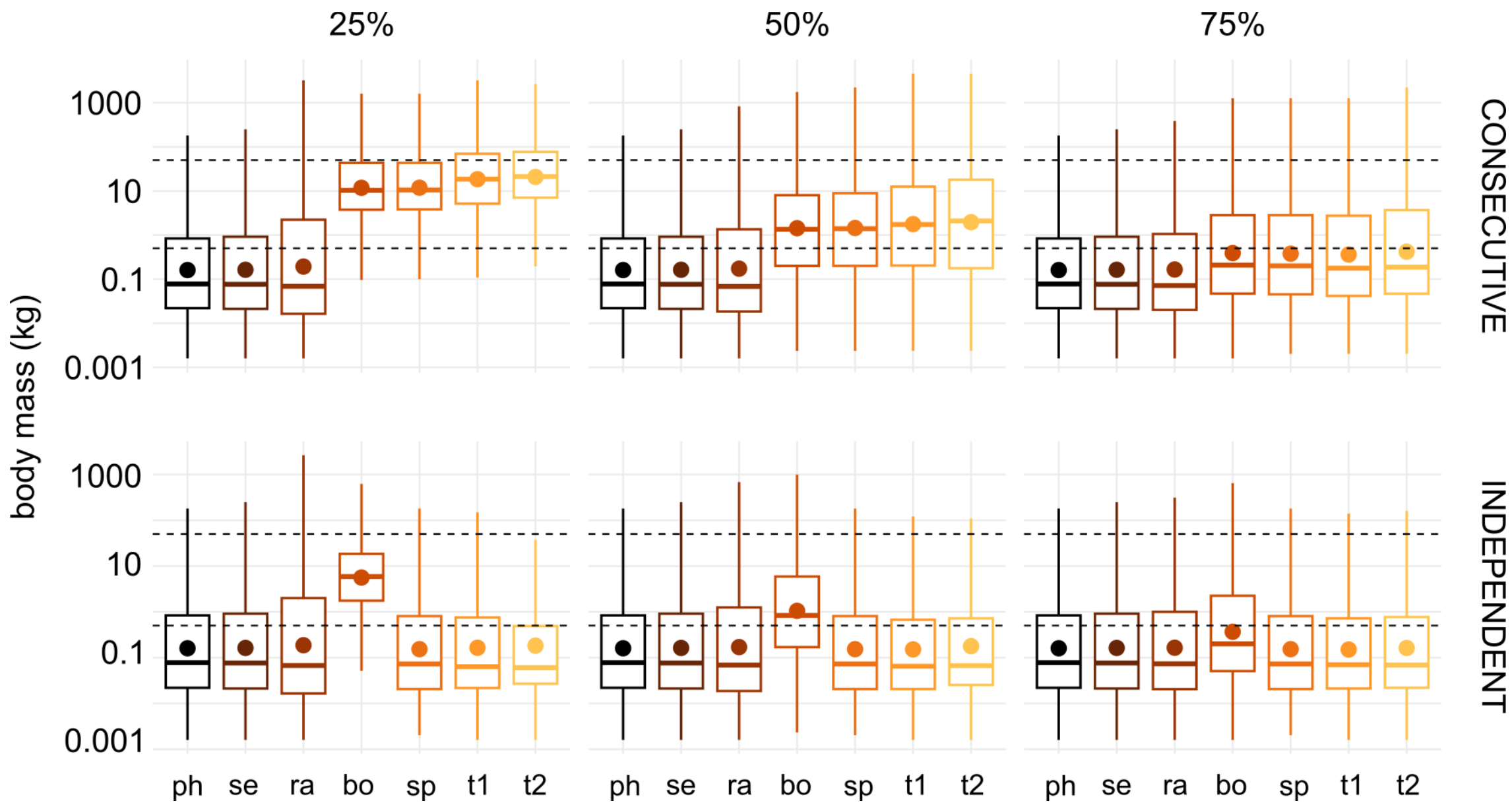

Supplementary Fig. 2. Body mass distributions after each filtering step and preservation rate (25%, 50%, 75%) across a hundred replicates, in the consecutive (top panel) and independent (bottom panel) approaches. Filter codes: ph – phylogeny, se – sediments, ra – range-size, bo – body-size, sp – spatial, t1 – taxonomy-whole families, t2 – taxonomy-weighted. Colours correspond to filters. Dots within boxes indicate mean body mass values; y-axis is shown in log10 scale. Dotted horizontal lines indicate the limits between larger- (> 50 kg), medium- (0.5 – 50 kg), and smaller-size (≤ 0.5 kg) body mass categories (from top to bottom), when body mass is taken as a discrete trait.

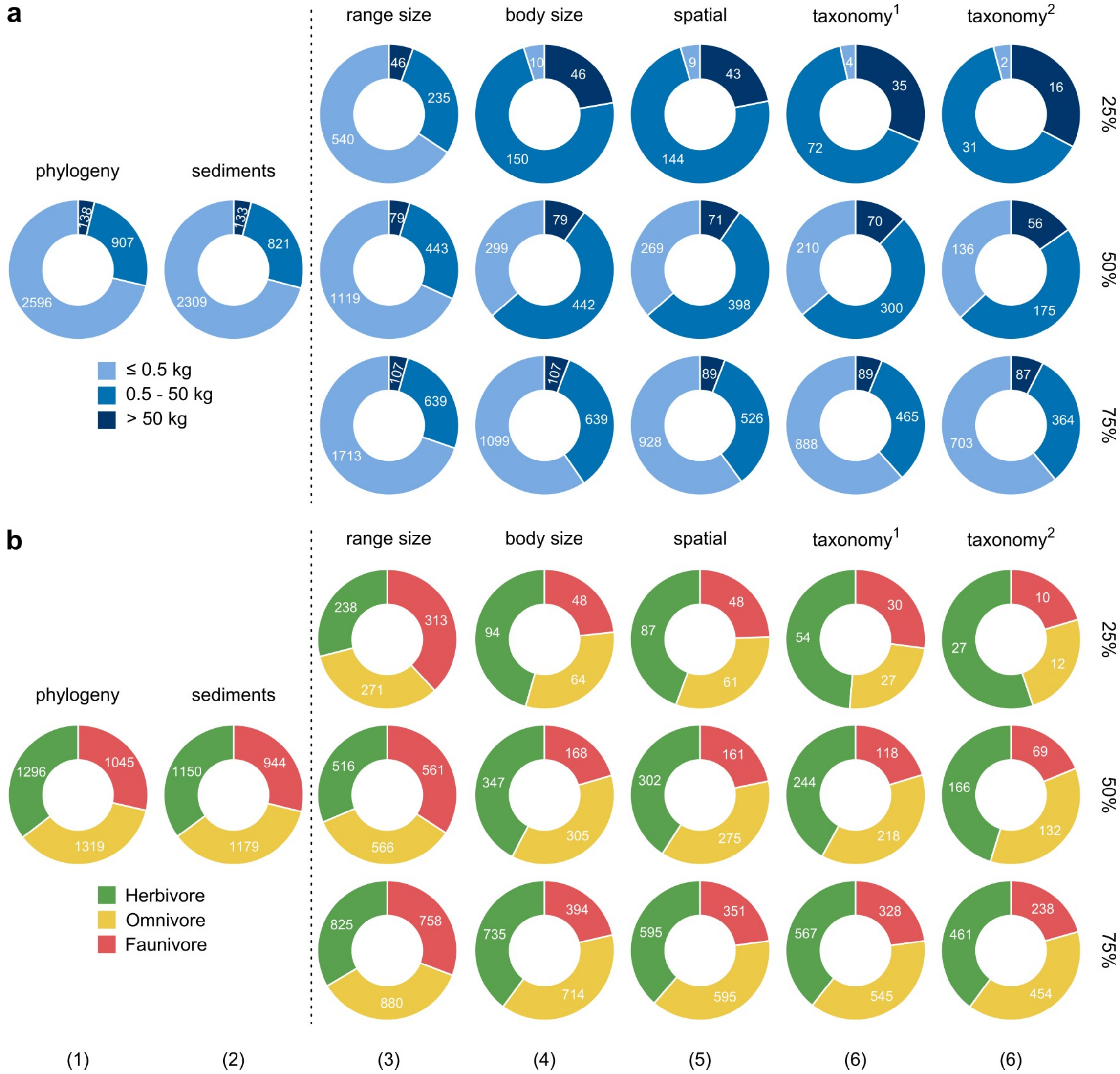

Supplementary Fig. 3. Sample composition by trait category in the consecutive approach, for body mass (**a**) and diet (**b**). Doughnut charts indicate the mean number of species retained after each filtering step and preservation rate (25%, 50%, 75%) across a hundred replicates. Abbreviations: taxonomy[1] – taxonomy-whole families filter, taxonomy[2] – taxonomy-weighted filter. Numbers in the bottom row indicate the order of application of filters.

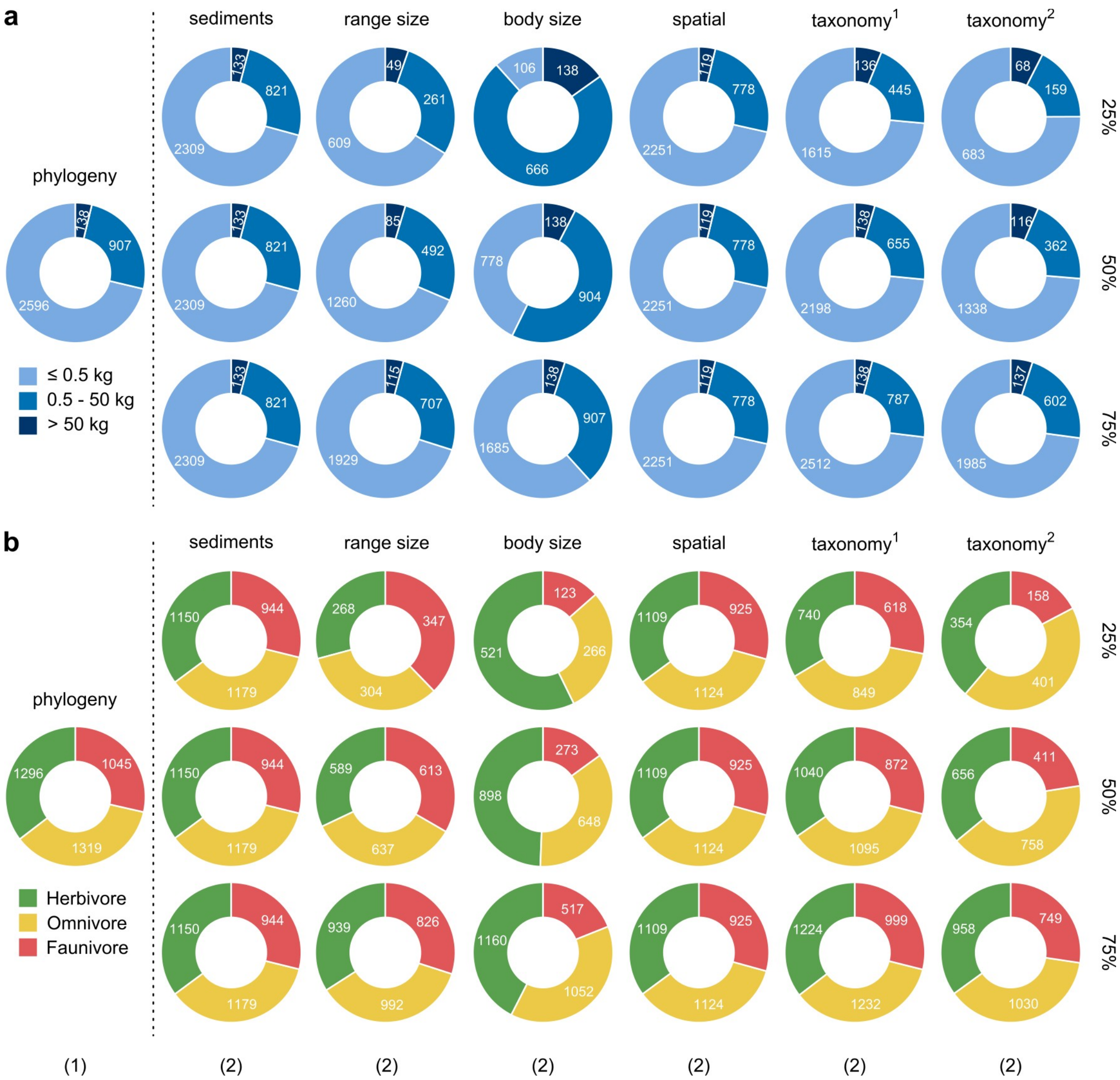

Supplementary Fig. 4. Sample composition by trait category in the independent approach, for body mass (**a**) and diet (**b**). Doughnut charts indicate the mean number of species retained after each filtering step and preservation rate (25%, 50%, 75%) across a hundred replicates. Abbreviations: taxonomy[1] – taxonomy-whole families filter, taxonomy[2] – taxonomy-weighted filter. Numbers in the bottom row indicate the order of application of filters.

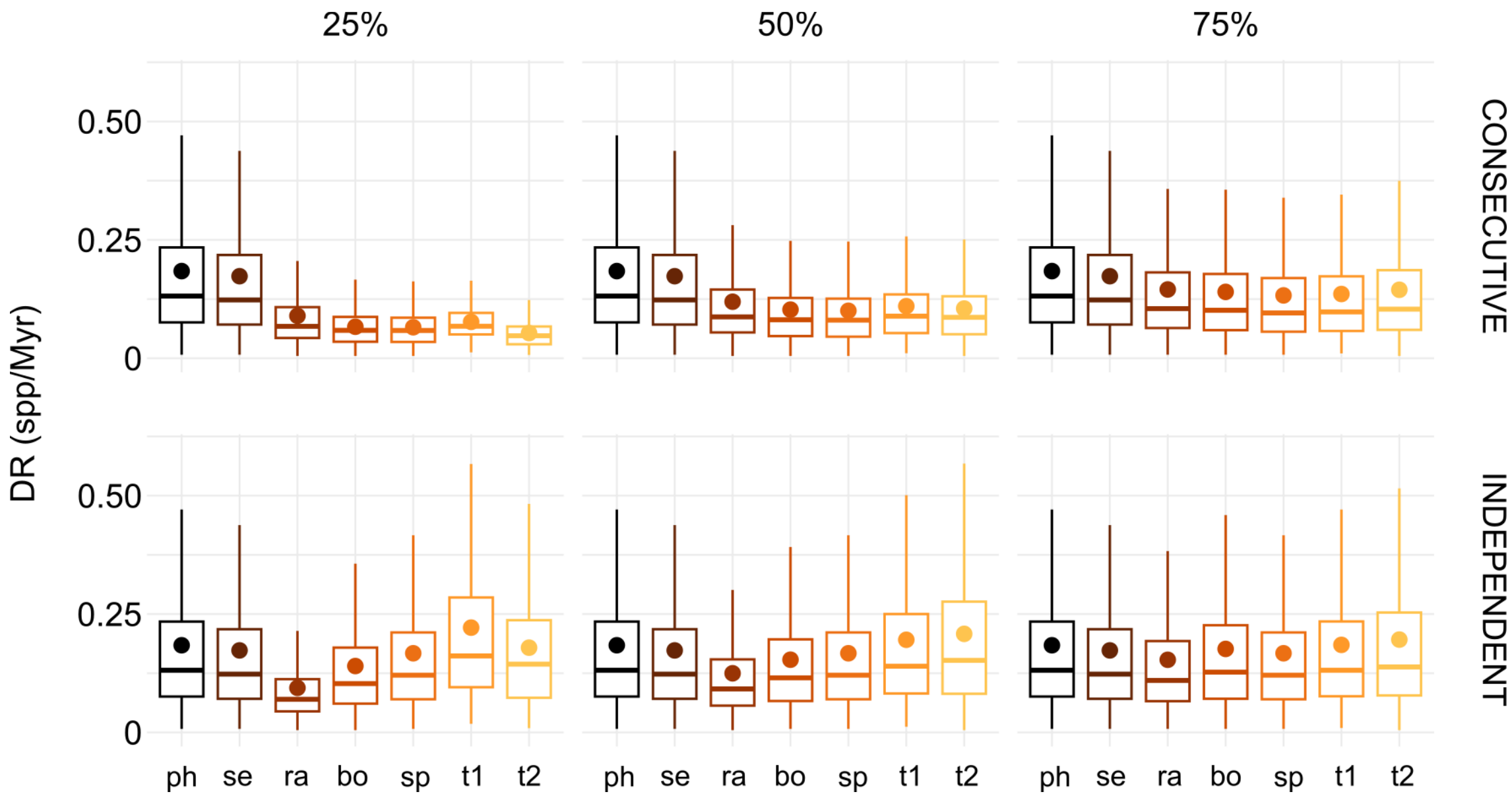

Supplementary Fig. 5. Distribution of tip diversification rate (DR) values after each filtering step and preservation rate (25%, 50%, 75%) across a hundred replicates, in the consecutive (top panel) and independent (bottom panel) approaches. Filter codes: ph – phylogeny, se – sediments, ra – range-size, bo – body-size, sp – spatial, t1 – taxonomy-whole families, t2 – taxonomy-weighted. Colours correspond to filters. Dots within boxes indicate mean DR values. DR estimates are indicated in species (spp) per one million years (Myr).

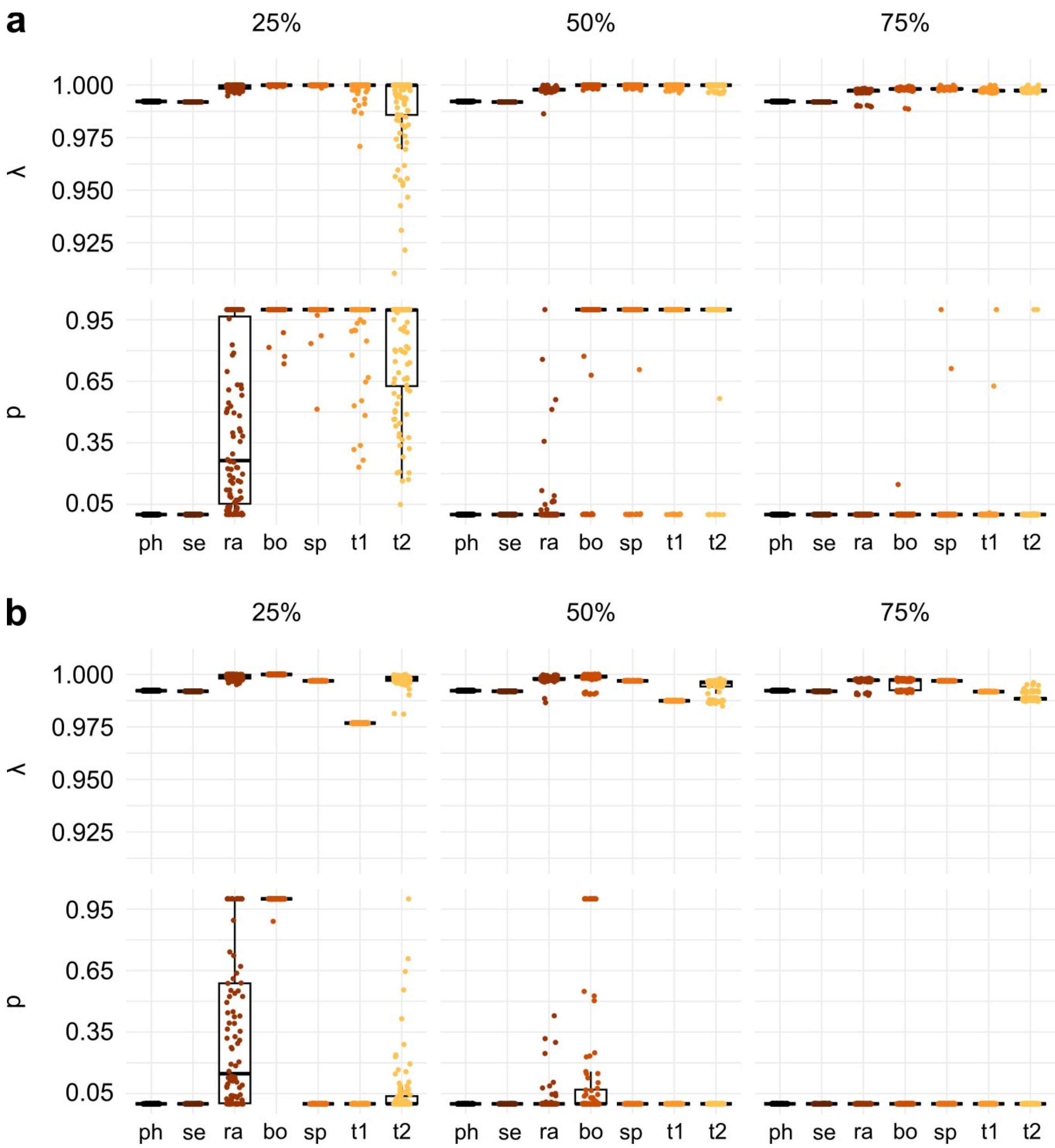

Supplementary Fig. 6. Phylogenetic signal of body mass as continuous trait after each filtering step and preservation rate (25%, 50%, 75%) in the consecutive (**a**) and independent (**b**) approaches. The lambda parameter (λ) and the corresponding *p*-value (p) for the null hypothesis of $\lambda = 1$ are shown in top and bottom panels, respectively. Filter codes: ph – phylogeny, se – sediments, ra – range-size, bo – body-size, sp – spatial, t1 – taxonomy-whole families, t2 – taxonomy-weighted. Colours correspond to filters.

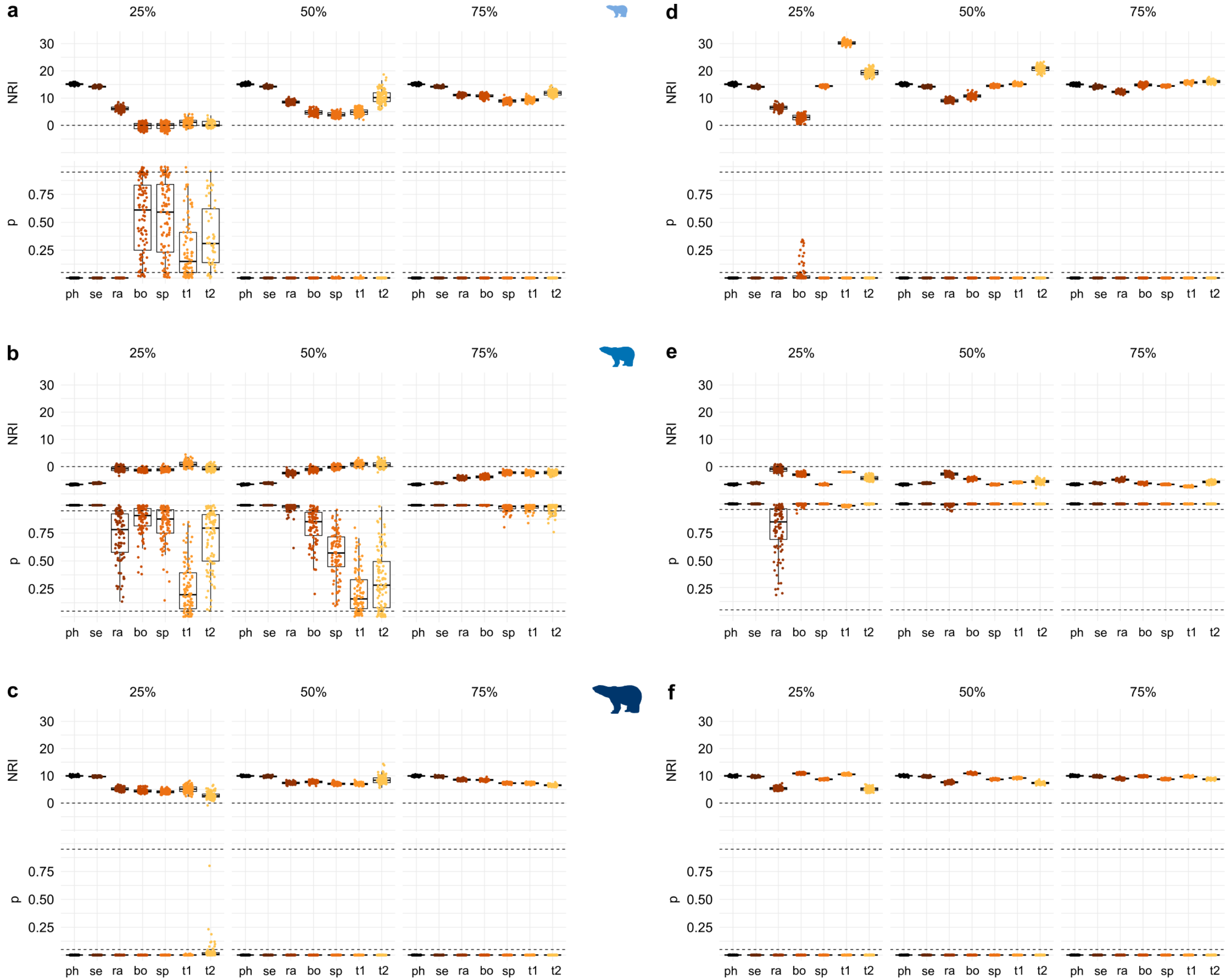
a
b
c
d
e
f
25%
50%
75%
NRI
p
30
20
10
0
0.75
0.50
0.25
ph
se
ra
bo
sp
t1
t2

Supplementary Fig. 7. Phylogenetic distribution of body mass categories after each filtering step and preservation rate (25%, 50%, 75%) in the consecutive (**a–c**) and independent (**d–f**) approaches. Filter codes: ph – phylogeny, se – sediments, ra – range-size, bo – body-size, sp – spatial, t1 – taxonomy-whole families, t2 – taxonomy-weighted. Colours correspond to filters. For each trait category (small: **a**, **d**; medium: **b**, **e**; large: **c**, **f**), the distributions of the net relatedness index (NRI) and corresponding *p*-values (p) are shown in top and bottom panels, respectively. On top panels, the horizontal dotted line indicates NRI value of zero. On bottom panels, horizontal dotted lines indicate *p*-values of 0.95 (upper) and 0.05 (lower). Positive NRI with *p*-value below 0.05 denotes clustering and negative NRI with *p*-value over 0.95, overdispersion. Remaining values denote random distribution. Species silhouettes were downloaded from PhyloPic (https://www.phylopic.org): *Ursus maritimus* by Tracy Heath is reprinted under a CC0 1.0 Universal Public Domain Dedication license.

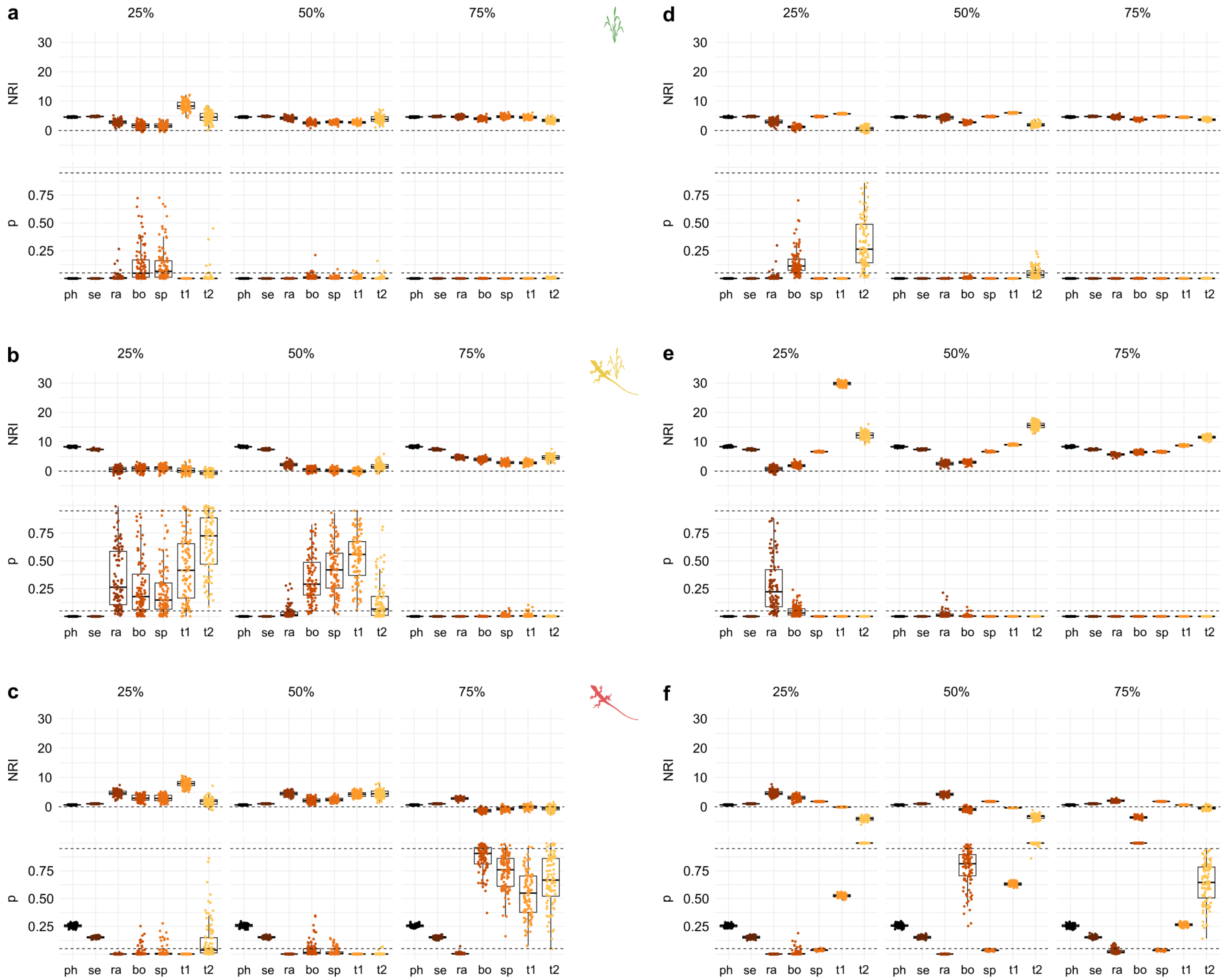

a
b
c
d
e
f
25%
50%
75%
NRI
p
30
20
10
0
0.75
0.50
0.25
ph
se
ra
bo
sp
t1
t2

Supplementary Fig. 8. Phylogenetic distribution of diet categories after each filtering step and preservation rate (25%, 50%, 75%) in the consecutive (**a**–**c**) and independent (**d**–**f**) approaches. Filter codes: ph – phylogeny, se – sediments, ra – range-size, bo – body-size, sp – spatial, t1 – taxonomy-whole families, t2 – taxonomy-weighted. Colours correspond to filters. For each trait category (herbivore: **a**, **d**; omnivore: **b**, **e**; faunivore: **c**, **f**), the distributions of the net relatedness index (NRI) and corresponding *p*-values (p) are shown in top and bottom panels, respectively. On top panels, the horizontal dotted line indicates NRI value of zero. On bottom panels, horizontal dotted lines indicate *p*-values of 0.95 (upper) and 0.05 (lower). Positive NRI with *p*-value below 0.05 denotes clustering and negative NRI with *p*-value over 0.95, overdispersion. Remaining values denote random distribution. Species silhouettes were downloaded from PhyloPic (https://www.phylopic.org): *Triticum aestivum aestivum* by Mason McNair (**a**, **d**–**b**, **e**) and *Anolis carolinensis* by Christoph Schomburg (**b**, **e**–**c**, **f**) are reprinted under a CC0 1.0 Universal Public Domain Dedication license.

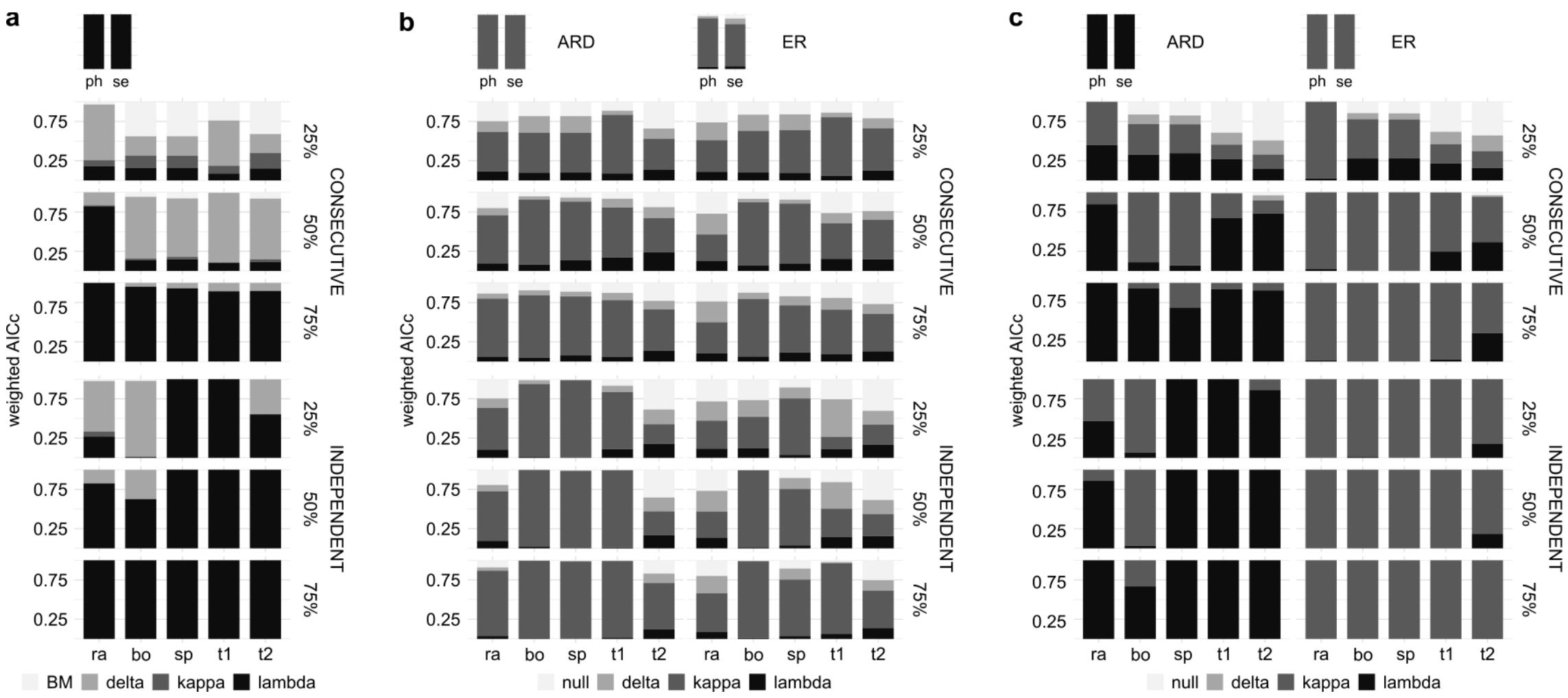

Supplementary Fig. 9. Mean support of models of trait evolution after each filtering step and preservation rate (25%, 50%, 75%) in the consecutive (top panels) and independent (bottom panels) approaches. Results are shown for body mass as continuous (log10 transformed, **a**) and discrete (three categories, **b**) trait, and for diet as discrete trait (three categories, **c**). Model support is indicated as mean weighted AICc scores across a hundred replicates. Filter codes: ph – phylogeny, se – sediments, ra – range-size, bo – body-size, sp – spatial, t1 – taxonomy-whole families, t2 – taxonomy-weighted. Abbreviations: BM – Brownian-Motion model; ARD – all-rates-different transition model; ER – equal-rates transition model.

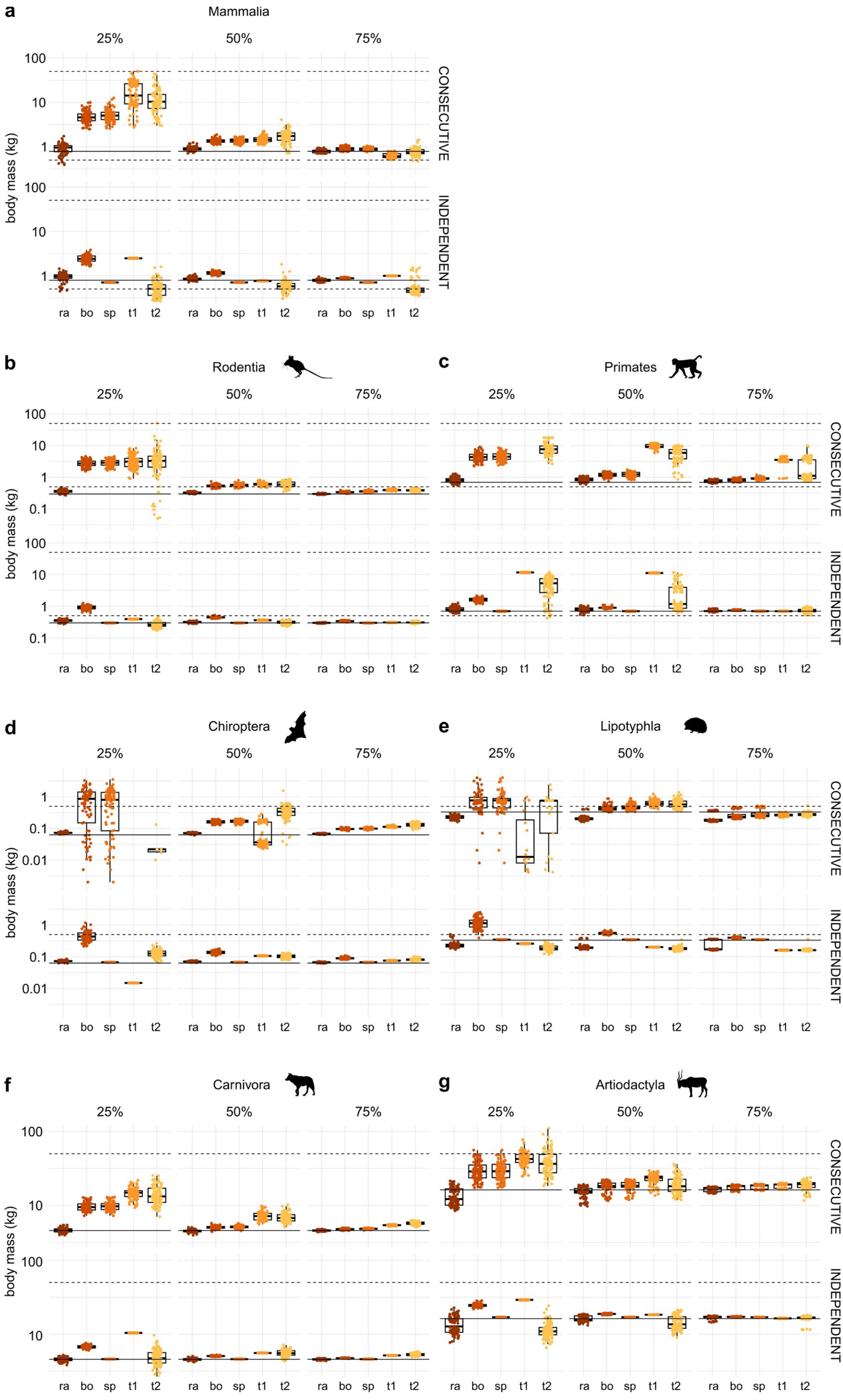
a
Mammalia
25%
50%
75%
body mass (kg)
100
10
1
CONSECUTIVE
INDEPENDENT
ra
bo
sp
t1
t2
b
Rodentia
0.1
c
Primates
d
Chiroptera
0.01
e
Lipotyphla
f
Carnivora
g
Artiodactyla

Supplementary Fig. 10. Distribution of inferred body mass for the ancestor of all mammals (**a**) and of major mammal orders (**b–g**) after each filtering step and preservation rate (25%, 50%, 75%) in the consecutive (top panels) and independent (bottom panels) approaches; y-axes are shown in log10 scale. The solid horizontal line indicates the ancestral estimate from the baseline phylogeny. Dotted horizontal lines indicate the limits between larger-, medium-, and smaller-size body mass categories (when body mass is taken as a discrete trait) as appropriate. Filter codes: ra – range-size, bo – body-size, sp – spatial, t1 – taxonomy-whole families, t2 – taxonomy-weighted. Colours correspond to filters. The sediments filter is not included because its results adjust to the baseline. Species silhouettes were downloaded from PhyloPic (https://www.phylopic.org): *Notomys mitchellii* by Rachel T Mason is reprinted under a CC0 1.0 Universal Public Domain Dedication license (**b**), *Macaca mulatta* by Ben Murrell is reprinted under a CC0 1.0 Universal Public Domain Dedication license (**c**), *Lasiurus borealis* by CJ Campbell is reprinted under a CC0 1.0 Universal Public Domain Dedication license (**d**), *Atelerix albiventris* by Motzillian is reprinted under a CC0 1.0 Universal Public Domain Dedication license (**e**), *Canis lupus* by Margot Michaud is reprinted under a CC0 1.0 Universal Public Domain Dedication license (**f**), *Addax nasomaculatus* by Andy Wilson is reprinted under a CC0 1.0 Universal Public Domain Dedication license (**g**).

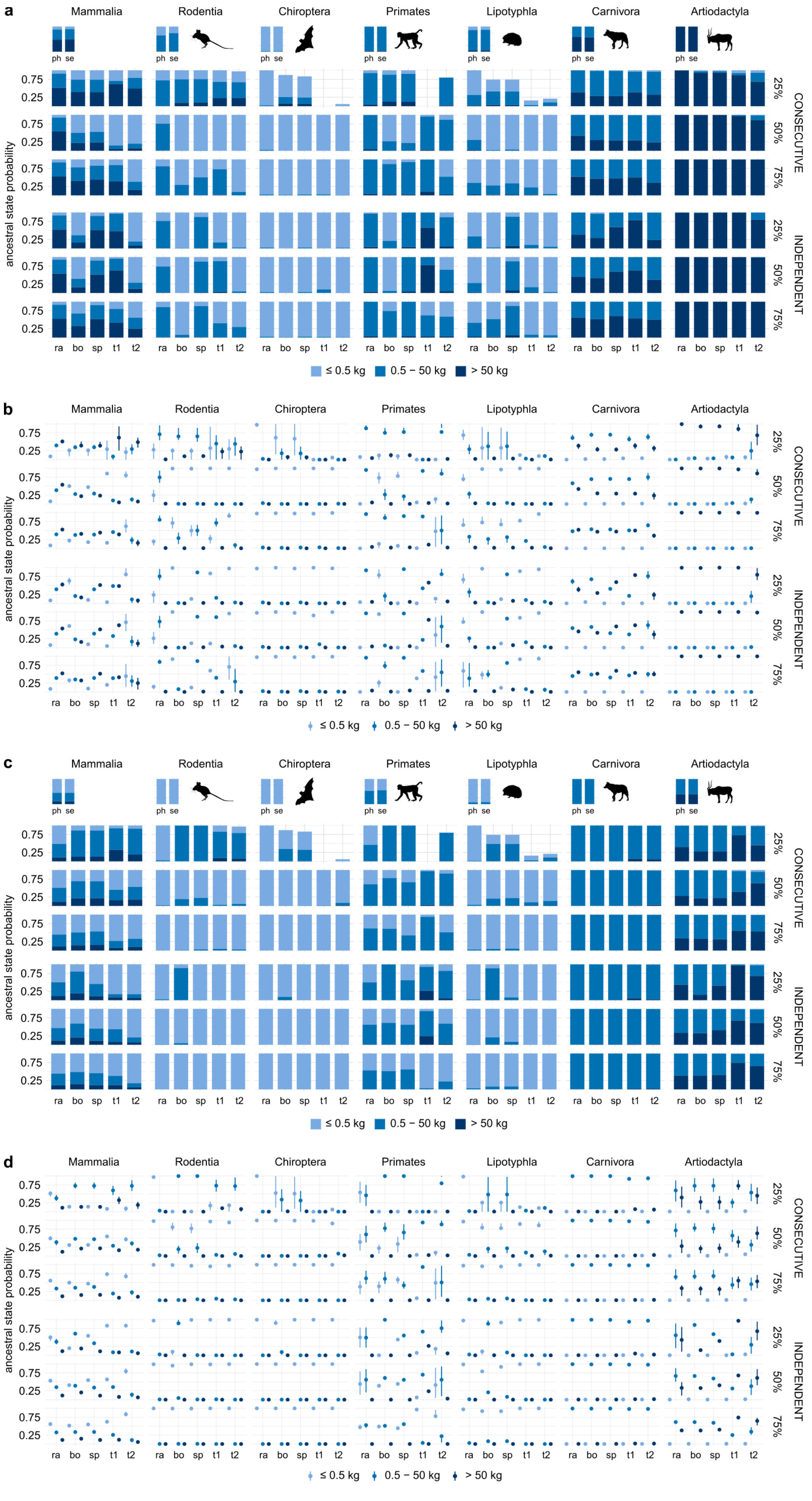
a
b
c
d
Mammalia
Rodentia
Chiroptera
Primates
Lipotyphla
Carnivora
Artiodactyla
ph se
ancestral state probability
0.75
0.25
ra bo sp t1 t2
25%
50%
75%
CONSECUTIVE
INDEPENDENT
≤ 0.5 kg
0.5 − 50 kg
> 50 kg

Supplementary Fig. 11. Maximum likelihood ancestral character reconstruction of body mass as a discrete trait, for the mammal root and the ancestor of each major mammal order, per filtering step and preservation rate (25%, 50%, 75%) in the consecutive (top panels) and independent (bottom panels) approaches across one hundred replicates. **a**, **c**) Mean probability of each character state under ARD (all-rates-different) and ER (equal-rates) transition models, respectively. Unfilled bars correspond to simulated samples missing species from the corresponding order. **b**, **d**) Mean probability (dot) and interquartile range (vertical line) of each character state under ARD (all rates different) and ER (equal rates) transition models, respectively. Filter codes: ph – phylogeny, se – sediments, ra – range-size, bo – body-size, sp – spatial, t1 – taxonomy-whole families, t2 – taxonomy-weighted. Species silhouettes were downloaded from PhyloPic (https://www.phylopic.org): *Notomys mitchellii* by Rachel T Mason is reprinted under a CC0 1.0 Universal Public Domain Dedication license, *Lasiurus borealis* by CJ Campbell is reprinted under a CC0 1.0 Universal Public Domain Dedication license, *Macaca mulatta* by Ben Murrell is reprinted under a CC0 1.0 Universal Public Domain Dedication license, *Atelerix albiventris* by Motzillian is reprinted under a CC0 1.0 Universal Public Domain Dedication license, *Canis lupus* by Margot Michaud is reprinted under a CC0 1.0 Universal Public Domain Dedication license, *Addax nasomaculatus* by Andy Wilson is reprinted under a CC0 1.0 Universal Public Domain Dedication license (from left to right; **a**, **c**).

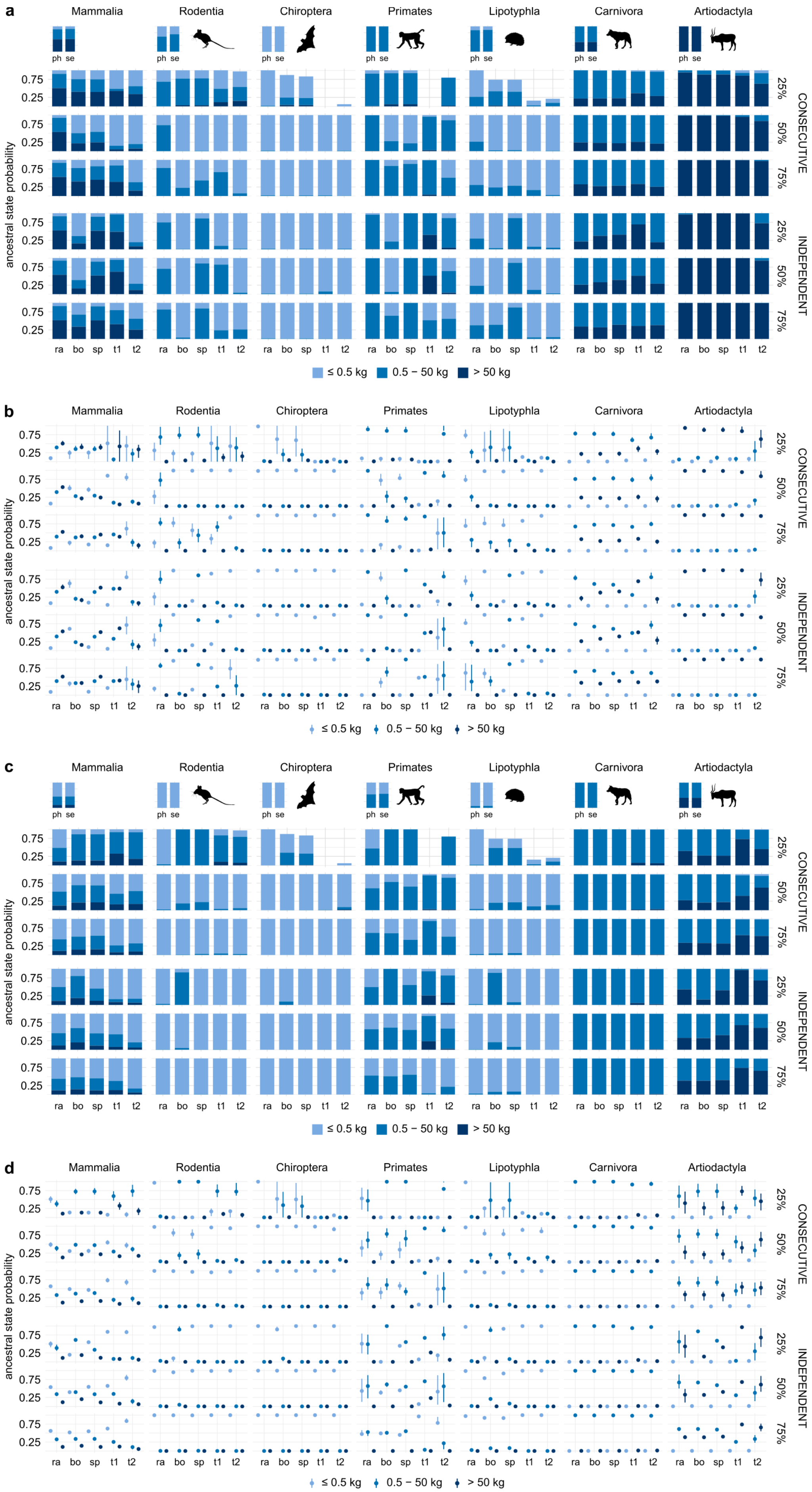
a
b
c
d
Mammalia
Rodentia
Chiroptera
Primates
Lipotyphla
Carnivora
Artiodactyla
ph se
ancestral state probability
0.75
0.25
25%
50%
75%
CONSECUTIVE
INDEPENDENT
ra bo sp t1 t2
≤ 0.5 kg
0.5 − 50 kg
> 50 kg

Supplementary Fig. 12. Bayesian ancestral character reconstruction of body mass as a discrete trait, for the mammal root and the ancestor of each major mammal order, per filtering step and preservation rate (25%, 50%, 75%) in the consecutive (top panels) and independent (bottom panels) approaches across one hundred replicates. **a**, **c**) Mean probability of each character state under ARD (all-rates-different) and ER (equal-rates) transition models, respectively. Unfilled bars correspond to simulated samples missing species from the corresponding order. **b**, **d**) Mean probability (dot) and interquartile range (vertical line) of each character state under ARD (all-rates-different) and ER (equal-rates) transition models, respectively. Filter codes: ph – phylogeny, se – sediments, ra – range-size, bo – body-size, sp – spatial, t1 – taxonomy-whole families, t2 – taxonomy-weighted. Species silhouettes were downloaded from PhyloPic (https://www.phylopic.org): *Notomys mitchellii* by Rachel T Mason is reprinted under a CC0 1.0 Universal Public Domain Dedication license, *Lasiurus borealis* by CJ Campbell is reprinted under a CC0 1.0 Universal Public Domain Dedication license, *Macaca mulatta* by Ben Murrell is reprinted under a CC0 1.0 Universal Public Domain Dedication license, *Atelerix albiventris* by Motzillian is reprinted under a CC0 1.0 Universal Public Domain Dedication license, *Canis lupus* by Margot Michaud is reprinted under a CC0 1.0 Universal Public Domain Dedication license, *Addax nasomaculatus* by Andy Wilson is reprinted under a CC0 1.0 Universal Public Domain Dedication license (from left to right; **a**, **c**).

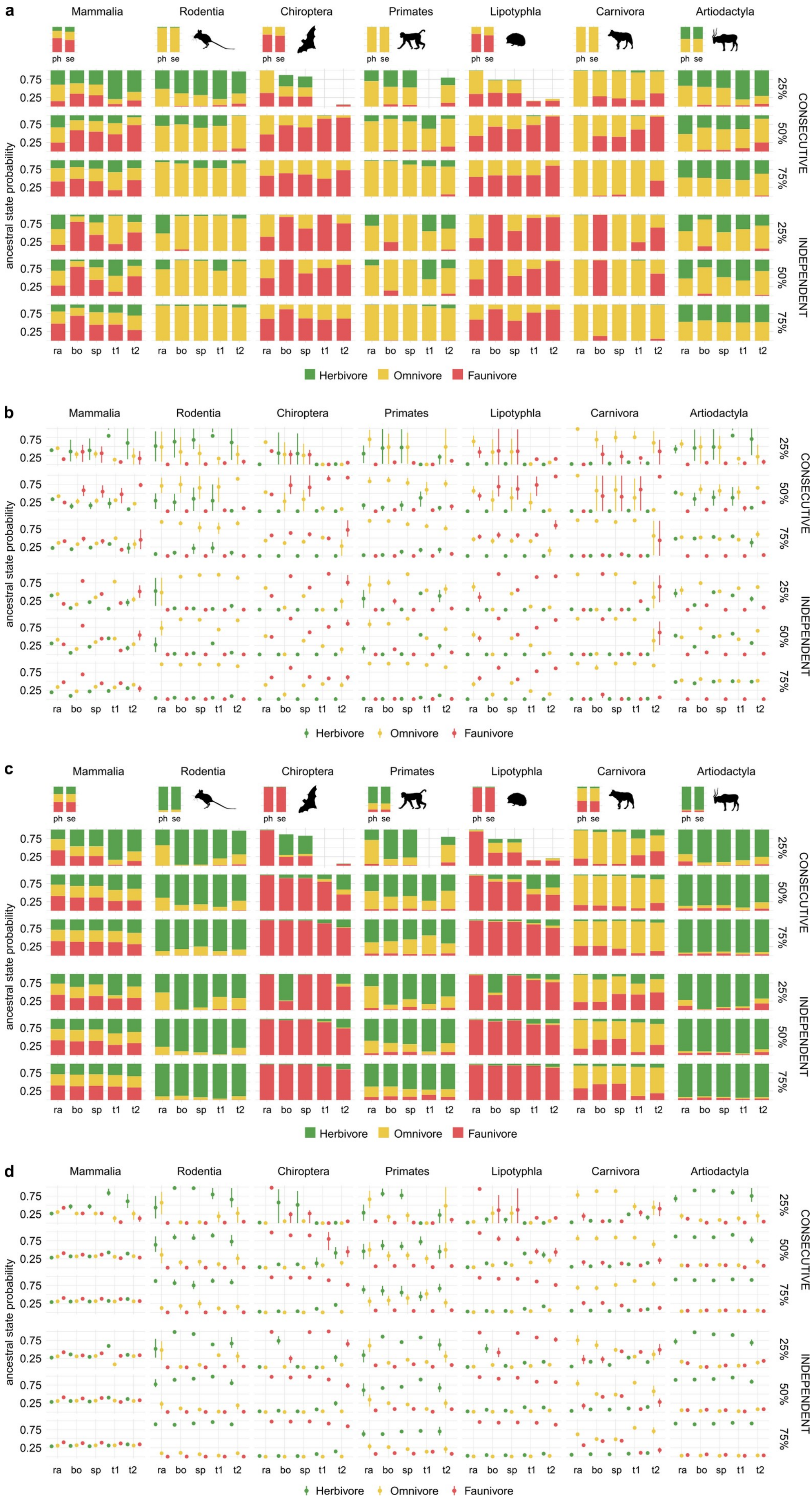
a
Mammalia
Rodentia
Chiroptera
Primates
Lipotyphla
Carnivora
Artiodactyla
ph se
0.75
0.25
ancestral state probability
ra bo sp t1 t2
25%
50%
75%
CONSECUTIVE
INDEPENDENT
Herbivore
Omnivore
Faunivore
b
c
d

Supplementary Fig. 13. Maximum likelihood ancestral character reconstruction of diet, for the mammal root and the ancestor of each major mammal order, per filtering step and preservation rate (25%, 50%, 75%) in the consecutive (top panels) and independent (bottom panels) approaches across one hundred replicates. **a**, **c**) Mean probability of each character state under ARD (all-rates-different) and ER (equal-rates) transition models, respectively. Unfilled bars correspond to simulated samples missing species from the corresponding order. **b**, **d**) Mean probability (dot) and interquartile range (vertical line) of each character state under ARD (all-rates-different) and ER (equal-rates) transition models, respectively. Filter codes: ph – phylogeny, se – sediments, ra – range-size, bo – body-size, sp – spatial, t1 – taxonomy-whole families, t2 – taxonomy-weighted. Species silhouettes were downloaded from PhyloPic (https://www.phylopic.org): *Notomys mitchellii* by Rachel T Mason is reprinted under a CC0 1.0 Universal Public Domain Dedication license, *Lasiurus borealis* by CJ Campbell is reprinted under a CC0 1.0 Universal Public Domain Dedication license, *Macaca mulatta* by Ben Murrell is reprinted under a CC0 1.0 Universal Public Domain Dedication license, *Atelerix albiventris* by Motzillian is reprinted under a CC0 1.0 Universal Public Domain Dedication license, *Canis lupus* by Margot Michaud is reprinted under a CC0 1.0 Universal Public Domain Dedication license, *Addax nasomaculatus* by Andy Wilson is reprinted under a CC0 1.0 Universal Public Domain Dedication license (from left to right; **a**, **c**).

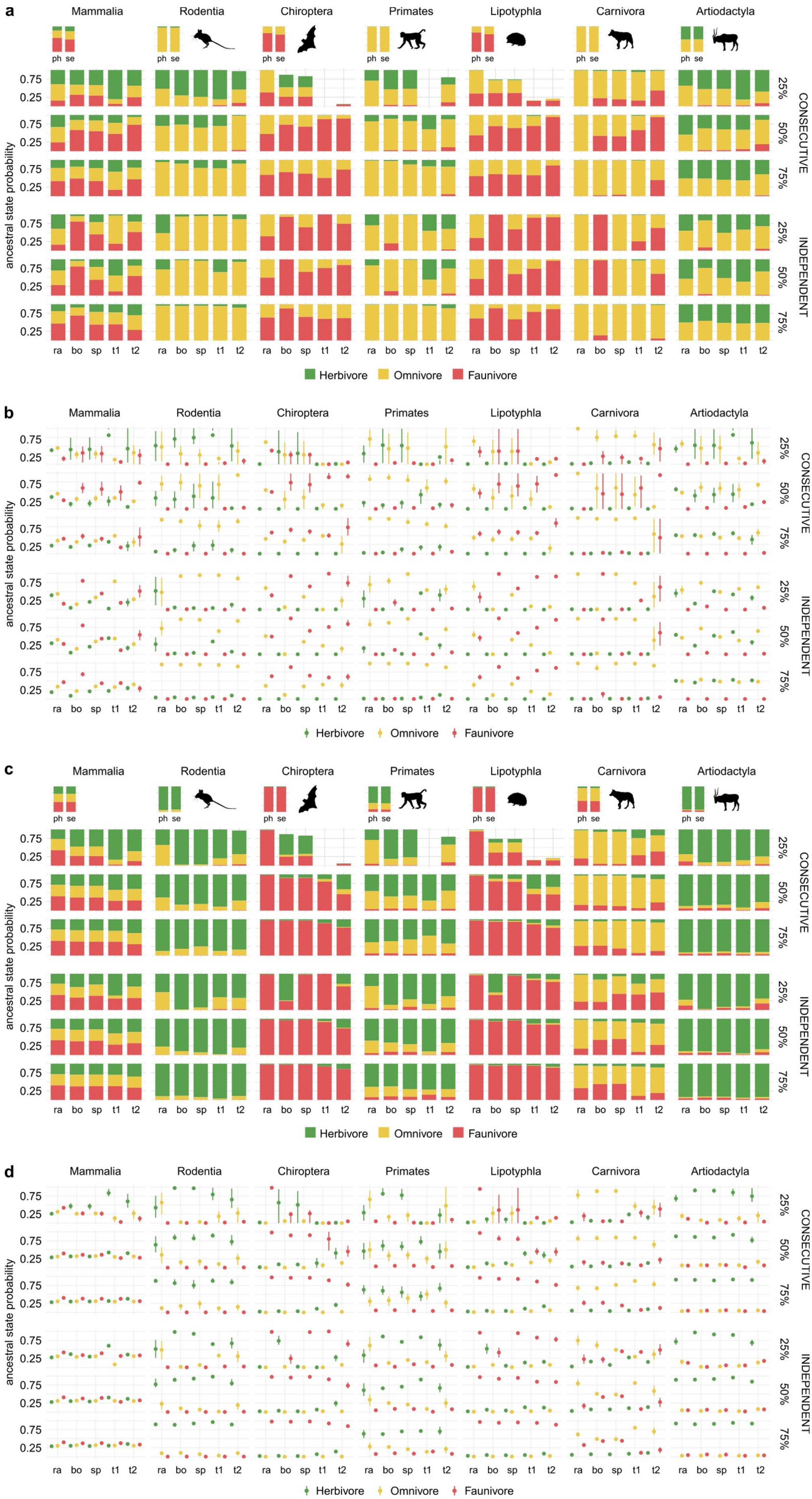
a
Mammalia
Rodentia
Chiroptera
Primates
Lipotyphla
Carnivora
Artiodactyla
ph se
ancestral state probability
0.75
0.25
ra bo sp t1 t2
25%
50%
75%
CONSECUTIVE
INDEPENDENT
Herbivore Omnivore Faunivore
b
c
d

Supplementary Fig. 14. Bayesian ancestral character reconstruction of diet, for the mammal root and the ancestor of each major mammal order, per filtering step and preservation rate (25%, 50%, 75%) in the consecutive (top panels) and independent (bottom panels) approaches across one hundred replicates. **a**, **c**) Mean probability of each character state under ARD (all-rates-different) and ER (equal-rates) transition models, respectively. Unfilled bars correspond to simulated samples missing species from the corresponding order. **b**, **d**) Mean probability (dot) and interquartile range (vertical line) of each character state under ARD (all-rates-different) and ER (equal-rates) transition models, respectively. Filter codes: ph – phylogeny, se – sediments, ra – range-size, bo – body-size, sp – spatial, t1 – taxonomy-whole families, t2 – taxonomy-weighted. Species silhouettes were downloaded from PhyloPic (https://www.phylopic.org): *Notomys mitchellii* by Rachel T Mason is reprinted under a CC0 1.0 Universal Public Domain Dedication license, *Lasiurus borealis* by CJ Campbell is reprinted under a CC0 1.0 Universal Public Domain Dedication license, *Macaca mulatta* by Ben Murrell is reprinted under a CC0 1.0 Universal Public Domain Dedication license, *Atelerix albiventris* by Motzillian is reprinted under a CC0 1.0 Universal Public Domain Dedication license, *Canis lupus* by Margot Michaud is reprinted under a CC0 1.0 Universal Public Domain Dedication license, *Addax nasomaculatus* by Andy Wilson is reprinted under a CC0 1.0 Universal Public Domain Dedication license (from left to right; **a**, **c**).

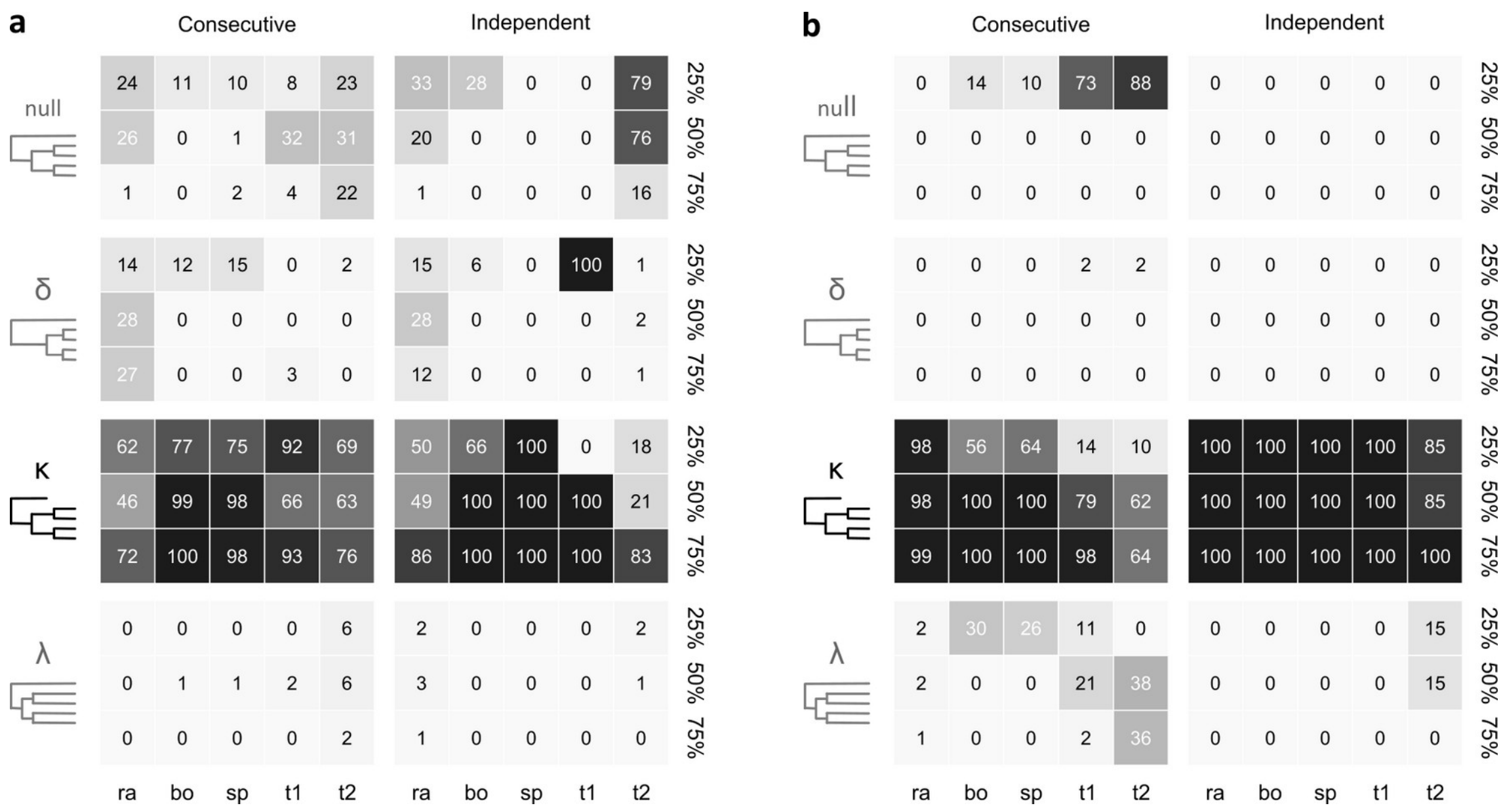

Supplementary Fig. 15. Summary of model support for the evolution of body mass (**a**) and diet (**b**) as discrete traits, under an equal-rates (ER) transition model. Heatmaps indicate the number of replicates, out of a hundred, where the best supported model was that shown in the left margin. null: none tree transformation, δ: delta (time-dependent), κ: kappa (speciational), λ: lambda (covariation) models. Results are indicated per filtering step and preservation rate (25%, 50%, 75%) in the consecutive (left column) and independent (right column) approaches. In the margin, the inference from the baseline phylogeny is highlighted in black. Filter codes: ra – range-size, bo – body-size, sp – spatial, t1 – taxonomy-whole families, t2 – taxonomy-weighted. The sediments filter is not included because of a consistent match with the baseline result.

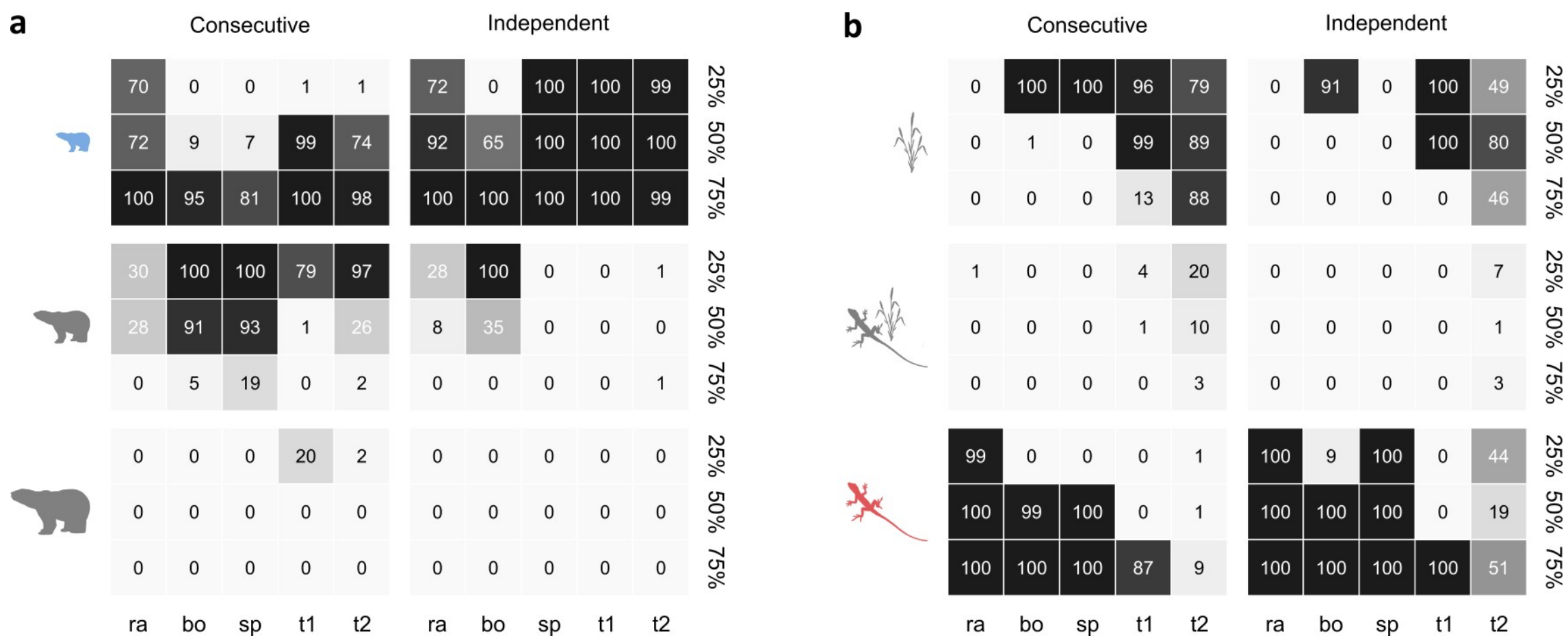

Supplementary Fig. 16. Summary of maximum likelihood ancestral character reconstruction of body mass (**a**) and diet (**b**), as discrete traits, under an equal-rates (ER) transition model, for the mammal ancestor. Heatmaps indicate the number of replicates, out of a hundred, where the most likely ancestral state was that shown in the margin (from top to bottom, small, medium, and large ancestor). Results are indicated per filtering step and preservation rate (25%, 50%, 75%) in the consecutive (left column) and independent (right column) approaches. In the left margin, the inference from the baseline phylogeny is highlighted in colour (in contrast to the general grayscale). Filter codes: ra – range-size, bo – body-size, sp – spatial, t1 – taxonomy-whole families, t2 – taxonomy-weighted. The sediments filter is not included because of a consistent match with the baseline result. Species silhouettes were downloaded from PhyloPic (https://www.phylopic.org): *Ursus maritimus* by Tracy Heath is reprinted under a CC0 1.0 Universal Public Domain Dedication license (**a**); *Triticum aestivum aestivum* by Mason McNair and *Anolis carolinensis* by Christoph Schomburg are reprinted under a CC0 1.0 Universal Public Domain Dedication license (**b**).
