# Supplementary material for: Fossil record biases skew inferences of mammal trait evolution
Source: Nat Commun. 2026 Aug 24;17:10096. doi: 10.1038/s41467-026-77026-w (PMC13597520; doi:10.1038/s41467-026-77026-w)
Supplement: Supplementary file 2 [file 41467_2026_77026_MOESM2_ESM.pdf]

Description of Additional Supplementary Files

Supplementary Data 1. Number of species retained after each filter and preservation rate in the consecutive and independent approaches. In the consecutive approach, mean, median, minimum (min), maximum (max), and standard deviation (sd) are indicated for each combination. In the independent approach, there is no variation in the number of species (n) across replicates.

Supplementary Data 2. Number of species with data on body mass retained after each filter and preservation rate in the consecutive and independent approaches. For each mammal order with more than 200 species in the baseline phylogeny, mean, median, minimum (min), maximum (max), and standard deviation (sd) are indicated per combination.

Supplementary Data 3. Number of species with data on diet retained after each filter and preservation rate in the consecutive and independent approaches. For each mammal order with more than 200 species in the baseline phylogeny, mean, median, minimum (min), maximum (max), and standard deviation (sd) are indicated per combination.

Supplementary Data 4. Differences in mean and median tip diversification rates (DR values) between the baseline phylogeny and pruned phylogenies after each filter and preservation rate in the consecutive and independent approaches. All differences respect to the baseline are significant (p-value < 1e-9) except for taxonomy-whole families filter under 75% preservation rate in the independent approach (p-value = 0.12; two-sided pairwise Wilcoxon test, significance level controlled for false discovery rate).

Supplementary Data 5. Summary of models and corresponding parameters of (continuous) body mass evolution after each filter and preservation rate in the consecutive and independent approaches. Best model: model with highest weighted AICc score in a set of replicates (n samples, out of a hundred). Mean, median, minimum (min), maximum (max), and standard deviation (sd) are indicated for each best model across the replicates it was selected as such.

Supplementary Data 6. Summary of models and corresponding parameters of (discrete) body mass evolution after each filter and preservation rate in the consecutive and independent

approaches. Matrix: transition matrix considered in each case; ARD: all rates different; ER: equal rates. Best model: model with highest weighted AICc score in a set of replicates (n samples, out of a hundred). Mean, median, minimum (min), maximum (max), and standard deviation (sd) are indicated for each best model across the replicates it was selected as such.

Supplementary Data 7. Summary of models and corresponding parameters of (discrete) diet evolution after each filter and preservation rate in the consecutive and independent approaches. Matrix: transition matrix considered in each case; ARD: all rates different; ER: equal rates. Best model: model with highest weighted AICc score in a set of replicates (n samples, out of a hundred). Mean, median, minimum (min), maximum (max), and standard deviation (sd) are indicated for each best model across the replicates it was selected as such.

Supplementary Data 8. List of synonymies applied between distribution (sci_name_IUCN) and phylogeny (sci_name_phylogeny) data.
