# Supplementary material for: Fossil record biases skew inferences of mammal trait evolution
Source: Nat Commun. 2026 Aug 24;17:10096. doi: 10.1038/s41467-026-77026-w (PMC13597520; doi:10.1038/s41467-026-77026-w)
Supplement: Supplementary file 4 [file 41467_2026_77026_MOESM5_ESM.pdf]

nature portfolio

Peer Review File

Fossil record biases skew inferences of mammal trait evolution

Corresponding Author: Dr Graciela Sotelo

This file contains all reviewer reports in order by version, followed by all author rebuttals in order by version.

Version 0:

Reviewer comments:

Reviewer #1

(Remarks to the Author)
This paper presents a simulations-based study aimed at understanding the impacts of various geological, taphonomic, and taxon-based 'filters' that affect the preservation potential of different terrestrial mammals, on reconstructing some key macroevolutionary 'traits'…including 'modes' of evolution, diversification rates, and the ancestral body mass and diet state. The authors consider a range of both biotic and abiotic factors; biotic factors include things like body size and range size (the idea being that small-bodied and -ranged taxa tend to have lower preservation probabilities), while abiotic factors include whether or not potential fossil 'localities' overlap with the species range – with localities (i.e., potentially fossiliferous rock) defined on the basis of unconsolidated sediments – and whether or not we're likely to find them (using the current locations of mammalian fossil sites downloaded from the PBDB – reflecting the fact that there is a geographic bias in paleontological exploration and sampling efforts). Finally, the authors impose a 'taxonomic' filter, tossing out species (essentially – counting them as 'not found') either on the basis on having low numbers of observations in the fossil record, or belonging to poorly-sampled families…the logic being, that paleontologists tend to work on some clades more than others. The results of the analysis show– perhaps not surprisingly – that the biological filters skew the distributions of body mass and diet, thus introducing errors into inference of ancestral state…while the abiotic factors tend to have much lesser impacts.

There are a growing number of simulations-based studies like this one, and the approach taken – one that aims to reconstruct evolutionary traits, rather than biogeographic patterns or extinction signal – is welcome and novel. That said, I have some reservations about the paper that stem from some of the methods adopted and the design of the 'filters':

1. Principally, I feel as though the body size filter I s particular is perhaps too harsh…and this is what is driving (what is – at least – presented as) the principal results. One of the things I like about the simulations framework employed by Darroch, Fraser and others is that it dives more deeply into the range of different taphonomic windows offered by the fossil record, and experiments with things like lagerstätten and microsites (in particular things like raptor regurgitites), which preserve small vertebrates in large numbers. After all, vertebrate paleontologists have long been cognisant of the fact that both: a) body size distributions in most mammal clades are right-skewed; and, b) that biases against the preservation of these small-bodied taxa may be a problem when it comes to reconstructing evolutionary history. However, thanks to these more unusual preservational windows we actually have access to an extensive fossil record of small mammals…and the fact that current ancestral state reconstructions suggest a small, rodent-sized insectivore at the base of the placental tree (see the 2013 O'Leary paper in Science) would seem to directly contradict the inferences raised by this study. To address this point, I think it would be useful if the authors could compare the body-size distributions that emerge from the application of their filters, with the known body size distributions from the fossil record…maybe as a whole, or taken from one specific timeslice. This would provide some sense for whether the filters being applied by the authors approximate the fossil record (and thus the results of the analysis are directly applicable), or whether something important is being missed. Without this proof-of-concept, I don't think the principal finding in abstraction ('eliminating small-bodied mammals from the fossil record prevents us from understanding evolution') is a particularly new or useful.

I have listed some other moderate- to minor points below, which should be relatively easy to address.

2. "riche" (line 27) should be 'rich'.

3. "inherently incomplete and non-uniform across space, time, environments, and taxa" (line 27); two key papers that deserve

to be cited here are:

Plotnick R. 2017 Recurrent hierarchical patterns and the fractal distribution of fossil localities. Geology 45, 295–298. (doi:10.1130/G38828.1)

Noto, C. R. 2010. Hierarchical control of terrestrial vertebrate taphonomy over space and time: discussion of mechanisms and implications for vertebrate paleobiology. In P. A. Allison, and D. J. Bottjer, eds. Taphonomy: process and bias through time. Topics in Geobiology 32:287–336. Springer, Dordrecht, Netherlands

4. “simulations suggest that the fossil record might preserve the signature of past biogeographic patterns” (line 41); the Darroch et al. 2018 and 2020 papers in Paleobiology and AmNat respectively probably deserve to be cited here.

5. “Mammals offer a suitable biological model to address this question. They are…one of the best studied” (line 46); another potential advantage, is that they have also been used as a study group by the vast majority of other simulation-based studies examining similar questions.

6. “…we simulate which current mammal species would become part of a future fossil record, according to geological (sediment availability)” (line 55); I actually like the use of the Borker et al. terrestrial sediments compilation here, however, I think some extra information could be given here. For example, are water bodies included in this compilation? (Vertebrate fossil preservation typically occurs in aquatic environments, due to the potential for burial – see the Darroch et al., 2018 paper). No proxy for future fossil formation will ever be perfect, but the reader should have some insights into the pros/cons of this one.

7. “We hypothesise that geographic driven biases would have a lower impact than biological and taxonomic driven biases” (line 67); to what extent are these biases correlated? For example, we know that things like range size, body size etc. are not randomly distributed in space…but rather describe some large-scale patterns driven by physiology, geography etc. To what extent might you expect the filters to be linker or inter-dependent at some scale?

8. “To define these, we extracted all fossil records for mammals from the Paleobiology Database (PBDB)” (line 115); some more information is perhaps needed here…were all records for the entire Cenozoic extracted, if – or so – why the entire Era, rather than one specific Period or Stage? If the idea is to understand to what extent a future paleontologist may be able to reconstruct macroevolutionary patterns, then I would think that one timeslice would be more appropriate…and aggregating localities through tens of millions of years like this is potentially an explanation for why these filters do not have a big impact in the final results.

9. “From our baseline phylogeny of 3,710 mammal species, the number of retained species per filter decayed from 3,294 after sediments to a minimum of 47 after taxonomy weighted in the consecutive approach and to 910 after body-size in the independent approach, under the lowest (25) preservation rate scenario” (line 210); as with comment #1, it would be interesting to compare these numbers with the numbers of species known from individual Stages from the Cenozoic (or potentially, North American Land Mammal Ages).

10. “punctual” (line 223); perhaps a typo here? I’m not sure what ‘punctual’ refers to in this context.

11. “fitlers” (line 497); should be ‘filters’.

(Remarks on code availability)
N/A

Reviewer #2

(Remarks to the Author)
I'm very pleased to read about these innovative means of examining how potential bias in the fossil record influences how we understand two key aspects of trait evolution and biodiversity: body size and diet. However, the text implies something quite different than what the data and methods actually deliver. In pointing this out I do not intend to minimize the significance of the authors' work. However, after the title & abstract I was expecting to find results concerning "reconstructions of evolutionary history" and "extent to which 'true' evolutionary scenarios can be recovered from fragmentary fossil data". In fact, the authors do not investigate phylogeny reconstruction per se, and their investigation is limited to how fossil bias may impact two fairly limited aspects of character evolution: body size and diet. This is not a terminal flaw, but does require that you rephrase your title, abstract & text in general so as not to imply relevance to conclusions beyond what your methods & data permit.

Specific comments are as follows:

Fossils are obviously important but certainly not "the only empirical evidence of how life evolved on Earth". Pseudogenes. Genetic signatures of retroviruses. A gymnosperm transcription factor functional in a flowering plant. Chromosomal fusions evident between close relatives. Phylogenetic bracketing and ancestral state reconstruction based on extant lineages. All qualify as "empirical". Please get rid of the hyperbole.

line 11: why the quotes around "future" and "true"? Are these terms not what you really mean?

line 27: "riche archive" ?

Every occurrence of "e.g." and "i.e." should be followed by a comma.

The phrase "but see" & citations on line 44 makes the previous claim "impact of fossil record biases on phylogenetic inferences beyond diversification rates has received less attention" rhetorical fluff. This topic has been a regular focus of investigation by various groups for decades. Besides the papers cited here, have a look at these (among others):
- Smith, A. B. 1998. Mol. Phylogenet. Evol. 9:437–447.
- Asher RJ, Hofreiter M. 2006. Systematic Biology 55(2):181-194.
- Lee MS, Camens AB. 2009. Journal of Evolutionary Biology. 22(11):2243-57.
- Wiens et al. 2010. Syst. Biol. 59(6):674–688, 2010
- Pattinson DJ, Thompson RS, Piotrowski A, Asher RJ. 2015. Systematic Biology 64(2):169-186.
- Beck RMD, Baillie C. 2018. Proc. R. Soc. B 285:20181632.
- Asher RJ, Smith MR, Rankin A, Emry RJ. 2019. R. Soc. open sci. 6: 190387.
- Yuan H, Dickson ED, Martinez Q, Arnold P, Asher RJ. 2024. Proceedings of the Royal Society B.

Moreover, I don't think this paper actually tests "phylogenetic inferences" insofar as "inference" means testing patterns of phylogenetic relatedness. Rather, as per the title, this paper tests "trait evolution and diversification history", and as the reader discovers buried in Methods, this "history" is restricted to just two things: body size and diet. The authors do not test other characters or the ability of fossilizable data to accurately reconstruct phylogenetic affinities. (See further comments below, e.g., line 139.)

line 67: "geographicALLY driven biases... taxonomicALLY driven..."

line 78: explain what polygons have to do with maps.

line 84: 0.5 degrees LATITUDE, right?

line 139: I sense a difference in academic tradition here from what I'm used to (not in itself a bad thing). When I read "phylogenetic inferences" I understand in the first instance patterns of relatedness, i.e., topology bifurcations showing which terminals (= tips) share close vs. distant common ancestry. But the authors here are not inferring phylogeny, i.e., reconstructing patterns of bifurcation. Rather they're assuming the phylogeny of Alvarez-Carreterro et al. 2022 and investigating "tip-diversification rates" -- which I guess means a combination of branch lengths plus character change -- and trait evolution (body mass and diet) along the mammal tree" -- which I guess means mapping character change on a given topology.
So I'd get rid of the phrase "phylogenetic inference" and write something like
"Our aim was to evaluate the impact of incomplete and biased sampling on the inferred evolution of two characters ..."
Relatedly, you might underscore that, if I've understood correctly, every "tree" (= topology) is compatible with the branching pattern shown by Alvarez-Carreterro et al. By the way, that paper showed one topology in its main text (2022: fig. 3) with illegible tips. Please add reference to the specific figure, probably suppdata, showing the phylogeny you used. The "trees" you mean are those in which certain tips are missing depending on your sampling regime for body size, habitat, range size, etc., correct?

Please avoid acronyms like the plague & write out what "DR" (etc.) means. Same goes for "NRI" a few lines later. For this and every other acronym, please use instead a concise phrase with real words (e.g., "net-relatedness"). For example, if you replace "BM" with "Brownian Motion" you will help your readers to avoid a momentary association of your text with Bowel Movements.

The paragraph starting on line 165 is impenetrable to me. The meaning of "phylogenetic signal of the traits" might mean the ability of those traits to accurately reconstruct phylogenetic branching patterns. Yet paleontologists trying to do so would use a matrix of hundreds of fossilizable characters, largely bones & teeth, not two characters consisting of body mass and diet. It's true that diet will have at least some anatomical consequences for, say, dental morphology. However, you should still avoid giving readers the impression that you're testing how the fossilization filter affects phylogeny reconstruction, something most readers would infer from your abstract: "the extent to which 'true' evolutionary scenarios can be recovered from fragmentary fossil data"

Similarly, you write about "phylogenetic signal" repeatedly in your results (e.g., from line 251), but your meaning is actually much more narrowly circumscribed around two traits: body mass and diet. You should rephrase all of your Results section so as to make clear that you're not actually discussing "Phylogenetic signal [or distribution] of continuous [or discrete] traits" generally, but something like "Phylogenetic signal/distribution of body mass/diet" (as per some of your section headings later on).

line 306 (and elsewhere, including your suppdata text & spreadsheet): "Eulipotyphla" is not a valid Linnean term for any mammalian clade and should be replaced with Lipotyphla (see Asher & Helgen 2011 BMC Evol Biol).

line 316 (and elsewhere): when you use jargon like "kappa model" could you add a phrase or sentence to explain more clearly in plain English what this means.

line 348 "faunivore" is a noun. You need the adjective "faunivorous" here.

line 370: As noted above, your study does not illustrate how "non-uniformity of the fossil record ... can affect phylogenetic inferences on lineage evolution", but rather character state reconstructions of diet and body size. You've not tested anything about "phylogenetic inference" as that phrase is commonly understood, i.e., reconstruction of evolutionary branching events. Furthermore, your text in that same sentence, "such as body mass and diet", implies that you tested other such characters, but as far as I can tell you have not.

line 484: "rather suggested" is not grammatically correct.

Again, on line 515 you refer to "our phylogenetic analysis" but you've not actually undertaken such an analysis, at least not one that maximizes likelihood or minimizes character change in a dataset implied by one topological branching pattern relative to others. Instead, you've relied on the topology in Alvarez-Carreterro et al. 2022. That's OK, but stop implying your study is more than what it actually is. Indeed, the caveats listed in this paragraph ("that the same backbone phylogeny will always be recovered...") should've been clear from both the Abstract and initial sentences of the Introduction.

I hope you'll have a close look at these two papers (already noted above) when you mention "State-of-the-art methods" on line 539:
- Beck RMD, Baillie C. 2018. Proc. R. Soc. B 285:20181632.
- Asher RJ, Smith MR, Rankin A, Emry RJ. 2019. R. Soc. open sci. 6: 190387.
and this paper, one of several since 2006, that examines the reliability of fossilizable data in phylogeny reconstruction (in particular suppdata fig. S9):
- Yuan H, Dickson ED, Martinez Q, Arnold P, Asher RJ. 2024. Proceedings of the Royal Society B.

line 555: "value of the fossil record is out of the question" means you exclude said "value...". I'd write instead something like "While no one doubts the value of the fossil record..."

(Remarks on code availability)

Reviewer #3

(Remarks to the Author)
Rigorous investigations of how biases in taxon sampling affect macroevolutionary inferences from comparative data are incredibly useful and always welcome, in my opinion. As taxon sampling is generally impacted by a complex array of interacting biases, however, it can be difficult to select which exact biases to investigate in a given study—not to mention how to most effectively simulate datasets with those biases. In this regard, starting with empirical data and focusing on well-known biases of the fossil record (e.g., large-bodied, widespread taxa frequenting environments with a high capacity for fossil formation/exposure)—as the authors do here—is certainly an interesting and worthwhile idea.

I have no doubt that this work will prove useful to many macroevolutionary biologists (especially those working with mammals) trying to gauge how the results of their own research are potentially influenced by biases in taxon sampling. Additionally, I could see the study's overall methodology inspiring future work on this important yet generally understudied topic. In particular, testing both the independent and cumulative effects of multiple biases seems like a practical compromise which manages the "combinatorial explosion" of simulation conditions that occur in these kinds of studies while better reflecting the expected complexity of biases affecting empirical datasets. The use of geological/paleontological databases to quantify/simulate the potential for fossilization/subsequent discovery across the globe is also an interesting idea I have not previously encountered (though this may be more common among paleontological research; I generally work with phylogenies primarily consisting of extant taxa).

My one major concern with this paper is that it is currently framed as an investigation of how biases in the fossil record may affect macroevolutionary inferences, yet the authors simulate "fossil record biases" by removing tips from a phylogeny consisting solely of extant mammal taxa. While the abstract and introduction of the manuscript make it clear that the authors use data on extant taxa to simulate likely biases in a hypothetical future fossil record, I still expected that the study would implement some kind of strategy for specifically simulating how biased sampling of non-contemporaneous, extinct taxa (reflecting the typical distribution of fossil data in empirical phylogenies) affect macroevolutionary inferences. Given the overall framing of the current manuscript, I think there are two key issues with the authors' current approach (or at least their interpretation of some results):

1) There are conceptual issues with claiming that these simulations/analyses faithfully represent inferences based on a future fossil record. Under this scenario, more time will have passed by the time these hypothetical future fossils are discovered, such that the apparent distribution of traits (e.g., body size, diet, range size) and species richness (e.g., affecting apparent patterns of lineage diversification) across clades will be different. The extent to which these differences would matter for macroevolutionary inferences is a complex question in and of itself, as it inevitably depends on the assumed timeframe for fossilization/discovery (I suppose around millions to tens of millions of years reflects the typical timeframe of mammalian fossil records unless one is solely focused on the Pleistocene) as well as the evolutionary dynamics of trait evolution/lineage diversification processes across the mammal phylogeny.

2) Extant taxa occur at a single point in time corresponding to the maximum height of a phylogeny, while fossil taxa are generally distributed across multiple time periods throughout a phylogeny. As such, we generally expect that removal/biased sampling of fossil taxa will have distinct impacts on macroevolutionary inferences from phylogenetic comparative data compared to removal/biased sampling of extant taxa.

Accordingly, I have three alternative suggestions for how the authors might revise their manuscript to better address/acknowledge these issues (generally ordered from most ideal to most practical):

1) Explicitly simulate fossilization and/or macroevolutionary processes some amount of time into the future to better address the questions posed in the introduction of the manuscript or at least gauge how much such processes could impact the overall conclusions of this study. While I think this option would greatly strengthen the manuscript, it would also introduce numerous additional complexities/assumptions to the authors' simulation pipeline and seems potentially impractical/beyond the scope of the current work.

2) Reframe the manuscript as being focused on biases in sampling of extant taxa in comparative studies, rather than fossil data. In this context, the fossilization component instead serves as a kind of foundation for identifying common, relatively universal biases that are likely to affect sampling of extant taxa. Admittedly, the preservation bias simulations would appear less well-motivated in this context, and I do think that these simulations are an interesting and unique aspect of the current manuscript.

3) Keep the current framing largely intact while explicitly acknowledging this important caveat with the study's simulation methods/analysis. I think this would reduce confusion among readers as well as help other researchers better contextualize/qualify the study's overall results/conclusions. Admittedly, I am not a paleontologist or mammalogist, so I may be unaware of some concrete, empirically grounded reasons to suspect that the aforementioned issues are inconsequential for the overall results/conclusions of the study. Nonetheless, I think such reasons need to be explicitly stated if they exist, as this seems highly counterintuitive to me given my understanding of macroevolutionary modeling and the general timeframe of mammalian evolution.

Outside of this one major concern, I think this study presents some unique and innovative strategies for simulating/investigating the impacts of sampling biases on macroevolutionary inferences and contains a lot of useful, generalizable information/insights. I provide some additional, more minor feedback on a line-by-line basis below:

L25-27: Perhaps this is only semantic/philosophical, but I would argue that comparative biology is another major, albeit indirect, source of empirical information on how life evolved on Earth.

L27: "riche" should be "rich"

L42-44: While the authors are correct that the impact of fossil record biases on macroevolutionary inferences (particularly with respect to trait evolution) is understudied, I think it is worth mentioning here that there is relevant previous work on how inclusion of fossil data (e.g., Slater et al 2012, https://onlinelibrary.wiley.com/doi/full/10.1111/j.1558-5646.2012.01723.x; Puttick 2016, https://onlinelibrary.wiley.com/doi/full/10.1111/j.1558-5646.2012.01723.x; Schnitzler et al 2017, https://drive.google.com/file/d/16qoCwyIXOKNhVfQPPeJkCOvQQE2RIkBR/view) as well as biased sampling of extant taxa (e.g., Ackerly 2000, https://onlinelibrary.wiley.com/doi/abs/10.1111/j.0014-3820.2000.tb00694.x; Marcondes 2019, https://pmc.ncbi.nlm.nih.gov/articles/PMC6791351/; Johnson et al 2020, https://onlinelibrary.wiley.com/doi/full/10.1111/geb.13185) affect trait evolution inferences from phylogenetic comparative data.

L181-182: Brownian Motion refers to a specific stochastic process which is only applicable to continuously varying quantities—it does not apply to discrete trait evolution models, which are instead based on continuous time Markov Chains (CTMCs). I think the most clear way to state the authors' methods here is that they fit Brownian Motion models to the continuous trait data and ER/ARD models to the discrete trait data either without or with one of Pagel's three tree transformations. This is because one is essentially still fitting the same basic models to the data even with Pagel's tree transformations in this context—these transformations only serve to squash/stretch the branches of the phylogeny to further improve the model's fit to the data.

L188-189: I think it is worth mentioning here that delta<1 corresponds to the popular "early burst" (EB) model of trait evolution (Harmon et al 2010, https://academic.oup.com/evolut/article-abstract/64/8/2385/6854028?redirectedFrom=fulltext&login=false) while delta>1, at least for ultrametric data (as is the case in the current manuscript), corresponds to the popular "Ornstein Uhlenbeck" (OU) model of trait evolution (at least assuming a single trait optimum applies to the entire phylogeny; Blomberg et al 2003, https://academic.oup.com/evolut/article-abstract/57/4/717/6756141?redirectedFrom=fulltext&login=false). While I think Pagel's tree transformations are conceptually and practically useful, they have fallen out of favor compared to the increasingly ubiquitous Brownian Motion/EB/OU model selection framework in my experience. Making this explicit connection will enable more readers to easily relate the results of this paper to their own research. Notably, however, revising the study to involve non-ultrametric phylogenies with extinct taxa will render delta>1 no longer equivalent an OU model. In that case, delta>1 is best thought of as modeling a "late burst" in contrast to an "early burst".

L263: This is only a minor point, but I do wonder to what extent the overdispersion of medium sizes may be a simple

consequence of the ordinal nature of a discretized measurement of body mass. In other words, you may inevitably expect medium sizes to be phylogenetically overdispersed because small-bodied lineages generally have to pass through medium-bodied phase to become large-bodied and vice versa. To that end, I also think it would be interesting (albeit not strictly necessary) to explore how the results of the discrete trait evolution analyses differ if one fits ordinal models of discrete trait evolution (i.e., forbidding direct transitions from small to large-bodied to states; these are termed "meristic" models in geiger) in addition to ER/ARD models (the authors could also do something similar for herbivory/omnivory/faunivory). Unfortunately, I cannot think of a straightforward way to incorporate the natural "order" of states into metrics of phylogenetic under/overdispersion. Perhaps seeing how the NRI results change when treating body size as a binary character could shed some light on this issue.

L303-304: "increased respect" should be "increased with respect"

L451: "Random evolution" is a somewhat vague interpretation of Brownian Motion-like evolutionary processes. More specifically, Brownian Motion (at least with no tree transformation) corresponds to what would be expected under pure drift/neutral dynamics OR selection with randomly varying direction/strength across time/lineages.

I sincerely hope the authors find this feedback useful!

(Remarks on code availability)
I have not investigated the authors' code in detail, but skimmed through some of it. In general, everything looked organized and straightforward to understand/reproduce. Additionally, the authors do a thorough job of describing their methods in the paper itself.

Version 1:

Reviewer comments:

Reviewer #1

(Remarks to the Author)
I thank the authors for their thoughtful revision and responses to my comments. Although maintain that the body size filter is too harsh – and that the fossil record of small vertebrates is better than is being modeled – I agree that the broadest finding (that aspects of evolutionary history are likely erased by the fossilization process and taphonomic biases) is well-made, and…if not strictly novel in a paleontological sense…is nicely demonstrated in these experiments. I only have a few additional comments that the authors may wish to take on board, or ignore as they see fit.

1. Following from my main point above, it might be worth emphasizing somewhere in the discussion that efforts to reconstruct the small vertebrate record – specifically by focusing on unusual, but widespread taphonomic windows like regurgitate deposits – should arguably be a bigger priority in both paleontology and evolutionary biology. I particularly like the 2010 paper by Rebecca Terry in Proceedings B, which illustrates not only that these deposits preserve the small mammal record extremely well, but also that they're more widespread that commonly appreciated. In the scenario the authors are considering…i.e., trying to reconstruct evolutionary history using an imagined fossil record of extant mammals, it's doubtless that lagerstätten, regurgitates, and even cave deposits where many common taphonomic biases do not operate, would be used. In turn, the author's findings illustrate that – if we're hoping to understand actual evolutionary history – devoting more resources to studying these deposits is crucial.
2. A related point, is that taphonomic processes are not exactly operating in the present day as they have in the past, and this might be worth thinking about (again, in context of the exercise the authors are walking through: modeling a future fossil record). It's probably only worth a brief mention, but Plotnick and Koy (2020) in Anthropocene ("The Anthropocene fossil record of terrestrial mammals") has some valuable insights.

(Remarks on code availability)

Reviewer #3

(Remarks to the Author)
The authors have done a fantastic and thorough job addressing reviewer feedback, demonstrating careful attention to detail and thoughtful consideration of each comment. Even in cases where the authors did not substantially alter their work in response to feedback (e.g., not adding a temporal component to their simulations, not treating diet/discretized body mass as ordinal variables), they nonetheless provided clear justifications for their decisions.

Overall, I think this is a quality and insightful contribution to the literature on how taxon sampling biases affect macroevolutionary inferences. Further, the heavy reliance on empirical data and trends in the authors' simulation pipeline appears rather novel and interesting (though there could be some literature I am unaware of). Even with the caveats of the study (which I think the authors do a much better job of acknowledging in their revised manuscript!), I agree with the authors that their work provides an excellent set of "baseline" results which can be further interrogated going forward. In any case, I think this paper has value not only in the results it directly provides, but also the simulation strategies it might inspire others to do to gain further clarity on these topics.

I do not have any remaining major criticisms of the paper, though I did find the wording of some sentences vague or unclear, which I detail below (line numbers based on clean copy):

L36: This is super minor, but I found myself getting tripped up by "while" in this sentence--"though" might fit better?

L41-42: I might suggest rewording as "...with a general consensus that combining data from fossil and living taxa increases phylogenetic accuracy..."

L46: I think the first sentence of this paragraph is a little vague (what question exactly, and what does your approach differ from?). I would simply say something along the lines "Here, we use data on extant taxa to explicitly simulate the effects of fossil biases on macroevolutionary inferences..."

L54: "distribution" should be plural here, I believe.

L67-68: Apologies if I'm getting nitpicky here, but I'm not sure "conservative" is quite the right word here--I think a clearer way to explain your point would be to simply say you're trying to isolate the effects of fossil records biases on macroevolutionary inferences. While it's unrealistic to assume the phylogenetic reconstruction would be identical, re-estimating the phylogeny would not only be impractical for the scope of this study, but also leave it unclear whether downstream effects on macroevolutionary inferences were driven primarily by inaccurate phylogenetic reconstruction (a much more well-studied topic) or fossil record biases.

Ah, I see you elaborate on this point more in the discussion! Well, I think it works in that context with the subsequent sentences--I might suggest just making the point a bit more explicit in the introduction.

Also, some sources that might strengthen some of your points on avoiding phylogenetic reconstruction/lacking temporal realism: https://doi.org/10.1093/sysbio/syq098, https://doi.org/10.1002/ecy.2788

To be clear, I think the caveats here are important to explicitly state and talk about, as I still think they could have major effects on the results (a point I'm sure you all agree with in any case!). Further, these papers only cover rather narrow aspects of macroevolutionary inference (regression, phylogenetic diversity, phylogenetic signal...). That being said, I nonetheless think these citations could provide readers with a bit more confidence in the "gist" of your results by showing how some common phylogenetic comparative analyses appear rather robust to error in tree topology/branch length estimates (at least to a degree).

L76: "...how these traits are distributed across the phylogeny..." is the more conventional phrasing, in my experience.

L204: "...it was best fit by..." is more common/clear phrasing than "...it best adjusted to..." in my experience.

L210: "none tree transformation" should be "no tree transformation" here, right?

L427-428: I would instead write "...instead of focusing on uncertainty around individual estimates..." as this would apply to both model selection and parameter uncertainty. Confidence intervals around values makes it sounds like you're only referring to parameter uncertainty.

(Remarks on code availability)

Open Access This Peer Review File is licensed under a Creative Commons Attribution 4.0 International License, which permits use, sharing, adaptation, distribution and reproduction in any medium or format, as long as you give appropriate credit to the original author(s) and the source, provide a link to the Creative Commons license, and indicate if changes were made.
In cases where reviewers are anonymous, credit should be given to 'Anonymous Referee' and the source.
The images or other third party material in this Peer Review File are included in the article's Creative Commons license, unless indicated otherwise in a credit line to the material. If material is not included in the article's Creative Commons license and your intended use is not permitted by statutory regulation or exceeds the permitted use, you will need to obtain permission directly from the copyright holder.
To view a copy of this license, visit https://creativecommons.org/licenses/by/4.0/

RESPONSE TO REVIEWER COMMENTS

We thank the reviewers for their time and effort in revising our submission. We really appreciate the careful reading of the manuscript and detailed comments, which have helped us to improve the clarity and framing of our work. Please find below our point-by-point response (**in bold**) to the reviewers' comments, where line numbers correspond to the revised version with tracked changes (unless otherwise stated).

Reviewer #1 (Remarks to the Author):

This paper presents a simulations-based study aimed at understanding the impacts of various geological, taphonomic, and taxon-based 'filters' that affect the preservation potential of different terrestrial mammals, on reconstructing some key macroevolutionary 'traits'…including 'modes' of evolution, diversification rates, and the ancestral body mass and diet state. The authors consider a range of both biotic and abiotic factors; biotic factors include things like body size and range size (the idea being that small-bodied and -ranged taxa tend to have lower preservation probabilities), while abiotic factors include whether or not potential fossil 'localities' overlap with the species range – with localities (i.e., potentially fossiliferous rock) defined on the basis of unconsolidated sediments – and whether or not we're likely to find them (using the current locations of mammalian fossil sites downloaded from the PBDB – reflecting the fact that there is a geographic bias in paleontological exploration and sampling efforts). Finally, the authors impose a 'taxonomic' filter, tossing out species (essentially – counting them as 'not found') either on the basis on having low numbers of observations in the fossil record, or belonging to poorly-sampled families…the logic being, that paleontologists tend to work on some clades more than others. The results of the analysis show– perhaps not surprisingly – that the biological filters skew the distributions of body mass and diet, thus introducing errors into inference of ancestral state…while the abiotic factors tend to have much lesser impacts.

There are a growing number of simulations-based studies like this one, and the approach taken – one that aims to reconstruct evolutionary traits, rather than biogeographic patterns or extinction signal – is welcome and novel.

Response: We thank the reviewer for the positive feedback on our study.

That said, I have some reservations about the paper that stem from some of the methods adopted and the design of the 'filters':

1. Principally, I feel as though the body size filter I s particular is perhaps too harsh…and this is what is driving (what is – at least – presented as) the principal results. One of the things I like about the simulations framework employed by Darroch, Fraser and others is that it dives more deeply into the range of different taphonomic windows offered by the fossil record, and experiments with things like lagerstätten and microsites (in particular things like raptor regurgitites), which preserve small vertebrates in large numbers. After all, vertebrate paleontologists have long been cognisant of the fact that both: a) body size distributions in most mammal clades are right-skewed; and, b) that biases against the preservation of these small-bodied taxa may be a problem when it comes to reconstructing evolutionary history. However, thanks to these more unusual preservational windows we actually have access to an extensive fossil record of small mammals…and the fact that current ancestral state reconstructions suggest a small, rodent-sized insectivore at the base of the placental tree (see the 2013 O'Leary paper in Science) would seem to directly contradict the inferences raised by this study. To address this point, I think it would be useful if the authors could compare the body-size distributions that emerge from the application of their filters, with the known body size distributions from the fossil record…maybe as a whole, or taken from one specific timeslice. This would provide some sense for whether the filters being applied by the authors approximate the fossil record (and thus the results of the analysis are directly applicable), or whether something important is being missed. Without this proof-of-concept, I don't think the principal finding in abstraction ('eliminating small-bodied mammals from the fossil record prevents us from understanding evolution') is a particularly new or useful.

Response: We thank the reviewer for this thoughtful and constructive comment, which touches on an important conceptual issue: how realistically our simulated filters approximate the fossil record, and so how the resulting inferences should be interpreted.

We agree with the reviewer that the body-size filter has a strong effect on the inferred evolutionary patterns. This is expected, as we note in the discussion (lines 316–319). Importantly, however, the body size filter does not imply that small mammals are not preserved but that they are less likely to be so, and we further implement three filter levels (25%, 50%, and 75% preservation rates) to account for a differential impact. This approach is consistent with previous simulation-based studies that treat preservation as heterogeneous and incomplete rather than binary (e.g., Fraser 2017, https://doi.org/10.1017/pab.2017.2; Darroch et al. 2021, https://doi.org/10.1098/rspb.2020.2927). Furthermore, no "true" preservation rates ultimately exist as they vary with a plethora of processes, settings, and entities. Applying multiple filter levels allows us to capture the impact of a range of preservation rates.

We fully acknowledge that exceptional preservational windows such as lagerstätten and microsites (including "raptor regurgitites") provide critical access to small-bodied mammals. However, these windows are geographically restricted, taxonomically selective, and temporally discontinuous. For instance, the study by Darroch et al. 2021, where lagerstätten are simulated, is restricted to North American mammals mostly from the Rancholabrean North American Land Mammal Age (220–11 thousand years ago). The same reasoning applies to other events such as raptor regurgitates, which are further limited to specific organisms (both predators and preys).

In terms of ancestral body size reconstruction, we want to note that our results do not contradict what O'Leary et al. propose (based on data from fossils and living taxa), which is a small and insectivore (faunivore in our classification) placental ancestor. Although we do not look into the placental ancestor, many of our simulated fossil samples would lead to such an inference for the mammalian ancestor (small and faunivore), especially under the 50% preservation rate scenario. That a sample is biased against small-bodied species does not necessarily mean that a small insectivore ancestor cannot be inferred, we indeed show that it depends on the model used and the particular composition of the sample analysed (see Fig. 5 and Supplementary Fig. 16).

The reviewer suggests comparing simulated body-size distributions to empirical fossil distributions as a validation step. While we appreciate the motivation behind this suggestion, we caution that such comparison is not straightforward. Our simulation begins from present-day mammal richness, whereas the empirical fossil record derives from an unknown and time-varying baseline richness. As shown by recent work (e.g., Lindholm & Close 2025, https://doi.org/10.1098/rspb.2025.1780), mammalian body-size distributions in the fossil record fluctuate substantially across the Cenozoic, making any single distribution an unreliable benchmark.

We remark that our purpose was to simulate biases known to exist in the current fossil record, not to mimic the process giving rise to currently collected fossils. Still, the percentage of species (total and by major orders) retained under the 25% and 50% preservation rate scenarios are similar to the completeness estimated for Miocene and Pleistocene mammal fossil records in previous works, as indicated in the discussion (lines 306–313). Furthermore, we acknowledge that other sources of bias than the ones we implemented here play a role in shaping current fossil representation (e.g., lines 283–286, 412–416).

Our goal was therefore not to reproduce the fossil record as observed, but to explore how known biases, applied to a complete community, can distort downstream evolutionary inferences. Overall, the take-home message we intend to pass with our work is not about the impact of fossil record biases on the inferred evolution of mammals in particular but of any extinct community in general.

We use mammals as an example to illustrate the extent to which fossil record biases can provide a distorted picture of the composition of past communities (because some taxa are preferentially lost) and how this impacts derived evolutionary estimates. For instance, we show that major mammal orders, such as Chiroptera, could leave no trace at all in a potential future fossil record. From any given extinct community, we cannot really know which taxa have been lost or what their proportion was in that community, so the impact on evolutionary estimates cannot be predicted either. Therefore, that limitation should always be kept in mind when drawing conclusions from the fossil record. We have tried to clarify our message in this revised version, both in the introduction and discussion sections.

I have listed some other moderate- to minor points below, which should be relatively easy to address.

2. “riche” (line 27) should be ‘rich’.

Response: We have corrected the typo.

3. “inherently incomplete and non-uniform across space, time, environments, and taxa” (line 27); two key papers that deserve to be cited here are:

Plotnick R. 2017 Recurrent hierarchical patterns and the fractal distribution of fossil localities. Geology 45, 295–298. (doi:10.1130/G38828.1)

Noto, C. R. 2010. Hierarchical control of terrestrial vertebrate taphonomy over space and time: discussion of mechanisms and implications for vertebrate paleobiology. In P. A. Allison, and D. J. Bottjer, eds. Taphonomy: process and bias through time. Topics in Geobiology 32:287–336. Springer, Dordrecht, Netherlands

Response: We thank the reviewer for the references, which we have now included in the text (line 31).

4. “simulations suggest that the fossil record might preserve the signature of past biogeographic patterns” (line 41); the Darroch et al. 2018 and 2020 papers in Paleobiology and AmNat respectively probably deserve to be cited here.

Response: We thank the reviewer for the references, which we have now included in the text (lines 47–48).

5. "Mammals offer a suitable biological model to address this question. They are…one of the best studied" (line 46); another potential advantage, is that they have also been used as a study group by the vast majority of other simulation-based studies examining similar questions.

Response: We thank the reviewer for noting this further advantage of the use of mammals as an example. We have added this point to the text (lines 67–68), which now reads:

"…, and they have been the subject of previous investigations on fossil record biases [e.g., 24,28,35,52,53]."

6. "…we simulate which current mammal species would become part of a future fossil record, according to geological (sediment availability)" (line 55); I actually like the use of the Borker et al. terrestrial sediments compilation here, however, I think some extra information could be given here. For example, are water bodies included in this compilation? (Vertebrate fossil preservation typically occurs in aquatic environments, due to the potential for burial – see the Darroch et al., 2018 paper). No proxy for future fossil formation will ever be perfect, but the reader should have some insights into the pros/cons of this one.

Response: We have added those details on the sedimentary classes we used to the methods section (lines 533–537), which now reads:

"To simulate this, we downloaded a global map of unconsolidated sediments currently exposed on the land surface [89], consisting of 39 sedimentary classes. Among them, we excluded 'ice and glaciers', 'proglacial', 'till', and 'glacial, undifferentiated' because of their extremely low preservation potential, while all water bodies were retained."

7. "We hypothesise that geographic driven biases would have a lower impact than biological and taxonomic driven biases" (line 67); to what extent are these biases correlated? For example, we know that things like range size, body size etc. are not randomly distributed in space…but rather describe some large-scale patterns driven by physiology, geography etc. To what extent might you expect the filters to be linker or inter-dependent at some scale?

Response: We agree with the reviewer that global biodiversity patterns are modulated by geography, as the observed increase of species' range size and body size with latitude, for example, but these patterns apply to all groups/species. At the species level, we further agree that the filters we use are not completely independent from each other. Apart from the general link between

range and body size, the taxonomic filters, for instance, are influenced by all remaining ones. The frequency of a given family in PBDB is not only the result of human research capacity/interests but also of the conditions that made possible their preservation (sediments, range size, body size) and discovery (sampling spatial). These are fair points, and we have now added more details in this respect to the methods section (lines 552–559).

8. "To define these, we extracted all fossil records for mammals from the Paleobiology Database (PBDB)" (line 115); some more information is perhaps needed here…were all records for the entire Cenozoic extracted, if – or so – why the entire Era, rather than one specific Period or Stage? If the idea is to understand to what extent a future paleontologist may be able to reconstruct macroevolutionary patterns, then I would think that one timeslice would be more appropriate…and aggregating localities through tens of millions of years like this is potentially an explanation for why these filters do not have a big impact in the final results.

Response: We have used all mammal records from all geological periods from PBDB. We have added this clarification, "from all geological periods", to the text (line 544).

We used all mammal fossil records because our aim was to capture the biased/unequal coverage that humans make of geographic regions and taxonomic groups when they study fossil sites. Because we are not interested in any stage-specific dynamics, we start the simulation by applying the sediments filter, considering "all" sedimentary places currently exposed without predicting any future availability of fossiliferous rocks. Otherwise, we will need to set a specific "future" time period, with additional assumptions on future land configurations or human settlement areas, among others. For that reason, we decided to keep all the information we have nowadays. We selected currently exposed sedimentary areas and all available data on sampling effort (using all PBDB occurrences as a proxy), deliberately avoiding limiting the study to a particular time in the future or in the past. Nonetheless, in the study we use as reference for the simulation (Galván et al. 2025, https://doi.org/10.1111/geb.70075), the authors took this concern into account, using only PBDB Quaternary fossil records (instead of all) as baseline data too. Results were similar between both approaches, since they rendered a similar distribution of fossil records in spatial and taxonomic terms, which supports our decision on using the whole mammal fossil record. Further constraining this filter would not change the overall conclusions of our work.

9. "From our baseline phylogeny of 3,710 mammal species, the number of retained species per filter decayed from 3,294 after sediments to a minimum of 47 after taxonomy weighted in the

consecutive approach and to 910 after body-size in the independent approach, under the lowest (25) preservation rate scenario” (line 210); as with comment #1, it would be interesting to compare these numbers with the numbers of species known from individual Stages from the Cenozoic (or potentially, North American Land Mammal Ages).

Response: In line with our response to comment #1, we consider that such comparison would not be informative because the source communities for our simulation and for currently collected fossils are different. Our baseline is the set of present-day land mammal species, but the source community of currently collected mammal fossils (from the whole Cenozoic or from individual stages) is unknown. The real composition of past mammal communities, in terms of total number of species and/or group proportions, cannot be reliably estimated, making such comparison uninformative.

10. “punctual” (line 223); perhaps a typo here? I’m not sure what ‘punctual’ refers to in this context.

Response: We were referring to the absence of rodents in a few samples. For clarity, we have replaced “punctual” by “sporadic”.

11. “fitlers” (line 497); should be ‘filters’.

Response: We have corrected the typo.

Reviewer #1 (Remarks on code availability):

N/A

Reviewer #2 (Remarks to the Author):

I'm very pleased to read about these innovative means of examining how potential bias in the fossil record influences how we understand two key aspects of trait evolution and biodiversity: body size and diet.

Response: We thank the reviewer for the positive words on our approach.

However, the text implies something quite different than what the data and methods actually deliver. In pointing this out I do not intend to minimize the significance of the authors' work. However, after the title & abstract I was expecting to find results concerning "reconstructions of evolutionary history" and "extent to which 'true' evolutionary scenarios can be recovered from fragmentary fossil data". In fact, the authors do not investigate phylogeny reconstruction per se, and their investigation is limited to how fossil bias may impact two fairly limited aspects of character evolution: body size and diet. In addition, they base their analysis on a phylogeny including all major crown mammalian high-level clades (Alvarez et al. 2022), but have measured the two aspects of character change in just six of those clades (as per their lines 194-195). This is not a terminal flaw, but does require that you rephrase your title, abstract & text in general so as not to imply relevance to conclusions beyond what your methods & data permit.

Response: We thank the reviewer for pointing out our imprecise use of some terms throughout the manuscript, which we have tried to correct in this revised version.

We acknowledge that we used evolutionary history/scenarios in a broad sense. Certainly, the reconstruction of a phylogeny, of the branching patterns, is a first fundamental step, but history/scenarios also include the changes that happened along those branches and how/when/where they happened.

We would like to note that we did not claim to analyse the effect of fossil record biases on the reconstruction of the mammal phylogeny. In the original/submitted abstract, we stated that the filtered-out species were "…systematically removed from the baseline phylogeny before analyses of trait evolution using phylogenetic comparative methods…" (lines 16–18). We also indicated our focus on trait evolution in the introduction: "We focus on the evolution of body mass and diet along the mammal phylogeny, two fundamental species' traits that can be generally obtained from fossil remains. We apply standard comparative phylogenetic methods to estimate how these traits distribute in the phylogeny, their mode of evolution, and their ancestral states across simulated fossil samples with respect to baseline data." (lines 88–92). In the methods section, which was right after the introduction in the submitted version and has been moved to the end in the revised version, we also tried to be clear about our methodology, to help interpret our findings. We specified that we just pruned the filtered-out species from the baseline phylogeny (lines 575–578) before proceeding with the analyses.

We analysed all mammals together throughout and, for ancestral state reconstruction, we also reported results for each of the six most abundant clades, those with more than 200 species in the

initial dataset (lines 630–633), which cover present-day land mammal diversity in terms of body size and diet while accounting for 89% of the initial species.

Following the reviewer's comments, we have made changes in the title, abstract, and main text.

Specific comments are as follows:

Fossils are obviously important but certainly not "the only empirical evidence of how life evolved on Earth". Pseudogenes. Genetic signatures of retroviruses. A gymnosperm transcription factor functional in a flowering plant. Chromosomal fusions evident between close relatives. Phylogenetic bracketing and ancestral state reconstruction based on extant lineages. All qualify as "empirical". Please get rid of the hyperbole.

Response: The reviewer is right; we have replaced "the only" by "primary".

line 11: why the quotes around "future" and "true"? Are these terms not what you really mean?

Response: We used the quotes because we refer to unknown parameters. We simulate future fossils, but that future is hypothetical. The same applies to evolutionary inferences, they are not free of uncertainties even when derived from extant species, so we do not know the truth. For simplicity, we have removed the quotes and replaced "true" by "real".

line 27: "riche archive" ?

Response: We have corrected the typo, deleting the "e" from "riche".

Every occurrence of "e.g." and "i.e." should be followed by a comma.

Response: Though both styles (with or without a comma) are accepted in British English, we have changed it throughout the manuscript following the reviewer's recommendation.

The phrase "but see" & citations on line 44 makes the previous claim "impact of fossil record biases on phylogenetic inferences beyond diversification rates has received less attention" rhetorical fluff. This topic has been a regular focus of investigation by various groups for decades. Besides the papers cited here, have a look at these (among others):

- Smith, A. B. 1998. Mol. Phylogenet. Evol. 9:437–447.
- Asher RJ, Hofreiter M. 2006. Systematic Biology 55(2):181-194.
- Lee MS, Camens AB. 2009. Journal of Evolutionary Biology. 22(11):2243-57.
- Wiens et al. 2010. Syst. Biol. 59(6):674–688, 2010
- Pattinson DJ, Thompson RS, Piotrowski A, Asher RJ. 2015. Systematic Biology 64(2):169-186.
- Beck RMD, Baillie C. 2018. Proc. R. Soc. B 285:20181632.
- Asher RJ, Smith MR, Rankin A, Emry RJ. 2019. R. Soc. open sci. 6: 190387.
- Yuan H, Dickson ED, Martinez Q, Arnold P, Asher RJ. 2024. Proceedings of the Royal Society B.

Response: We acknowledge our misuse of "phylogenetic inferences", as we were meaning inferences derived from/based on phylogenies. We thank the reviewer for the references, which focus on the utility of combining morphological and molecular data from extinct and extant species towards increasing phylogenetic accuracy. We have adjusted the introduction following the reviewer's recommendation, including some of those references alongside Asher and Smith 2022. The text now reads (lines 48–51):

"In a phylogenetic framework, fossil sampling has proven to impact tree reconstruction [29–35] and time-calibration of diversification events [36,37], with a general consensus on the benefit of combining data from fossil and living taxa towards increasing phylogenetic accuracy (as reviewed by [38–40])."

Moreover, I don't think this paper actually tests "phylogenetic inferences" insofar as "inference" means testing patterns of phylogenetic relatedness. Rather, as per the title, this paper tests "trait evolution and diversification history", and as the reader discovers buried in Methods, this "history" is restricted to just two things: body size and diet. The authors do not test other characters or the ability of fossilizable data to accurately reconstruct phylogenetic affinities. (See further comments below, e.g., line 139.)

Response: We thank the reviewer for pointing out the misuse of "phylogenetic inferences", as we were meaning inferences derived from/based on phylogenies throughout the manuscript. We have tried to correct that as indicated in previous responses.

line 67: "geographicALLY driven biases... taxonomicALLY driven..."

Response: We have corrected those words.

line 78: explain what polygons have to do with maps.

Response: In GIS, a polygon is a two-dimensional, closed shape defined by pairs of coordinates. Polygons are usually used to represent areas on a map, and they are the provided data type for species' ranges by IUCN. We have added a clarification to the text (lines 501–502):

"... polygons (i.e., map areas) ..."

line 84: 0.5 degrees LATITUDE, right?

Response: We refer to grids of 0.5º latitude and 0.5º longitude. We have clarified it in the text (lines 507–508).

line 139: I sense a difference in academic tradition here from what I'm used to (not in itself a bad thing). When I read "phylogenetic inferences" I understand in the first instance patterns of relatedness, i.e., topology bifurcations showing which terminals (= tips) share close vs. distant common ancestry. But the authors here are not inferring phylogeny, i.e., reconstructing patterns of bifurcation. Rather they're assuming the phylogeny of Alvarez-Carreterro et al. 2022 and investigating "tip-diversification rates" -- which I guess means a combination of branch lengths plus character change -- and trait evolution (body mass and diet) along the mammal tree" -- which I guess means mapping character change on a given topology. So I'd get rid of the phrase "phylogenetic inference" and write something like "Our aim was to evaluate the impact of incomplete and biased sampling on the inferred evolution of two characters ..."

Response: We have modified the heading of the section and its initial sentence. They now read (lines 571–574):

"<u>Phylogenetic comparative methods</u>

Our aim was to evaluate the impact of incomplete and biased sampling, as expected from the fossil record, on evolutionary inferences in a phylogenetic context."

Relatedly, you might underscore that, if I've understood correctly, every "tree" (= topology) is compatible with the branching pattern shown by Alvarez-Carreterro et al. By the way, that paper showed one topology in its main text (2022: fig. 3) with illegible tips. Please add reference to the specific figure, probably suppdata, showing the phylogeny you used. The "trees" you mean are

those in which certain tips are missing depending on your sampling regime for body size, habitat, range size, etc., correct?

Response: That is right; as indicated before, we pruned the species that were filtered-out in each sample from the initial tree. The tree we used is available at https://github.com/sabifo4/mammals_dating/blob/main/02_SeqBayes_S2/03_Generate_final_mammal_tree/4705sp.tree, and is shown in Fig. 3 from Álvarez-Carretero et al. (2022). It is provided as a NEXUS file, which is a standard format to store phylogenetic trees. We have added the link to the text (lines 509–511).

Please avoid acronyms like the plague & write out what "DR" (etc.) means. Same goes for "NRI" a few lines later. For this and every other acronym, please use instead a concise phrase with real words (e.g., "net-relatedness"). For example, if you replace "BM" with "Brownian Motion" you will help your readers to avoid a momentary association of your text with Bowel Movements.

Response: The acronyms were defined the first time they appeared, in the methods section of the submitted version (e.g., NRI on line 174 and BM on line 181 of that version), which was right after the introduction. Because of that, we did not spell acronyms out in the posterior sections. Nonetheless, we have now added the meaning of acronyms to the first time they are used in each section (results, discussion, and methods).

The paragraph starting on line 165 is impenetrable to me. The meaning of "phylogenetic signal of the traits" might mean the ability of those traits to accurately reconstruct phylogenetic branching patterns. Yet paleontologists trying to do so would use a matrix of hundreds of fossilizable characters, largely bones & teeth, not two characters consisting of body mass and diet. It's true that diet will have at least some anatomical consequences for, say, dental morphology. However, you should still avoid giving readers the impression that you're testing how the fossilization filter affects phylogeny reconstruction, something most readers would infer from your abstract: "the extent to which 'true' evolutionary scenarios can be recovered from fragmentary fossil data"

Response: The reviewer is right; the phylogenetic signal of a trait can be defined as the power of that trait to reconstruct phylogenetic relationships. However, we use this term in a comparative phylogenetic context, where it is also defined “as the statistical nonindependence among species trait values due to their phylogenetic relatedness” (Revell et al. 2008,

https://doi.org/10.1080/10635150802302427). We have clarified that in the text (lines 599–601), which now reads:

"We first evaluated the phylogenetic signal of the traits, defined as the tendency for closely related species to resemble each other more than less related ones because of shared evolutionary history [93]."

Here, what we are measuring with "phylogenetic signal" is how the traits are distributed along the phylogeny, as indicated in the text. We would like to note, nonetheless, that both definitions of phylogenetic signal go in the same direction: if trait values are more similar among relatives than among distant taxa, it means that the trait holds information on the branching pattern, and so it can contribute to reconstruct the phylogenetic relationships within the group under study.

Similarly, you write about "phylogenetic signal" repeatedly in your results (e.g., from line 251), but your meaning is actually much more narrowly circumscribed around two traits: body mass and diet. You should rephrase all of your Results section so as to make clear that you're not actually discussing "Phylogenetic signal [or distribution] of continuous [or discrete] traits" generally, but something like "Phylogenetic signal/distribution of body mass/diet" (as per some of your section headings later on).

Response: We have changed the headings following the reviewer's recommendation.

line 306 (and elsewhere, including your suppdata text & spreadsheet): "Eulipotyphla" is not a valid Linnean term for any mammalian clade and should be replaced with Lipotyphla (see Asher & Helgen 2011 BMC Evol Biol).

Response: The term Eulipotyphla was originally coined to avoid confusion with historical uses of Lipotyphla and Insectivora, which have been shown to be non-monophyletic according to molecular data, encompassing the orders Eulipotyphla and Afrosoricida (e.g., Douady and Douzery 2003, https://doi.org/10.1016/S1055-7903(03)00119-2). Since then, both names have continued to be used in the literature under different nomenclatural conventions. For example, Lipotyphla is adopted by Álvarez-Carretero et al. 2022 (https://doi.org/10.1038/s41586-021-04341-1), the source of our baseline tree, whereas Eulipotyphla is used by Upham et al. 2019 (https://doi.org/10.1371/journal.pbio.3000494) in a previous mammal phylogeny. In the current version of the Mammal Diversity Database (MDD, https://www.mammaldiversity.org/), a project led by Upham and funded primarily by the American Society of Mammalogists, it is noted that:

“Asher and Helgen (2010) also recommended using Lipotyphla over Eulipotyphla, but confusion around the association of Lipotyphla with past definitions of the paraphyletic Insectivora assemblage (Woodman 2018) has prompted the MDD to favor Eulipotyphla as the first name used for the current definition of the order”. The term Eulipotyphla is also used by the IUCN (https://www.iucnredlist.org/). This reflects the absence of a universally enforced standard name for this clade, rather than a disagreement about its phylogenetic composition. In line with the reviewer’s request, we have adopted Lipotyphla throughout the revised version. We note, however, that alternative nomenclatural practices remain in active use and that the chosen name does not affect the phylogenetic or analytical results presented here.

line 316 (and elsewhere): when you use jargon like "kappa model" could you add a phrase or sentence to explain more clearly in plain English what this means.

Response: As indicated in the response to the previous comment on acronyms, the models were defined in the methods section of the submitted version (lines 181–189 of that version), before results and discussion. Nonetheless, we have now added the description to the first time they are mentioned in every section of the revised version.

line 348 "faunivore" is a noun. You need the adjective "faunivorous" here.

Response: We have corrected the typo, now it reads: “a faunivore” (line 256).

line 370: As noted above, your study does not illustrate how "non-uniformity of the fossil record ... can affect phylogenetic inferences on lineage evolution", but rather character state reconstructions of diet and body size. You've not tested anything about "phylogenetic inference" as that phrase is commonly understood, i.e., reconstruction of evolutionary branching events. Furthermore, your text in that same sentence, "such as body mass and diet", implies that you tested other such characters, but as far as I can tell you have not.

Response: We have modified the sentence to clarify what we have done. Now it reads (lines 277–279):

“Our study illustrates how the incompleteness and non-uniformity of the fossil record across space and taxa can affect inferences on lineage evolution regarding two fundamental traits, body mass and diet.”

line 484: "rather suggested" is not grammatically correct.

Response: We have removed "rather" from that sentence (line 402).

Again, on line 515 you refer to "our phylogenetic analysis" but you've not actually undertaken such an analysis, at least not one that maximizes likelihood or minimizes character change in a dataset implied by one topological branching pattern relative to others. Instead, you've relied on the topology in Alvarez-Carreterro et al. 2022. That's OK, but stop implying your study is more than what it actually is. Indeed, the caveats listed in this paragraph ("that the same backbone phylogeny will always be recovered...") should've been clear from both the Abstract and initial sentences of the Introduction.

Response: We now refer to "our phylogenetic comparative analysis" in that sentence (line 434), we explain that caveat also in the introduction (lines 75–82), and we use the term "pruned" instead of "removed" in the abstract (line 16) to clarify our methodology.

I hope you'll have a close look at these two papers (already noted above) when you mention "State-of-the-art methods" on line 539:

- Beck RMD, Baillie C. 2018. Proc. R. Soc. B 285:20181632.
- Asher RJ, Smith MR, Rankin A, Emry RJ. 2019. R. Soc. open sci. 6: 190387.

and this paper, one of several since 2006, that examines the reliability of fossilizable data in phylogeny reconstruction (in particular suppdata fig. S9):

- Yuan H, Dickson ED, Martinez Q, Arnold P, Asher RJ. 2024. Proceedings of the Royal Society B.

Response: We thank the reviewer for the references on the "artificial extinction" approach. We have added Beck and Baillie 2018 alongside Asher and Smith 2022 when referring to state-of-the-art methods. The text now reads (lines 471–473):

"Finally, state-of-the-art methods allow the integration of data on extinct and extant organisms [32,54,83,84], increasing the power to explore and elucidate deep-time diversification patterns (as reviewed by [38–40]), …"

line 555: "value of the fossil record is out of the question" means you exclude said "value...". I'd write instead something like "While no one doubts the value of the fossil record..."

Response: We have corrected the sentence as indicated by the reviewer (lines 488–489).

Reviewer #3 (Remarks to the Author):

Rigorous investigations of how biases in taxon sampling affect macroevolutionary inferences from comparative data are incredibly useful and always welcome, in my opinion. As taxon sampling is generally impacted by a complex array of interacting biases, however, it can be difficult to select which exact biases to investigate in a given study—not to mention how to most effectively simulate datasets with those biases. In this regard, starting with empirical data and focusing on well-known biases of the fossil record (e.g., large-bodied, widespread taxa frequenting environments with a high capacity for fossil formation/exposure)—as the authors do here—is certainly an interesting and worthwhile idea.

I have no doubt that this work will prove useful to many macroevolutionary biologists (especially those working with mammals) trying to gauge how the results of their own research are potentially influenced by biases in taxon sampling. Additionally, I could see the study's overall methodology inspiring future work on this important yet generally understudied topic. In particular, testing both the independent and cumulative effects of multiple biases seems like a practical compromise which manages the "combinatorial explosion" of simulation conditions that occur in these kinds of studies while better reflecting the expected complexity of biases affecting empirical datasets. The use of geological/paleontological databases to quantify/simulate the potential for fossilization/subsequent discovery across the globe is also an interesting idea I have not previously encountered (though this may be more common among paleontological research; I generally work with phylogenies primarily consisting of extant taxa).

Response: We really appreciate the reviewer's positive words on our study.

My one major concern with this paper is that it is currently framed as an investigation of how biases in the fossil record may affect macroevolutionary inferences, yet the authors simulate "fossil record biases" by removing tips from a phylogeny consisting solely of extant mammal taxa. While the abstract and introduction of the manuscript make it clear that the authors use data on extant taxa to simulate likely biases in a hypothetical future fossil record, I still expected that the study would implement some kind of strategy for specifically simulating how biased sampling of

non-contemporaneous, extinct taxa (reflecting the typical distribution of fossil data in empirical phylogenies) affect macroevolutionary inferences.

Response: We agree with the reviewer that this is an important limitation of our study. We simulate and analyse fossil samples from a single time point, which certainly departs from regular practice in palaeontological/macroevolutionary studies. We have tried to remark this caveat both in the introduction and discussion of the revised version. The text now reads (lines 83–87):

"We also acknowledge that analysing samples from a single time period departs from common practice in the field, where fossils are generally distributed across multiple time periods throughout a phylogeny, but adding a temporal component to the simulation would imply even stronger assumptions on preservation and sampling processes (e.g., different land configurations or human settlements)."

And later on (lines 455–469):

"In addition, the baseline phylogeny is an ultrametric (extant-only) tree from which we have removed species by pruning the corresponding tips, so our simulated fossil samples represent a single point in time. This contrasts with most studies on fossil remains, where samples of different ages (fossil time-series) are generally analysed, helping trace evolutionary paths [77–80]. We acknowledge that our approach is closer to studies on extant taxa dealing with the effect of incomplete sampling on phylogenetically derived estimates [64–66,81,82], and indeed arrives at the same conclusion: that non-random sampling causes the most skewed estimates. Yet, our incomplete sampling is non-random in a specific way: it reflects biases known to affect the fossil record and further accounts for different preservation scenarios. Therefore, though certainly simplistic, our approach gives some insights into how fossil record biases might distort our interpretation of extinct communities and their trajectories."

Given the overall framing of the current manuscript, I think there are two key issues with the authors' current approach (or at least their interpretation of some results):

1) There are conceptual issues with claiming that these simulations/analyses faithfully represent inferences based on a future fossil record. Under this scenario, more time will have passed by the time these hypothetical future fossils are discovered, such that the apparent distribution of traits (e.g., body size, diet, range size) and species richness (e.g., affecting apparent patterns of lineage diversification) across clades will be different. The extent to which these differences would matter for macroevolutionary inferences is a complex question in and of itself, as it inevitably depends

on the assumed timeframe for fossilization/discovery (I suppose around millions to tens of millions of years reflects the typical timeframe of mammalian fossil records unless one is solely focused on the Pleistocene) as well as the evolutionary dynamics of trait evolution/lineage diversification processes across the mammal phylogeny.

Response: We agree with the reviewer that the absence of an explicit temporal dimension is one of the main limitations of our framework, a simplification that necessarily reduces realism but also frees us from further assumptions on how preservation and sampling biases might change through time.

2) Extant taxa occur at a single point in time corresponding to the maximum height of a phylogeny, while fossil taxa are generally distributed across multiple time periods throughout a phylogeny. As such, we generally expect that removal/biased sampling of fossil taxa will have distinct impacts on macroevolutionary inferences from phylogenetic comparative data compared to removal/biased sampling of extant taxa.

Response: The reviewer is right, and we have tried to remark the caveats of our study as indicated above, in our previous responses.

Accordingly, I have three alternative suggestions for how the authors might revise their manuscript to better address/acknowledge these issues (generally ordered from most ideal to most practical):

1) Explicitly simulate fossilization and/or macroevolutionary processes some amount of time into the future to better address the questions posed in the introduction of the manuscript or at least gauge how much such processes could impact the overall conclusions of this study. While I think this option would greatly strengthen the manuscript, it would also introduce numerous additional complexities/assumptions to the authors' simulation pipeline and seems potentially impractical/beyond the scope of the current work.

2) Reframe the manuscript as being focused on biases in sampling of extant taxa in comparative studies, rather than fossil data. In this context, the fossilization component instead serves as a kind of foundation for identifying common, relatively universal biases that are likely to affect sampling of extant taxa. Admittedly, the preservation bias simulations would appear less well-

motivated in this context, and I do think that these simulations are an interesting and unique aspect of the current manuscript.

3) Keep the current framing largely intact while explicitly acknowledging this important caveat with the study's simulation methods/analysis. I think this would reduce confusion among readers as well as help other researchers better contextualize/qualify the study's overall results/conclusions. Admittedly, I am not a paleontologist or mammalogist, so I may be unaware of some concrete, empirically grounded reasons to suspect that the aforementioned issues are inconsequential for the overall results/conclusions of the study. Nonetheless, I think such reasons need to be explicitly stated if they exist, as this seems highly counterintuitive to me given my understanding of macroevolutionary modeling and the general timeframe of mammalian evolution.

Response: We thank the reviewer for precisely noting the caveats of our approach and for proposing ways to deal with them while maintaining the usefulness/validity of our study. In line with the reviewer's reasoning, we have followed this third alternative, highlighting the caveats and reframing the study to make clearer what we have done and how our findings can still inform on the impact of fossil record biases on macroevolutionary estimates. To that end, we have reorganized the introduction and discussion sections as indicated in previous responses.

Outside of this one major concern, I think this study presents some unique and innovative strategies for simulating/investigating the impacts of sampling biases on macroevolutionary inferences and contains a lot of useful, generalizable information/insights. I provide some additional, more minor feedback on a line-by-line basis below:

L25-27: Perhaps this is only semantic/philosophical, but I would argue that comparative biology is another major, albeit indirect, source of empirical information on how life evolved on Earth.

Response: The reviewer is right, as also noted by Reviewer #2, the fossil record is not the only empirical evidence on how life evolved on Earth. We have replaced "the only" by "primary" in that sentence.

L27: "riche" should be "rich"

Response: We thank the reviewer for spotting that, we have now corrected the typo.

L42-44: While the authors are correct that the impact of fossil record biases on macroevolutionary inferences (particularly with respect to trait evolution) is understudied, I think it is worth mentioning here that there is relevant previous work on how inclusion of fossil data (e.g., Slater et al 2012, https://onlinelibrary.wiley.com/doi/full/10.1111/j.1558-5646.2012.01723.x; Puttick 2016, https://onlinelibrary.wiley.com/doi/full/10.1111/j.1558-5646.2012.01723.x; Schnitzler et al 2017, https://drive.google.com/file/d/16qoCwyIXOKNhVfQPPeJkCOvQQE2RIkBR/view) as well as biased sampling of extant taxa (e.g., Ackerly 2000, https://onlinelibrary.wiley.com/doi/abs/10.1111/j.0014-3820.2000.tb00694.x; Marcondes 2019, https://pmc.ncbi.nlm.nih.gov/articles/PMC6791351/; Johnson et al 2020, https://onlinelibrary.wiley.com/doi/full/10.1111/geb.13185) affect trait evolution inferences from phylogenetic comparative data.

Response: We thank the reviewer for the references on this topic. We have included them in the revised version, the first three in the introduction (line 55) and the last three in the discussion (lines 295, 464).

L181-182: Brownian Motion refers to a specific stochastic process which is only applicable to continuously varying quantities—it does not apply to discrete trait evolution models, which are instead based on continuous time Markov Chains (CTMCs). I think the most clear way to state the authors' methods here is that they fit Brownian Motion models to the continuous trait data and ER/ARD models to the discrete trait data either without or with one of Pagel's three tree transformations. This is because one is essentially still fitting the same basic models to the data even with Pagel's tree transformations in this context—these transformations only serve to squash/stretch the branches of the phylogeny to further improve the model's fit to the data.

Response: We thank the reviewer for the precise description of the models of trait evolution. We have modified the text accordingly, now it reads (lines 613–629):

"To assess the mode of trait evolution along the phylogeny, we fitted likelihood models for both continuous and discrete traits using the *fitContinuous* and *fitDiscrete* functions (respectively), and compared them by means of the Akaike information criterion using the *aicw* function, all from the 'geiger' package (v.2.0.11) [97]. For continuous traits, we compared Brownian motion (BM) [98] and Pagel's tree transformation models [94]: lambda (λ, covariance among trait values for species), kappa (κ, punctuated/speciational evolution), and delta (δ, time-dependent evolution). Both λ and κ range

from 0 to 1, while δ can take any value. When any of the parameters is 1, the model equates to BM. A λ of 0 means that the trait has evolved independently of the phylogeny whereas a κ of 0 indicates that changes only occurred at branching (speciation) events. δ values below or over 1 point to slowdown or acceleration of evolution towards the present, here largely corresponding to 'early burst' (EB) [99] and 'Ornstein Uhlenbeck' (OU) [100] models of trait evolution, respectively. For discrete traits, we evaluated models under both equal and all different transition rates between character states (ER and ARD, respectively), either specifying or not (null model) one of the three Pagel's tree transformations."

We have also adjusted text in results and discussion sections (e.g., lines 227, 372), as well as Fig. 4 and Supplementary Fig. 9 and 15.

L188-189: I think it is worth mentioning here that delta<1 corresponds to the popular "early burst" (EB) model of trait evolution (Harmon et al 2010, https://academic.oup.com/evolut/article-abstract/64/8/2385/6854028?redirectedFrom=fulltext&login=false) while delta>1, at least for ultrametric data (as is the case in the current manuscript), corresponds to the popular "Ornstein Uhlenbeck" (OU) model of trait evolution (at least assuming a single trait optimum applies to the entire phylogeny; Blomberg et al 2003, https://academic.oup.com/evolut/article-abstract/57/4/717/6756141?redirectedFrom=fulltext&login=false). While I think Pagel's tree transformations are conceptually and practically useful, they have fallen out of favor compared to the increasingly ubiquitous Brownian Motion/EB/OU model selection framework in my experience. Making this explicit connection will enable more readers to easily relate the results of this paper to their own research. Notably, however, revising the study to involve non-ultrametric phylogenies with extinct taxa will render delta>1 no longer equivalent an OU model. In that case, delta>1 is best thought of as modeling a "late burst" in contrast to an "early burst".

Response: We thank the reviewer for this clarification. We have added the suggested information to the methods section, as indicated in our previous response.

L263: This is only a minor point, but I do wonder to what extent the overdispersion of medium sizes may be a simple consequence of the ordinal nature of a discretized measurement of body mass. In other words, you may inevitably expect medium sizes to be phylogenetically overdispersed because small-bodied lineages generally have to pass through medium-bodied phase to become large-bodied and vice versa. To that end, I also think it would be interesting

(albeit not strictly necessary) to explore how the results of the discrete trait evolution analyses differ if one fits ordinal models of discrete trait evolution (i.e., forbidding direct transitions from small to large-bodied to states; these are termed "meristic" models in geiger) in addition to ER/ARD models (the authors could also do something similar for herbivory/omnivory/faunivory). Unfortunately, I cannot think of a straightforward way to incorporate the natural "order" of states into metrics of phylogenetic under/overdispersion. Perhaps seeing how the NRI results change when treating body size as a binary character could shed some light on this issue.

Response: In line with the reviewer comment, we do think that the overdispersion of medium sizes in baseline data is related, at least to some extent, with the way we defined the size categories. The medium category is very broad (500.1 g – 50,000 g) in contrast to the small and large categories. We adopted this classification as a compromise between the usual definitions of small and large mammals and the resulting number of species per category, but we acknowledge that its amplitude can contribute to the observed pattern. In that respect, we also tried an alternative classification with four categories (0 – 100 g, 100.1 – 1,000 g, 1,000.1 g – 10,000 g, > 10,000 g) and the third category largely behaved as the medium we finally used: it was overdispersed in the baseline phylogeny and turned into random or clustered patterns across simulated samples. This result would be compatible with our interpretation that the broadness of those categories (including diverse species across the mammal tree) might explain the observed patterns.

In the case of diet, while omnivory might also be seen as the path between herbivory and faunivory, it adjusted to clustering in the baseline tree. In this case, the number of species per category was more even than for body mass (see Supplementary Fig. 3).

To model the evolution of the traits, we also considered the meristic model because the sequential transition between states seems more logical for both body size and diet (in biological terms) than ER or ARD models. However, we ended up not using it for practical reasons, because at least one of the categories was frequently lost in the simulated samples, causing errors when running the models (at least in the way we implemented them). Besides, the meristic model can be considered a particular case of the ARD model, where transitions that do not pass through the intermediate category are not allowed. So, in theory, a meristic model could still be inferred for the data through the ARD implementation.

In line with the reviewer, we do not see a straightforward way of considering the sequential transition between states when estimating NRI.

L303-304: "increased respect" should be "increased with respect"

Response: We have corrected the sentence.

L451: “Random evolution” is a somewhat vague interpretation of Brownian Motion-like evolutionary processes. More specifically, Brownian Motion (at least with no tree transformation) corresponds to what would be expected under pure drift/neutral dynamics OR selection with randomly varying direction/strength across time/lineages.

Response: Following the previous reviewer’s suggestions, we have replaced “random” by “stochastic”.

I sincerely hope the authors find this feedback useful!

Response: We sincerely appreciate the careful revision of our work and the constructive comments provided by the reviewer.

Reviewer #3 (Remarks on code availability):

I have not investigated the authors' code in detail, but skimmed through some of it. In general, everything looked organized and straightforward to understand/reproduce. Additionally, the authors do a thorough job of describing their methods in the paper itself.

Response: We thank the reviewer for checking our code, and for their positive words on our code and methods.

RESPONSE TO REVIEWER COMMENTS

We thank the reviewers for their availability to go through the revised version of our manuscript. We sincerely appreciate their positive words on our work and their detailed comments, which helped us to further improve the quality and clarity of our text. Please find below our point-by-point response (**in bold**) to the reviewers' comments, where line numbers correspond to the revised version with tracked changes.

Reviewer #1 (Remarks to the Author):

I thank the authors for their thoughtful revision and responses to my comments. Although maintain that the body size filter is too harsh – and that the fossil record of small vertebrates is better than is being modeled – I agree that the broadest finding (that aspects of evolutionary history are likely erased by the fossilization process and taphonomic biases) is well-made, and…if not strictly novel in a paleontological sense…is nicely demonstrated in these experiments. I only have a few additional comments that the authors may wish to take on board, or ignore as they see fit.

Response: We thank the reviewer for their positive feedback and additional suggestions on our revised manuscript. As the reviewer indicates, our general findings can serve as an illustrative example of the potential effects of fossil record biases on deep-time macroevolutionary inferences.

1. Following from my main point above, it might be worth emphasizing somewhere in the discussion that efforts to reconstruct the small vertebrate record – specifically by focusing on unusual, but widespread taphonomic windows like regurgitate deposits – should arguably be a bigger priority in both paleontology and evolutionary biology. I particularly like the 2010 paper by Rebecca Terry in Proceedings B, which illustrates not only that these deposits preserve the small mammal record extremely well, but also that they're more widespread that commonly appreciated. In the scenario the authors are considering…i.e., trying to reconstruct evolutionary history using an imagined fossil record of extant mammals, it's doubtless that lagerstätten, regurgitates, and even cave deposits where many common taphonomic biases do not operate, would be used. In turn, the author's findings illustrate that – if we're hoping to understand actual evolutionary history – devoting more resources to studying these deposits is crucial.

Response: We fully agree that unusual preservation windows (such as lagerstätten, regurgitates, and cave deposits) provide critical access to the small mammal record, and we thank the reviewer for the further information on this topic. In particular, the study by Rebecca Terry (https://doi.org/10.1098/rspb.2009.1984) highlights how historical and subfossil skeletal samples can inform on the structure of modern small mammal communities, giving insights into their recent evolution. However, because the scope of our study was more general, we have not added this point on exceptional deposits to the discussion.

2. A related point, is that taphonomic processes are not exactly operating in the present day as they have in the past, and this might be worth thinking about (again, in context of the exercise the authors are walking through: modeling a future fossil record). It's probably only worth a brief mention, but Plotnick and Koy (2020) in Anthropocene ("The Anthropocene fossil record of terrestrial mammals") has some valuable insights.

Response: We agree with the reviewer that taphonomic processes can also vary across time, as we mention in the introduction. We thank the reviewer for the reference, which we have added to the text (lines 39—40):

"However, this rich archive is inherently incomplete and non-uniform across space, time, environments, and taxa [1–4]."

Reviewer #3 (Remarks to the Author):

The authors have done a fantastic and thorough job addressing reviewer feedback, demonstrating careful attention to detail and thoughtful consideration of each comment. Even in cases where the authors did not substantially alter their work in response to feedback (e.g., not adding a temporal component to their simulations, not treating diet/discretized body mass as ordinal variables), they nonetheless provided clear justifications for their decisions.

Overall, I think this is a quality and insightful contribution to the literature on how taxon sampling biases affect macroevolutionary inferences. Further, the heavy reliance on empirical data and trends in the authors' simulation pipeline appears rather novel and interesting (though there

could be some literature I am unaware of). Even with the caveats of the study (which I think the authors do a much better job of acknowledging in their revised manuscript!), I agree with the authors that their work provides an excellent set of "baseline" results which can be further interrogated going forward. In any case, I think this paper has value not only in the results it directly provides, but also the simulation strategies it might inspire others to do to gain further clarity on these topics.

Response: We sincerely appreciate the reviewer's positive feedback on our work and our revised manuscript.

I do not have any remaining major criticisms of the paper, though I did find the wording of some sentences vague or unclear, which I detail below (line numbers based on clean copy):

L36: This is super minor, but I found myself getting tripped up by "while" in this sentence-- "though" might fit better?

Response: We thank the reviewer for pointing this out, the word has been corrected.

L41-42: I might suggest rewording as "...with a general consensus that combining data from fossil and living taxa increases phylogenetic accuracy..."

Response: We thank the reviewer for the suggestion, the sentence has been reworded as indicated.

L46: I think the first sentence of this paragraph is a little vague (what question exactly, and what does your approach differ from?). I would simply say something along the lines "Here, we use data on extant taxa to explicitly simulate the effects of fossil biases on macroevolutionary inferences..."

Response: We agree that the sentence was not clear enough, and we thank the reviewer for the proposed alternative. We were meaning that our approach differs from those mentioned in the previous paragraph to address the question on the effect of the fossil record biases on macroevolutionary inferences. We have rephrased it accordingly (lines 62—63):

"Here, we use data on extant taxa to explicitly simulate the effects of fossil record biases on macroevolutionary inferences."

L54: "distribution" should be plural here, I believe.

Response: The reviewer is right; it has been corrected.

L67-68: Apologies if I'm getting nitpicky here, but I'm not sure "conservative" is quite the right word here--I think a clearer way to explain your point would be to simply say you're trying to isolate the effects of fossil records biases on macroevolutionary inferences. While it's unrealistic to assume the phylogenetic reconstruction would be identical, re-estimating the phylogeny would not only be impractical for the scope of this study, but also leave it unclear whether downstream effects on macroevolutionary inferences were driven primarily by inaccurate phylogenetic reconstruction (a much more well-studied topic) or fossil record biases.

Ah, I see you elaborate on this point more in the discussion! Well, I think it works in that context with the subsequent sentences--I might suggest just making the point a bit more explicit in the introduction.

Response: We thank the reviewer for their suggestions to better explain/justify why we did not include a phylogenetic reconstruction step in our approach. We have included the proposed modification in the introduction. Now it reads (lines 83—86):

"We acknowledge this is rather unlikely [54,55], but our aim here is to isolate the effect of fossil record biases on subsequent evolutionary estimates from the distortion caused primarily by inaccurate phylogenetic reconstruction."

Also, some sources that might strengthen some of your points on avoiding phylogenetic reconstruction/lacking temporal realism: https://doi.org/10.1093/sysbio/syq098, https://doi.org/10.1002/ecy.2788

To be clear, I think the caveats here are important to explicitly state and talk about, as I still think they could have major effects on the results (a point I'm sure you all agree with in any case!). Further, these papers only cover rather narrow aspects of macroevolutionary inference (regression, phylogenetic diversity, phylogenetic signal...). That being said, I nonetheless think these citations could provide readers with a bit more confidence in the "gist" of your results by

showing how some common phylogenetic comparative analyses appear rather robust to error in tree topology/branch length estimates (at least to a degree).

Response: We completely agree with the reviewer that the caveats of our approach are important and should be clearly stated, and we tried to do so in our previous revision. We also thank the reviewer for the additional references in support of our phylogenetic comparative approach, which we have included in the text as follows (lines 442—444):

"Nonetheless, some common comparative methods as those we applied here (e.g., phylogenetic signal) appear to be robust to tree misspecification, at least in terms of general trends [76,77]."

L76: "...how these traits are distributed across the phylogeny..." is the more conventional phrasing, in my experience.

Response: We agree with that; it has been corrected.

L204: "...it was best fit by..." is more common/clear phrasing than "...it best adjusted to..." in my experience.

Response: We thank the reviewer for the correction, which we have implemented.

L210: "none tree transformation" should be "no tree transformation" here, right?

Response: Right; it has been corrected.

L427-428: I would instead write "...instead of focusing on uncertainty around individual estimates..." as this would apply to both model selection and parameter uncertainty. Confidence intervals around values makes it sounds like you're only referring to parameter uncertainty.

Response: We thank the reviewer for pointing out the inaccuracy of that sentence; it has been corrected as suggested.
